# Practical Application and Methodological Considerations on the Basics of Sports Nutrition in Basketball: A Comprehensive Systematic Review of Observational and Interventional Studies

**DOI:** 10.3390/nu15204484

**Published:** 2023-10-23

**Authors:** Paulina M. Nowaczyk, Jakub Adamczewski, Krzysztof Durkalec-Michalski

**Affiliations:** 1 Department of Sports Dietetics, Poznań University of Physical Education, 61-871 Poznań, Poland; adamczewski@awf.poznan.pl; 2 Sport Sciences–Biomedical Department, Charles University, 162 52 Prague, Czech Republic; 3 Centre for Sport Research, Deakin University, Burwood, VIC 3125, Australia

**Keywords:** team sports, sports dietetics, nutritional support, diet evaluation, hydration practices, eating habits

## Abstract

The current systematic review (PROSPERO registration no. CRD42022334707) of observational (OS) and interventional studies (IS) aimed at evaluating the state of scientific knowledge on the basics of sports nutrition, framing discipline-specific dietary recommendations, and indicating potential directions for future studies in various age, experience level, and able-bodied abilities groups of basketball players (BP). A systematic search of PubMed, SPORTDiscus, and Web of Science ended on 20 December 2022. Records were excluded if reporting studies on animals, sport disciplines other than basketball, or supplementation protocols other than those related to macronutrients and hydration manipulations. Risk of bias (RoB) was evaluated using Cochrane RoB_2 tools, ‘JBI checklist for prevalence studies’, and ‘Quality assessment tool for before-after (pre-post) studies with no control group’. The relevant data was synthesized in tables and a narrative review was performed. Seventy-two records were included (2581 participants): 63 were on able-bodied BP (2433 participants) and 9 on para-athlete players (148 participants); 45 records were OS and 27 IS. The review disclosed widespread poor nutritional habits and knowledge and shortages in applying adequate nutritional and hydration practices in BP. Moreover, the systematic review revealed the lack of a sufficient number of investigations delivering reliable proof for framing discipline-specific and evidence-based recommendations on the basics of sports nutrition in basketball.

## 1. Introduction

Basketball is a court-based team sport, one of the most popular sports disciplines in the world for both males and females and across all levels of competition and age groups [1]. Each basketball team consists of five players, playing in different positions (e.g., centers, guards, and forwards) [1]. Basketball match playing time depends on the level of competition and may last from 10 to 12 min per quarter (QR) with a total of four QRs per game [2]. Furthermore, wheelchair basketball is also one of the most widespread sports for para-athletes, practiced in nearly 100 countries around the world [3]. Rules of matches are almost the same as those applicable in the competition of able-bodied basketball players according to the rules of the International Basketball Federation (FIBA; e.g., rules related to playing time, size of the court or the ball) [3]. Nevertheless, in wheelchair basketball, there are some additional requirements to follow, related mainly to the equipment allowing athletes to participate in basketball practice [4].

Basketball is a high-impact sport, and active participation in basketball match play requires performing fast and short accelerations and decelerations, explosive changes of directions, jumps, as well as physical contacts with other opponents [3]. Still, the frequency of these efforts depends mostly on players’ positions [1]. The matches that last 40 min generate usually 5–6 km distance by both male and female basketball players (BP), with average exercise intensities above the lactate threshold, and 85% of maximal heart rate (HR_max_) [2]. Although basketball is not a typical endurance-based sport, it is crucial to maintain high contributions of both aerobic and anaerobic metabolic pathways covering energy requirements during the game/training [1]. According to the literature, the average maximal oxygen uptake (VO_2max_) is 44.0–54.0 mL∙kg^−1^∙min^−1^ in female BP and 50.0–60.0 mL∙kg^−1^∙min^−1^ in male BP [1]. High-intensity efforts cover up to 65% of the total active play-time during basketball practice, and they are associated with covering energy demands mostly by glycolytic pathways [1,5]. Thus, increased liver and muscle glycogen stores obtained by high carbohydrate (CHO) intake may support performance and help delay exercise-induced fatigue [6], especially in the last QR of the match, when limited CHO availability may result in less involvement of the players in active and effective efforts [1]. CHO intake should be individually adjusted to one’s requirements arising not only from anthropometric characteristics and playing position [2] but also training goals, training macrocycle period, and schedule to maximize physical and cognitive performance or optimize proper adaptation and recovery after exercise [4,7].

Each sport discipline differs in the types and specificity of efforts, energy pathways, exercise duration, and environmental conditions in which training and/or competition are undertaken. Therefore, discipline-specific nutritional recommendations are required. It should be mentioned that there is a relatively large body of evidence regarding nutritional recommendations for team sports such as football/soccer [6,7] (also regarding gender-dependent differences within these disciplines) [8,9] or rugby [10]. However, basketball seems to be neglected in this respect.

Thus, the current systematic review aimed to (1) evaluate the current state of scientific data on the basics of sports nutrition in basketball; (2) frame discipline-specific dietary recommendations; as well as (3) indicate potential directions for future studies within this area. To obtain the assumed aims, the following prosecution was planned to undertake: (1) evaluation of habitual energy and macronutrients intake and hydration strategies/hydration status in BP at a different age, level of training experience, gender, or level of full-body abilities (able-bodied BP and para-athlete players); (2) identification of possible reasons for poor nutritional value and quality of diet in BP based on eating behaviours and nutritional knowledge (NK) evaluation; and (3) consolidation of summaries of experimental protocols and evaluation of the effectiveness of up-to-date dietary interventions related to hydration strategies and macronutrients manipulations undertaken in BP. Recently, two reviews on in-season nutrition strategies to enhance recovery for BP [11], or ergogenic and/or micronutrients’ supplementation interventions in basketball [12], were published. However, the scopes of the aforementioned reviews do not cover the scope of the current systematic review. Moreover, the first mentioned is a narrative review [11]. Thus, the current systematic review is a unique, comprehensive elaboration concerning both the scope of the paper and coverage of various subgroups of BP and the type of studies’ designs considered to accomplish the above stated objectives.

## 2. Materials and Methods

### 2.1. Study Eligibility

The systematic review was conducted according to the *Preferred Reporting Items for Systematic Review and Meta-Analyses* (PRISMA) guidelines [13] (see PRISMA checklist, Supplementary Table S1 A,B) and was registered prospectively with the PROSPERO database (no. CRD42022334707). Observational (OS) and interventional (IS) human studies in English and Polish were included in this review. The review questions and studies’ eligibility criteria were determined according to PICOS (*Population, Intervention, Comparison, Outcomes, and Study Design*) criteria [14]. The *Population* included male and female BP at different ages and levels of training experience. Studies on para-athlete players (i.e., wheelchair and deaf players) and able-bodied athletes were taken into consideration. Regarding OS, the study must have described the habitual (1) energy, carbohydrate (CHO), protein (PRO), or FAT intake; (2) hydration behaviours and hydration status; or (3) eating behaviours and/or NK. Concerning IS, the *Intervention* must have employed a single acute or chronic dietary intervention protocol implementing supplementation and/or manipulation in macronutrients (CHO, PRO, FAT/fatty acids <FA>) intake or must have been a hydration/dehydration strategy. In relation to *Comparison*, the OS could have been cross-sectional single time-point descriptions or multiple time points within/between athletic season comparisons. Regarding *Study Design* of IS, the protocol for the current systematic review permitted non-, single-, and double-blinded designs; single- (pre-post/before-after comparisons); and multiple-arm (placebo-controlled and non-placebo-controlled; randomized and non-randomized, parallel-group and cross-over) studies. A wide range of *Outcomes* was considered, including body mass (BM) and composition and indices related to physical capacity and discipline-specific performance, adaptation and recovery, cognitive performance, hydration status, or sleep quality. The studies were excluded if reporting on animal studies or human studies on supplementation protocols other than those related to macronutrients and hydration manipulations.

### 2.2. Search Strategy

**Figure 1:**
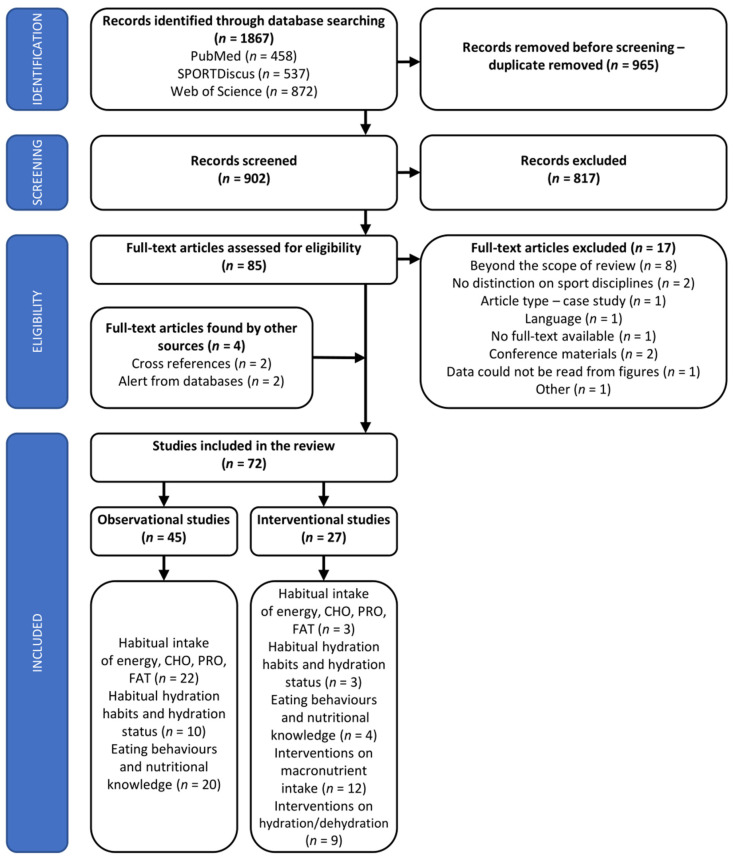
PRISMA flow diagram of studies included in the systematic review. Several studies investigated more than one of the subject areas of the review. Summing the number of studies will lead to duplication and not accurately reflect the total number of included studies.

A systematic search of three databases (PubMed, SPORTDiscus, and Web of Science [WoS]) was conducted (P.M.N., J.A.) from 30 May 2022 to 20 December 2022 to identify all relevant articles for the current study. No limitations regarding the date of publication were implemented. The search terms ‘energy intake’, ‘energy’, ‘energy value’, ‘energy availability’, ‘carbohydrate’, ‘fat’, ‘fatty acids’, ‘protein’, ‘fluids’, ‘eating behaviours’, or ‘nutritional habits’ were individually contacted with ‘basketball’. ‘All fields’ (WoS, SPORTDiscus) or ‘title and abstracts’ (PubMed) were searched. The following filters were set: species—humans (inclusion), languages—English, Polish (inclusion), type of article—review (exclusion). Following the removal of duplicates (Figure 1), the titles and abstracts of retrieved articles were independently screened by two researchers (P.M.N., J.A.) to assess their eligibility for inclusion in the current review. Records that had unclear suitability were included at this stage, and the final decision was reached after reading the full text. Any disagreements regarding study eligibility were resolved through discussion and consensus with the third reviewer (K.D.-M.).

### 2.3. Data Extraction and Synthesis

All the data were first extracted independently by two reviewers (P.M.N., J.A.), then discussed by the reviewers, and eventually checked for correctness and clarity by a third reviewer (K.D.-M.).

For preliminary alignment of the gathered data, general characteristics of included studies were processed via extracting and tabularizing the following data: surname of the first author (or two authors), location, type of the study and study design, sample size, gender, age, experience and level of basketball training, and occurrence of disabilities (Supplementary Table S2). Further, the data were organized into five main subject areas and synthesized in the relevant tables: (1) habitual energy and macronutrient intakes (first author [or two authors] and publication year, dietary evaluation method, sample size and gender, age, BM, energy value, macronutrient intake, season/training macrocycle time point; Table 1); (2) habitual hydration strategies and hydration status (first author [or two authors] and publication year, sample size and gender, age, BM, environmental conditions, duration and type of exercise, fluid intake, indices of hydration status; Table 2); (3) dietary interventions related to hydration/dehydration strategies (first author [or two authors] and publication year, study design, sample size and gender, age, dietary intervention description, summary of experimental procedure and outcomes; Table 3); and (4) dietary interventions related to macronutrient manipulations (same data extracted as for latter mentioned area, Table 4); and (5) NK and eating behaviours (taking into account a large diversity of methods for eating habits and NK evaluation and a great range of measured outcomes, solely the narrative synthesis was performed within this area).

The data extracted from IS were insufficient for meta-analysis due to diversity in studies’ protocols and measured outcomes and in general a low number of relevant studies. Therefore, for all the subject areas being discussed in the review, a narrative synthesis was conducted.

### 2.4. Assessment of Studies’ Quality

Due to a wide range of study designs included in the systematic review, various tools for risk of bias (RoB) assessment needed to be employed, with Cochrane tools, if applicable, being the most preferable. For randomized IS the revised Cochrane RoB tools for randomized (RoB 2) parallel groups [71,72] or cross-over designs [73] were implemented. For single-arm (pre-post) IS the ‘Quality assessment tool for before-after (pre-post) studies with no control group’ developed by the National Heart, Lung and Blood Institute [74] was implemented. While the quality of observational cross-sectional studies was assessed with ‘The Joanna Briggs Institute critical appraisal checklist for studies reporting prevalence data’ (‘JBI checklist for prevalence studies’) [75]. The two latter tools were selected based on a recent review on methodological quality assessment tools for medical studies [76]. Regarding observational cross-sectional studies related to energy and macronutrients intake or hydration strategies and hydration status, which were evaluated via ‘JBI checklist for prevalence studies’ [75], the question related to statistical analysis was discounted, while single-point data were extracted for the systematic review. RoB was first evaluated by one author (P.M.N.) and further discussed and revised by another author (K.D.-M.).

## 3. Results

The literature search initially identified a total of 1867 potential records, of which 902 remained for screening after duplicates removal (Figure 1). After title and abstracts screening, 85 articles were evaluated for inclusion/exclusion criteria. Finally, 72 full-text articles (of which 63 were papers on able-bodied BP [on 2433 athletes] and 9 were para-athlete BP [on 148 athletes]) on 2581 participants met the inclusion criteria and were included in the systematic review. Forty-five studies were OS, and the remaining 27 studies were IS (Supplementary Table S2). Twenty-five studies (22 OS and 3 IS) reported on habitual intake of energy, CHO, PRO, and FAT (Table 1); 13 studies reported on habitual fluid intake and hydration status (10 OS and 3 IS; Table 2); 9 papers were interventions related to dehydration/hydration status (Table 3); 12 were IS on macronutrients manipulations (Table 4); and 24 studies referred to eating habits/behaviours and NK (20 OS and 4 IS). Some of the studies were included in more than one subject area of the current systematic review. The baseline data from the few IS that referred to habitual energy and macronutrient intake [15,37], eating behaviours [22], or habitual fluid intake and hydration status [40,42,49] were also included in the tables related to OS. These studies are marked with ‘^§^’ in the relevant tables.

Within OS, 2 studies were published between 1980 and 1989, 4 studies between 1990 and 1999, 4 studies between 2000 and2009, 22 studies between 2010 and 2019, and 13 studies between 2020 and 2022. Within IS, 1 study was published between 1990 and 1999, 5 studies between 2000 and 2009, 17 studies between 2010 and 2019, and 4 studies between 2020 and 2022.

### 3.1. Study Quality and Risk of Bias

RoB was evaluated separately for each of the five subject areas of the review and within each area with the use of relevant tools corresponding to the type of the study and/or its design.

RoB for studies reporting on habitual energy and macronutrients intake was evaluated via the ‘JBI checklist for prevalence studies’ [75]. Regarding three questions related to study sample, namely (1) the appropriateness to address the target population; (2) the appropriateness of the selection process, and (3) the adequateness of sample size; as many as 32 [15,18,23,24,26,27,29,30], 32 [15,18,23,24,26,29,30,39], and 36% [15,18,23,24,26,27,29,30,39] of the included studies (25 studies) were rated ‘no’, respectively, and the next 12 [25,31,39], 8 [27,31], and 8% [25,31] were categorized as ‘unclear’ (see Supplementary Material S3A,). The remaining studies were evaluated as ‘yes’. As many as 48% of studies did not describe study settings and subjects with a sufficient level of detail [15,18,19,20,22,23,24,26,27,28,29,30], and one study (4%) was rated ‘unclear’ in this respect [36]. In 40% of studies, data analysis did not cover the study group sufficiently, and they were rated as ‘no’ [15,16,18,23,24,25,26,27,29,39], while the next 12% were rated as ‘unclear’ in this respect [28,31,32]. As many as 24% of studies did not use valid methods for the identification of the condition (e.g., subjects were not instructed about keeping food diaries or diet recalling, and dietary evaluation was performed based on solely single-day recall) [15,16,19,21,31,32], and in the next 12% of studies the descriptions of methods for diet evaluation were ‘unclear’ (e.g., lack of clear description of study participants’ familiarization with diet evaluation methodology) [30,34,39]. Studies that used food frequency questionnaires (FFQ) for diet evaluation [18,33] utilised validated tools. In one of the included studies, there was a great risk for lack of a standard way for measuring diet across all participants [31], due to a lack of any instructions given to the participants. In the next 32% of studies, this point seems to be ‘unclear’ [15,16,19,30,32,34,37,39]. In general, the adequacy of the response rate was the best-rated of all evaluated aspects. Still, one study (4%) was evaluated negatively [32] due to nearly 10% of participants who did not complete the dietary recall correctly (and taking into account the baseline compliance rate, which was 71%), and 12% of studies were evaluated as ‘unclear’ [27,37,39]. As many as 16% of studies (four studies) were rated ‘yes’ for all the questions [17,33,35,38]. These studies can be evaluated as having ‘low’ RoB.

RoB for studies reporting on habitual hydration strategies and hydration state was evaluated via the ‘JBI checklist for prevalence studies’ [75]. As many as 31% of the studies were ranked negatively regarding appropriately addressing the target population [40,41,44,49], and 8% (one study) was perceived as ‘unclear’ in this respect [42] (Supplementary Material S3C,D). Fifty-four percent of studies were ranked as ‘unclear’ regarding the procedure of sampling of study groups [40,41,42,44,46,49,50], and in 31% of the studies, the sample size seemed to be too low from the point of view of the stated studies’ aims [40,41,44,49]. In more than half of the studies (54%), the descriptions of study participants and settings were assessed negatively [41,43,44,45,46,49,50], and in the next 15%, this point was ‘unclear’ [32,48]. Data analysis was perceived as insufficiently covering the identified sample in 23% of studies [42,43,49] and as unclear in 38% of studies [32,40,41,44,48]. One study was evaluated as using an invalid method for the identification of outcomes and simultaneously ‘unclear’ regarding the use of a standard way for measuring outcomes across all the participants [32], while all the remaining studies were categorized as using valid methods and standard methodology for the entire sample [40,41,42,43,44,45,46,47,48,49,50,51]. The response rate was rated as inadequate in one study [77] and ‘unclear’ in two (15%) other studies [46,48]. Solely the study by Vukašinović-Vesić et al. [51] was rated ‘yes’ for all the questions and thus has been recognized as having ‘low’ RoB.

RoB for studies reporting on hydration/dehydration strategies was evaluated using Cochrane RoB 2 tools [72,73]. Out of nine studies, two studies [49,53] were evaluated as possessing ‘some concerns’ and seven studies [42,52,54,55,56,57,58] as having ‘high’ RoB (Supplementary Material S4A,B). 

RoB for studies reporting on dietary manipulations in macronutrient intake was evaluated using Cochrane RoB 2 tools [72,73] or ‘Quality assessment tool for before-after (pre-post) studies with no control group’ [74]. Out of nine randomized studies, four studies [59,60,69,70] were rated as exhibiting ‘some concerns’ and five studies [61,62,63,67,68] as having ‘high’ RoB (Supplementary Material S5A,B). None of the three included single-arm studies [64,65,66] was rated positively in all of the evaluated domains. The share of scores in particular domains is shown in Supplementary Material S5C,D.

Regarding the area of eating behaviours and NK, three different tools for RoB evaluation were used depending on the type of the study; they were (1) ‘JBI checklist for prevalence studies’ [75] for cross-sectional observations [19,20,28,29,32,38,39,78,79,80,81,82,83,84,85,86,87,88,89,90]; (2) ‘Quality assessment tool for before-after (pre-post) studies with no control group’ [74] for single-arm IS [22,37,91]; and (3) Cochrane RoB 2 [73] for interventional randomized cross-over studies [92]. Regarding cross-sectional observations and questions related to the study sample: (1) addressing the target population, (2) selection process, and (3) sample size, as many as 20 [29,79,83,90], 15 [29,39,79] and 35% [29,39,79,83,87,88,90] of included studies were evaluated negatively, and the next 15 [39,87,88], 15 [78,83,90], and 5% [84] were categorized as ‘unclear’ (Supplementary Material S6A,B), respectively. In 40% [29,39,83,89,90] of studies, the descriptions of study settings and subjects were insufficient, and in the next 5% [32] they were ‘unclear’. One study used an invalid method for identification of the outcomes [77], and in 40% of them the validity of methods was ‘unclear’ [39,79,80,81,85,87,88,89]. Standardization of measuring outcomes was ‘unclear’ in 25% of studies [32,39,85,88,89], and the appropriacy of statistical analysis was recognized as ‘unclear’ in 10% of them [39,86]. The response rate was inadequate-rated as ‘no’ in 10% of studies [32,87] and ‘unclear’ in 20% [39,83,84,90]. Solely one study [38] was evaluated ‘yes’ for all the questions and could be perceived as having ‘low’ RoB. None of the single-arms IS [22,37,91] could be perceived as having ‘low’ RoB (Supplementary Material S6 C,D), while the sole randomized cross-over study [92] identified in this subject area was rated as having ‘some concerns’ regarding RoB (Supplementary Material S6 E).

### 3.2. Energy and Macronutrients Intake

Twenty-five of the studies assessed the energy and macronutrient intake of various samples of BP. The following methods were utilised for dietary intake evaluation: 3- [15,20,21,22,24,27,29,37,38], 4- [26], 5- [30,31], or 7-day [34,35] food diaries (FD); 24 h [16,19,77], 3 × 24 h [23,25], or 7 × 24 h [17] dietary recall (DR); FFQ [18,28,33], or 4-day recording via mobile app [39]. One study utilised a doubly labeled water method for the evaluation of energy expenditure [36]. 

For the proper understanding of the subsequent parts of the text, it needs to be underlined that the results/values preceded with ‘~’ in the subsequent parts of the manuscript refer to the results/values calculated by the authors of the current review based on original data presented in corresponding papers. In Table 1, these data are marked with ‘^ǂ^’ and are written in italics. Moreover, the data taken directly from the referred papers are provided in the text and the tables (Table 1, Table 2, Table 3 and Table 4) with original writing of decimal places.

Eight of the included studies (32% within the discussed area) investigated energy and macronutrients’ intake in BP aged <18 years [15,16,18,24,28,31,34,35] (Table 1). The estimated energy value of habitual diet varied from 2895 [35] to 3962 kcal∙day^−1^ [28] [from ~35.8 [35] to 51.1 kcal∙kg_BM_^−1^∙day^−1^ [28]] in male and from 1801 [34] to 2854.5 kcal∙day^−1^ [from ~27.5 [34] to 41.6 kcal∙kg_BM_^−1^∙day^−1^ [16]] in female young BP, respectively. In the study by Dzimbova [18] on BP aged 15.4 years (with no indication of males and females ratio), the estimated energy intake (EI) was 2204 kcal∙day^−1^ [~32.2 kcal∙kg_BM_^−1^∙day^−1^]. PRO intake ranged from 135.4 [35] to 150 g_PRO_∙day^−1^ [34] (from ~1.7 [35] to ~1.9 g_PRO_∙kg_BM_^−1^∙day^−1^ [34]) in males and from 82 to 104 g_PRO_∙day^−1^ [from ~1.2 to ~1.6 g_PRO_∙kg_BM_^−1^∙day^−1^] in females depending on the period of the season (with lower values during competitive season [34]). CHO intake ranged from 365.5 [35] to 487.8 g_CHO_∙day^−1^ [28] [from ~4.5 [35] to 6.3 g_CHO_∙kg_BM_^−1^∙day^−1^ [28]] in males and from 218.8 [35] to ~375 g_CHO_∙day^−1^ [16] (from ~3.4 [35] to 5.4 g_CHO_∙kg_BM_^−1^∙day^−1^ [16]) in females. While FAT intake ranged from 93.5 [35] to 165.6 g_FAT_∙day^−1^ [28] (from ~1.2 [35] to ~2.2 g_FAT_∙kg_BM_^−1^∙day^−1^ [28]) in males and from 63 [34] to ~113 g_FAT_∙day^−1^ [15] (from ~0.96 [34]) to 1.6 g_FAT_∙kg_BM_^−1^∙day^−1^ [15]] in females.

Regarding the adult male and female able-bodied BP, eleven of included studies (44%) reported on habitual energy and macronutrient intake [16,21,23,25,26,27,29,30,32,39,93]. The estimated EI of male BP varies considerably between studied groups and ranged from 1901 [30] to 4521.1 kcal∙day^−1^ [16] (from 21 [30] to 52.9 kcal∙kg_BM_^−1^∙day^−1^ [16]). The estimated EI noted by Papandreou et al. [30] was extremely low (and probably underestimated) and when expressed in relation to BM was even lower in males compared to females (25 kcal∙kg_BM_^−1^∙day^−1^). In both genders, it was lower compared to estimated energy expenditures (EE). Simultaneously, the energy value noted by Papandreou et al. [30] in females was the lowest (1487 kcal∙day^−1^) among other included studies. The highest absolute EI in female BP was reported by Nepocatych et al., at 2567 kcal∙day^−1^ (34 kcal∙kg_BM_^−1^∙day^−1^ [27]), and the highest relative value by Leinus and Ööpik [26] was ~34.6 kcal∙kg_BM_^−1^∙day^−1^ (2185 kcal∙day^−1^). Estimated PRO intake ranged from 79.3 [21] to 211.3 g_PRO_∙day^−1^ [32] [from relative values as low as about 1.0–1.1 [26,30] to 2.3 g_PRO_∙kg_BM_^−1^∙day^−1^ [32]] in males or from ~50 [26] to 97.9 g_PRO_∙day^−1^ [39] (from 0.8 [26] to 1.31 g_PRO_∙kg_BM_^−1^∙day^−1^ [39]) in females. Estimated daily CHO intake ranged from 220 [30] to ~514 g_CHO_∙day^−1^ [16] (from 1.9 [30] to 6.0 g_CHO_∙kg_BM_^−1^∙day^−1^ [16]) in males and from 170 [30] to 304 g_CHO_∙day^−1^ [27] (from 2.9 [30] to 4.1 g_CHO_∙kg_BM_^−1^∙day^−1^ [27]) in females. The intake of FAT ranged from 58.5 [21] to 185.3 g_FAT_∙day^−1^ [32] (from ~1.7 [29] to 2.1 g_FAT_∙kg_BM_^−1^∙day^−1^ [32]) in males and from 63 [29] to 113 g_FAT_∙day^−1^ [39] (from ~0.9 [29] to 1.5 g_FAT_∙kg_BM_^−1^∙day^−1^ [39]) in females.

Seven studies (28%) investigated energy and nutritional value of habitual diet in para-athlete BP [17,19,20,22,33,37,38], of which six studies focused on wheelchair BP [19,20,22,33,37,38] and one on deaf BP [17]. Among female wheelchair BP, EI ranged from ~1635 [33,38] to 2867.8 kcal∙day^−1^ [19] (from 26.8 [38] to ~50.0 kcal∙kg_BM_^−1^∙day^−1^ [19]), while in male adult wheelchair BP it was comparable across studied groups [20,22,37,38], being ~2400–2500 kcal∙day^−1^ [from 32.4 [37] to 34.8 kcal∙kg_BM_^−1^∙day^−1^ [20]]. CHO intake among female wheelchair BP ranged from ~226 [37] to 297.3 g_CHO_∙day^−1^ [19] (from ~3.2 [19] to 3.7 g_CHO_∙kg_BM_^−1^∙day^−1^ [37]). In males CHO intake oscillated from ~233 [22] to ~318 g_CHO_∙day^−1^ [20] (from 3.1 to 4.24 g_CHO_∙kg_BM_^−1^∙day^−1^). PRO and especially FAT intake contribution differentiated EI of female wheelchair BP to a great extent. PRO intake ranged from 57.5 [33] to 92.6 g_PRO_∙day^−1^ [19], which corresponded to 1.0 [37] and ~1.6 g_PRO_∙kg_BM_^−1^∙day^−1^ [19], and FAT intake ranged from ~55 [37] to 142.7 g_FAT_∙day^−1^ [19] (from 0.9 to ~2.5 g_FAT_∙kg_BM_^−1^∙day^−1^ [19,37]). In adult male para-athlete BP, PRO intake across studies groups ranged from ~111 [20,37,38] to ~126 g_PRO_∙day^−1^ [20] (from~1.5 [20,37,38] to ~1.6 _PRO_∙kg_BM_∙day^−1^ [20,22]], while FAT intake ranged from ~92 to 104 g_FAT_∙day^−1^ [20] (from 1.23 to 1.39 g_FAT_∙kg_BM_^−1^∙day^−1^ [20]). In the study by Toti et al. [37], the group of male wheelchair BP who did not receive dietary advice (NDAM-T3; *n* = 12) consisted of 47% of senior BP and 53% of junior BP. Thus, the results regarding estimated energy and macronutrients intake in this particular study deviated from the results observed in the remaining groups of adult male wheelchair BP [20,22,38].

### 3.3. Hydration Practices and Hydration Status

A total of 13 studies evaluating habitual hydration practices and hydration status [32,40,41,42,43,44,45,46,48,49,50,51], including 1 study on wheelchair BP [47], were identified and are included in this review (Figure 1, Table 2). 

According to the most widely used and recommended by the American College of Sports Medicine [94] cut-off point for euhydration (USG < 1.020), the mean pre-game/pre-practice USG indicating a proper hydration status was noted in six of the included studies [40,44,46,47,48,49]; however, in two of these studies [46,86], solely one out of a few performed evaluation occasions. Eight studies [40,41,46,47,48,49,50,51] provided the direct numbers of athletes being dehydrated (DEH) before the beginning of the training unit and/or competition (however, using various indicators and classifications for defying the state of dehydration). In able-bodied BP, the number of DEH athletes ranged from 40 [40] to 75–95% (depending on the indicator of hydration status taken into consideration [51]), or even 100% in the study by Thigpen et al. [50]. In wheelchair BP, 1 (9%) out of 11 athletes was DEH before the training. Interestingly, Heishman et al. [46] revealed greater pre-exercise mean USG and a greater number of DEH cases during competitive compared to pre-season evaluations.

The mean percentage of BM loss during training/games ranged between 0.6 [40,44] and 2.9% [50] in able-bodied BP and 0.4 and 0.6% in wheelchair BP [47]. Mean in-training/in-match BM loss referring to ‘well hydration’ (+1 to −1% change in BM [95]) was observed in two studies on able-bodied BP [40,44] and in the study on wheelchair BP [47], while referring to minimal dehydration (−1 to −3% change in BM) in three other studies [41,48,50]. Still, it needs to be taken into consideration that in the case of pre-exercise DEH existence, even the percentage of BM loss that refers to ‘well hydration’ must be considered as a deviation, while it may escalate the baseline (pre-exercise) DEH. An example can be found in the paper by Arnaoutis et al. [41], in which mean BM loss during exercise was −1.0 ± 0.01% but the incidence of pre-exercise EUH was solely 16.7% (83.3% of participants were DEH already before exercise). Abbasi et al. [40] revealed the level of fluid replacement equal to 59.4 ± 27.3% in female BP, while in the study by Brandenburg and Gaetz [44], fluid intake in relation to sweat loss during the game was ~78%. Other studies also pointed out that the fluid intake/fluid intake rate during basketball practice/competition was lower compared to sweat loss/sweating rate [45,48,50,51].

### 3.4. Dietary Interventions on Dehydration and Hydration Strategies

A total of nine studies reporting on interventions related to the impact of DEH and/or various hydration strategies on performance and performance-determining factors were included in this review [42,49,52,53,54,55,56,57,58]. A total sample size of all studies consisted of 117 BP (100 males and 17 females), whereas wheelchair BP were not examined in this area (Table 3). Among included studies, DEH was executed either via heat/exercise procedures to achieve the preconceived/targeted degree of DEH [42,52,54,57] or via refraining from fluid intake during exercise [49,53,56]. Most of the aforementioned studies compared DEH condition(s) with EUH obtained with the use of various fluids [42,52,53,54,56]. Moreover, two studies [49,58] implemented protocols relying on the comparison of various hydration strategies’ effectiveness, with no comparison to DEH state.

The two most comprehensive investigations on the impact of different levels of DEH (1–4% DEH) on a wide range of physiological and cognitive- or discipline-specific performance outcomes were performed by Baker and colleagues [42,52]. Similar procedures (EXERCISE/HEAT exposure) in evoking targeted DEH (solely 2% DEH investigated) was applied and similar outcomes were evaluated by Dougherty et al. [54]. All the mentioned studies indicated that DEH was related to greater core temperature (T_c_) compared to EUH state, especially at the time point right after EXERCISE/HEAT exposure and especially when DEH level was ≥2%. Even 1% DEH was related with substantial reduction in plasma volume (PV) [42,52]. The impact of DEH (1–4%) on elevation in HR was particularly noticeable right after the EXERCISE/HEAT exposure [42,54], and the recovery in this respect was retarded when 4% DEH was evoked [42]. Mean arterial pressure (MAP) was significantly impaired at 3–4% DEH [42] but not 1–2% [42,54]. Moreover, the study by Baker et al. [52] revealed that attentional vigilance was significantly impaired by DEH (in relation to most of its studied aspects). The impairment was noticeable to a minor degree right after EXERCISE/HEAT exposure, but the accumulation of adverse effects of DEH was particularly noticeable at the END of the whole test procedure. While in the study by Hoffmann et al. [56], ~2.3% DEH (resulting from refraining from fluid intake) led to the impairment in lower-body reaction time (regardless of the hydration condition used as a comparator), as well as visual and physical reaction time (compared to water enriched with 1 g per 500 mL of L-alanyl-L-glutamine) but had no effect on motor reaction time. In general, the subjective ratings of various aspects of fatigue (i.e., lightheadedness, windedness, hotness, muscle cramping, side stitch/ache, or upper-, lower- and total body fatigue) were elevated due to DEH in a varying degree [42,52,54]. However, the most prominent elevations in the indicators of fatigue were noted when 3–4% DEH was evoked [42].

The comprehensive evaluation of the relationship between the level of DEH and discipline-specific (basketball) performance by Baker et al. [42] indicated 2% DEH as a threshold at which performance decrement reached statistical significance (at least for selected performance indices). This seems to be in line with the study by Dougherty et al. [54], in which the targeted 2% DEH led to substantial decrements in most of the indices in the basketball drill test, but contrary to the investigation by Louis et al. [57], where a targeted 2% DEH was tolerable concerning maintaining performance and technique in three-point shots tests in elite BP. In the studies by Hoffmann, Stavsky, and Folk [55] and Carvalho et al. [53] DEH equal to ~−1.9 and −2.46% BM loss, respectively (resulting from prohibition in fluid intake), did not affect basketball performance, while Hoffmann et al. [56], in their more recent study, revealed DEH equal to ~−2.3% BM loss substantially decreased performance in field goal shooting (compared to intake of water enriched with 1 g per 500 mL of l-alanyl-l-glutamine).

Five of the included studies [42,49,53,54,57] utilised widely used and validated Borg’s Rating of Perceived Exertion Scale (RPE). Within two protocols that implemented targeted procedures of DEH [42,54], RPE was elevated right after exposure to EXERCISE/HEAT but not after completion of the whole test protocol. The phenomenon seemed to be more pronounced when 3–4% DEH was evoked (unlike 1–2% DEH; [42]). In the study by Louis et al. [57], the targeted 2% DEH resulted in increased RPE after exercise tests. Unlike studies by Baker et al. [42] and Dougherty et al. [54], the latter of the mentioned protocol did not consider recovery between DEH procedures and exercise tests [57]. In the study by Carvalho et al. [53] 2.46% BM loss (as a consequence of a forced by the study protocol refrain from fluid intake during exercise) led to increased RPE compared to ad libitum water (−1.08% BM loss) or carbohydrate-electrolyte solution intake (CES; −0.65% BM loss). Finally, Taim et al. [49] did not observe differences in RPE when ingesting plain- (~−0.941% BM loss) or flavoured water (~0.534% BM loss) during exercise. Furthermore, the two last-mentioned studies compared the effectiveness of various fluids on replenishing fluid loss during exercise and cognitive- or discipline-specific performance outcomes; however, the results are still inconclusive. Both studies by Baker and colleagues [42,52], despite investigating EUH obtained by the means of two different fluids, (1) lemon/lime-flavored CES (6% CHO; 18.0 mmoL∙L^−1^ Na; EUH-CES) or (2) lemon/lime-flavored water (18.0 mmoL∙L^−1^ Na; EUH-PLA), did not present results obtained by the implementation of those two fluids separately. This was due to a lack of significant differences in measured outcomes between these two EUH conditions. In contrast, the implementation of the same fluids by Dougherty et al. [54] revealed some advantages of EUH-CES over EUH-PLA concerning selected aspects of performance in basketball drill tests (i.e., combined shooting or suicide sprinting). Carvalho et al. [53] found no differences in performance outcomes when ad libitum ingestion of water (3.8 mg∙L^−1^ Na) or CES (7.2% sugar, 0.8% maltodextrin, 510 mg∙L^−1^ Na) was implemented. Hoffman et al. [56] found no differences in fluid intake or outcomes of reaction, performance, and power when ingesting water combined with 1 g or 2 g of L-alanyl-L-glutamine per 500 mL. In the study by Minehan et al. [58], CES (6.8% CHO, 1130 kJ∙L^−1^, 18.7 mmol∙L^−1^ Na, 2 mmol∙L^−1^ K) and low-energy-electrolyte beverage (1% CHO, 170 kJ∙L^−1^, 18.7 mmol∙L^−1^ Na, 3 mmol∙L^−1^ K) vs. water in females and CES vs. water in males were more effective in assuring proper fluid balance. Finally, Taim et al. [49] revealed greater palatability ratings for flavoured compared to plain water but without further effects on the remaining evaluated outcomes. 

### 3.5. Dietary Interventions on Macronutrients’ Manipulations

A total of 12 studies on dietary interventions related to CHO, PRO, and FAT manipulations were included in the systematic review [59,60,61,62,63,64,65,66,67,68,69,70] (Table 4), of which one was the study in wheelchair BP [64]. Four studies [59,60,61,63] were single acute evaluations of macronutrients manipulations, concerning CHO [59,60], PRO [63], or CHO/PRO [61] quantity and/or quality. Four studies were short- (10 days) [68] to moderate-term (30 days/4 weeks) duration of PRO [67] or CHO [65,66] supplementation. The next two studies were long-term (8 weeks) evaluations on PRO [69,70] alternations. Eventually, the remaining studies were 30-day [64] or 6-week [62] supplementation protocols with polyunsaturated fatty acids (PUFA).

Afman et al. [59] reported that a single acute ingestion of 75 g CHO (sucrose; CHO-SOL) 45 min before exercise impaired 20 m sprint time in a 1st QR of basketball stimulated test and improved it in the 4th QR compared to placebo (PLA), whereas the 1st QR and overall mean layup shooting was significantly lower after CHO-SOL consumption. Moreover, ingestion of CHO resulted in an increased pre-exercise glucose concentration that was lower at 1st QR. In the study by Shi [68], 10 days of intake of a 500 mL beverage containing 100 g_CHO_∙L^−1^ was associated with a higher reduction in blood urea nitrogen concentration and creatine kinase (CK) activity between pre- and post-exercise evaluations compared to PLA. Blood glucose concentration after supplementation significantly increased right after post-exercise compared to PLA but did not differ at pre-exercise or 30 min post-exercise evaluations. In none of the two mentioned studies [59,68] did blood lactate concentration change in dietary intervention groups compared to PLA. Daniel et al. [60] reported that ingestion of high glycemic index (HGI) dinner and evening snacks as an individual case the day before competition was associated with a higher intake of CHO compared to their consumption at a corresponding meal consisting of low glycemic index (LGI) products/dishes. There were no differences between the consumption of HGI vs. LGI evening meals in variables of sleepiness or concentrations of cortisol and melatonin in saliva. At HGI, the glycemic response was higher; however, satiety indices did not differ between conditions. Eventually, Michalczyk and colleagues [65,66] investigated the effect of 4 weeks of a low carbohydrate diet (LCD; 10% CHO, 31% PRO, and 59% FAT in total energy intake, TEI) followed by 7 days of CHO loading (Carbo-L, 75% CHO, 16% PRO, 9% FAT in TEI) on body composition [65,66], blood markers of lipid and CHO metabolism [65], acid-base balance indices [66], and hormones’ concentration [66] or anaerobic performance [66] in male adult Polish BP. One month before the experiment, athletes in both studies consumed a conventional diet (CD) that provided 54% CHO, 15% PRO, and 31% FAT in TEI. Interestingly, there were inconsistencies between the two discussed studies in the outcomes related to BM and body composition (see Table 4 for details). Triglycerides (TG) concentration was lower after LCD compared to CD, and Carbo-L led to an increase in TG and glucose concentrations compared to values noted when consuming CD. No changes were observed concerning concentrations of total-, HDL-, LDL-cholesterol and insulin or HOMA-IR [65]. LCD led to a decrease in blood lactate concentration and pH and an increase in β-hydroxybutyrate concentrations at rest compared to CD, yet Carbo-L resulted in returning to the baseline values (no differences between Carbo-L and CD) [66]. No differences were found in the aforementioned variables at post-exercise evaluations. In addition, testosterone concentration increased after LCD and remained increased after Carbo-L compared to CD [66]. There was a substantial reduction in insulin concentration after LCD, which, however, returned to the baseline value after Carbo-L. Simultaneously, there was a substantial increase in the concentration of growth hormone after LCD and a return to the baseline value after Carbo-L [66]. Finally, LCD led to a decrease in total work during the 30 s Wingate Anaerobic Test, which, however, recovered to baseline values after Carbo-L. There were no changes in peak power (PP) or time to PP [66].

In the study by Gentle et al. [61], pre-exercise (90 min) ingestion of 1 g_CHO_∙kg_BM_^−1^ in conjunction with 1 g_PRO_∙kg_BM_^−1^ (CHO-PRO) compared to consumption of 2 g_CHO_∙kg_BM_^−1^ alone resulted in higher blood glucose concentration during and post-exercise (exercise lasting 87 min) and a lower increase in post-exercise CK activity and cortisol concentration. Furthermore, HR, blood lactate, or testosterone levels did not differ between study conditions at any time point. Regarding basketball-specific performance, the CHO-PRO condition gave an advantage over sole consumption of CHO in mean success rate for the first two free-throw attempts at the 4th QR. No differences were found in jump height or sprint time. The conditions did not differ in preventing muscle soreness. However, CHO + PRO contributed to greater upset of gastrointestinal side effects and greater RPE at the 1st and 4th QRs [61].

Ho et al. [63] found that a single oral ingestion of 600 mL of high-PRO compared to a low-CHO drink after 1 h of endurance cycling enhanced recovery and resulted in longer time to exhaustion (~16%) in a subsequent (after 2 h) cycling time trial [63]. Still, it needs to be underlined that exercise tests implemented by Ho and colleagues [63] do not reflect the type of exercise efforts undertaken during basketball matches. Thus, the results of the study may have limited utility in relation to basketball. The study by Ronghui [67] revealed that 30 days of supplementation with 20 g of whey PRO (W-PRO) plus 40 g of oligosaccharides dissolved in 250 mL of milk compared to ingestion of 250 mL milk only resulted in improvement in selected blood haematological markers (such as haemoglobin, red blood cell count, haematocrit, and mean corpuscular volume). Eventually, Taylor et al. [69] and Wilborn et al. [70] performed long-term (8 weeks) evaluations of the effectiveness of PRO supplementation in BP, of which the first mentioned [69] compared ingestion of W-PRO (2 × 24 g_W-PRO_∙day^−1^) vs. maltodextrin, while the second compared W-PRO (2 × 24 g_W-PRO_∙day^−1^) vs. casein PRO (C-PRO; 2 × 24 g_C-PRO_∙day^−1^). Within both protocols, ingestion of W-PRO resulted in favourable changes in body composition (increase in lean body mass and decrease in fat mass). W-PRO compared to maltodextrin ingestion resulted in an improvement in agility drill time [69]; however, there were no advantages of W-PRO compared to C-PRO in strength outcomes or agility drill time [70].

Data on dietary interventions related to FAT alternations in BP are very limited. Previously mentioned investigations by Michalczyk and colleagues [65,66] employed LCD dietary protocols, in which the contribution of energy from FAT was ~59% of TEI (compared to 31% of TEI in CD). Still, the contribution of FAT was not high enough to classify the diet as ‘ketogenic’ or to evoke the state of ketosis, and greater emphasis was paid to the lower availability of CHO than increased FAT contribution. Apart from the discussed investigations, solely studies by Ghiasvand et al. [62] and Marques et al. [64] brought up the issue of the effectiveness of lipid constituents’ supplementation in BP. Ghiasvand et al. [62] employed supplementation with eicosapentaenoic acid (EPA) alone or in conjunction with vitamin E for 6 weeks, while Marques et al. [64] supplemented with 3 g of fish oil (1500 mg docosahexanoic acid [DHA], 300 mg EPA, and 6 mg vitamin E) for 30 days in wheelchair BP. Solely the supplementation with EPA + vitamin E (and not with EPA alone) was effective in improving selected indices of the inflammatory and antioxidant status of the body [62]. In turn, the most important practical observations by Marques et al. [64] were the effectiveness of implemented supplementation concerning prevention of increases in the plasma activity of lactate dehydrogenase and concentration of IL-6, the loss of membrane integrity, as well as favourable alterations in exercise-induced reactive oxygen species.

### 3.6. Eating Behaviours and Nutritional Knowledge

A total of 24 studies (20 OS and 4 IS) assessing eating behaviours or NK were included in this review. The total sample size of those studies was 1277, of which 102 athletes were wheelchair BP. According to the scope of the studies, they were divided into the following categories: meal frequency, breakfast consumption, timing of meal consumption, frequency of food groups consumption, types of food consumed before training/competition, hydration habits, drinking alcohol and smoking, disordered eating (DE) and other psychological aspects, NK, and eventually dietary counseling interventions.

#### 3.6.1. Frequency and Timing of Meals Consumption, and Breakfast Consumption Habits

The gathered data revealed that meal frequency differed considerably between various groups of BP [20,39,79,89], as well as between training season time points [85]. About 63.6% of Polish male and female professional BP declared consuming 4–5 meals∙day^−1^ and 45.8% of the regular timing of meals consumption (every 3 h) [89]. Zanders et al. [39] found variation in the number of meals∙day^−1^ in NCAA Division II female BP across the entire season (3.3–4.2 meals∙day^−1^), with the highest number observed during the in-season phase (characterized by heavy practicing and participation in conference league games). Mavra et al. [85] noted a substantial differentiation in frequency of meal consumption/skipping according to division, with female Croatian BP from the 1st division declaring consuming 3 meals∙day^−1^ more often and skipping meals less frequently, compared to athletes from the 2nd division. Sánchez-Díaz et al. [79] in Spanish BP under 14 years (U14) revealed that the frequency of meal consumption differs between genders, with 84.6% of boys and 70% of girls declaring consumption of 3 meals∙day^−1^ on a regular basis. The frequency of meal consumption in Spanish male wheelchair BP in the pre-competitive period ranged between 3.8 ± 0.8 (May) and 4.0 ± 0.8 (June) meals∙day^−1^. 

Apart from a total number of meals consumed, the particular importance of breakfast consumption concerning basketball performance has been raised in the cross-over IS by Čabarkapa et al. [92]. The athletic performance in free-throw shooting in male BP was higher at breakfast consumption days compared to no-breakfast consumption days. Still, the significance level in this study was set at *p* < 0.10 and no relationships were observed in 2- or 3-point attempts. Sánchez-Díaz et al. [79] noted that all females and 92.3% of males U14 BP declared eating breakfast on a regular basis. In the study by Mavra et al. [85], the percentage of regular breakfast consumers (5–7 times∙week^−1^) was 64.56 among the 1st and 60.81% among the 2nd division BP. Musaiger and Ragheb [88] noted daily consumption of breakfast solely in 46.2% of studied Bahraini BP. Still, the data came from 1994. 

Timing and composition of pre- and post-exercise meals have been investigated in a few studies [19,32,88,89], with mean time of the last meal before competition ranging from 192 ± 55 min (~3.2 ± 0.92 h) in Spanish male BP [32] to 204 min (3.4 h) in Bahraini BP [88], and with 19.6% of Polish male and female BP declaring consumption of the last meal 2–3 h before training. Pre-exercise meals most frequently consisted of rice (82.1% of participants), vegetable salads (51.3%), and meat (30.8%) in Bahraini BP [88] and spaghetti (90%), meat other than chicken (86.3%), and salads (37.8%) in Spanish BP [32]. The post-exercise meal was reported to be consumed 120 ± 45 min (~2 h) after competition in Spanish BP [32], and 48.6% of Polish athletes declared consuming meals 1–2 h after training [89]. Eskici and Ersoy [19] noted that pre-training meals in female Turkey wheelchair BP included water (77.3% athletes) and fruits (54.5%), in-training meals consisted of water (90.9%) and candy or chocolate (27.3%), and post-training meals consisted of water (95.5%) and fruits (40.9%). In the study by del Mar Bibiloni [81], overall hydration habits of amateur Spanish male and female BP were rated as ‘good’ in 54.6, 74.2, and 76.5% of athletes before, during, and after training, respectively. However, as many as 20.8 and 17.5% of BP reported not consuming fluids before training and competitions, and it was more pronounced in females (27.6 and 25.3%) compared to males (14.6 and 10.4%), while lack of proper hydration during training was more prevalent in males (9.4%) compared to females (1.2%). Drinking habits seemed to be more appropriate during competition days compared to training days, and the most preferred fluid was water [81]. Among Spanish BP [32], 44% of studied athletes demonstrated a lack of the proper habit of consuming fluids before exhibiting thirst, and no fluid consumption during training and competition was reported by 3 and 2 (out of 50) athletes, respectively. The most highly consumed fluids were water (~54% share of total daily fluid intake), followed by milk (~26%), commercial sports drinks (~12%), or carbonated beverages (~10%) [32]. Gender-dependent tendencies in hydration habits were also observed in elite young BP players [79], with 69.2 of males and 40% of females declaring drinking at least 1–1.5 L of water every day. Among Polish adult BP [89] 69.2% of BP replenished fluids with mineral water and 39.3% consumed more than 2.5 L of water per day.

#### 3.6.2. Food Groups’ Contribution to Daily Food Rations and Composition of Pre- and Post-Exercise Meals

A few studies have reported on the frequency of food groups consumption or their contribution to daily food rations in BP [79,89]. Among Spanish U14 BP [79], the percentage of players who declared consumption of at least 200 g of fruits every day on a regular basis (‘always’) was 46.2 in males and 20% in females; ‘often’ consumption was reported by 30.8 and 30% of participants, respectively. As many as 20% females declared that they ‘never’ consume 200 g of fruits per day. Even worse habits were noticed concerning vegetable intake. As many as 15.4, 30.8, 23.1, and 23.1% of males answered that they consume at least 200 g of vegetables every day as frequently as ‘never’, ‘sometimes’, ‘often’, and ‘always’, respectively. The corresponding values in females were 0, 70, 30, and 0%, respectively. Szczepańska and Spałkowska [89] in a sample of Polish male and female adult BP found that the percentage of athletes presenting proper eating habits concerning frequency of consumption of foods perceived as having high nutritional value and ’pro-health’ effect was 82.3% for meat and meat products (proper eating behaviour perceived as consumption as frequent as every day), 77.6% for milk and fermented milk products (every day), 73.8% for eggs (few times a week), 71.1% for fruits (few times a day), 43.0% for wholemeal bakery products and groats (every day), 41.1% for fresh/cottage cheese (few times a week), 30.8% for vegetables (few times a day), and 29.9% for fish (few times a week). Concerning foods possessing poor nutritional value/adverse impacts on health it was 39.2% for ‘fast foods’ (occasionally or never) and 11.2% for cheese/blue cheese (few times a month). Other proper eating behaviours, namely, eating raw (not processed) fruits were reported by 77.6% of participants, choosing low-fat poultry meat by 77.6%, eating raw (not processed) vegetables by 71.0%, and frying without fat by 8.4%. According to the data by Davis et al. [93], the habitual diet of BP seems to be characterized by low fish consumption. Dietary intake of athletes of the National Basketball Association (NBA) showed that 31% of players reported consuming no fish in their diet per week, with 61% of players reported consuming less than 2 servings of fish per week.

#### 3.6.3. Alcohol Use and Smoking Habits

The literature gathered via the current systematic review identified some adverse eating and lifestyle behaviours related to the use of alcohol [19,28,29,79] or smoking habits [19,38]. The phenomenon is the most alarming in young athletes, aged < 18 years. Sánchez-Díaz et al. [79] noted that among U14 Spanish BP, the percentage of boys drinking wine or beer at meals ‘on a regular basis’ was 15.4% and of those who drank alcohol ‘sometimes’ was 23.1%. Corresponding percentages in their female counterparts were 0 and 40%, respectively. While the study by Nikić et al. [28] revealed the consumption of alcohol was between 0.4 and 0.8 g∙day^−1^ (0.1–0.2% of TEI) in Serbian male junior elite BP. Among adult college BP, the consumption of alcohol was 3.6 ± 7.8 in males and 2.0 ± 6.3 g∙day^−1^ in females [29]. Two of the included studies recorded alcohol intake and smoking cigarettes in wheelchair BP [19,38]. None of the athletes studied by Eskici and Ersoy [19] reported alcohol consumption, but 22.7% of them declared smoking cigarettes, while in the study by Toti et al. [38], 2 out of 15 athletes smoked occasionally or on a regular basis, and 13 athletes drank alcohol, with alcohol intake ranging between 4.1 ± 1.2 g∙day^−1^ (0.7 ± 0.2% of TEI). None of the studies reporting on alcohol intake (g∙day^−1^ or % of TEI) indicated the type of alcohol consumed or provided information on whether the given values correspond to alcoholic beverages or the intake of pure ethanol (C_2_H_5_OH).

#### 3.6.4. Disordered Eating Behaviours

In general, female BP [84,86,87,90] were more often investigated concerning disordered eating (DE) behaviours compared to males [83]. The study by Michou and Costarelli [86] revealed that 11% of studied female BP demonstrated DE attitudes based on the Eating Attitudes Test-26 (EAT-26), which, in fact, was lower compared to their non-athletic peers (15%). Wells et al. [90], based on the ATHLETE survey, found that female varsity BP, along with athletes representing sports disciplines such as softball, soccer, or golf, displayed a lower prevalence of psychological factors and behaviours associated with DE compared to female athletes practicing swimming, volleyball, or cross country running. Similarly, Kampouri et al. [84] found that, in general, elite Greece team sport players (basketball, water polo, volleyball) exhibit a similar prevalence of DE behaviours (5.1%) compared to their non-athletic peers (1.1%) based on the Eating Disordered Examination Questionnaire (EDE-Q). Solely the ‘eating concern’ subscale of EDE-Q differed between studied team sports, with water polo athletes exhibiting higher values compared to basketball or volleyball players (VB). No differences were found between BP and VB. Monthuy-Blanc et al. [87] found no differences between adolescent French female BP, ballet dancers, and their non-athletic peers in any of the disturbed eating and behaviours investigated via the Eating Disorders Inventory. Regarding male BP, Gorrell et al. [83] found mean EDE-Q score equal to 0.63 and percentage of athletes with clinical global EDE-Q about 12% (2 out of 16 studied BP; percentage lower compared to baseball, cycling, or volleyball and higher compared to triathlon, ice hockey, wrestling, football, rowing, gymnastics, fencing, running, soccer, swimming, cheerleading, lacrosse, ultimate frisbee, or water polo), and with clinical eating, weight, and shape concerns each equal to about 6% (1 out of 16 studied BP). Interestingly, the rate of athletes exhibiting binge eating behaviours was the highest among BP (50%, *n* = 8) compared to other investigated sports disciplines [83]. Only one of the included studies examined DE behaviours among wheelchair BP (males), and it found no differences in the risk of *orthorexia nervosa* (based on the ORTO-15 questionnaire) compared to gym attendees or inactive individuals [38]. Simultaneously, lower ORTO-15 scores (lower risk of *orthorexia nervosa*) were linked with higher adherence to the Mediterranean dietary pattern, higher share of FAT and lower share of CHO in TEI, higher gastro esophageal reflux disease symptoms and lower starvation symptoms inventory (lower risk of eating disorders, e.g., *anorexia nervosa*). Thus, medical conditions typical to wheelchair individuals may substantially impact their food choices and eating behaviours. Moreover, psychological factors need to be taken into account when analyzing the determinants of basketball players’ eating behaviours and nutritional choices. Gacek [21] found a positive relationship between the level of self-efficacy (as evaluated via the Generalised Self-Efficacy Scale, GSES) and the quality of diet (energy value, water intake, PRO, CHO, sucrose, polyunsaturated fatty acids content, intake of certain vitamins, i.e., A, E, B_1_, B_3_, B_6_, C, and minerals, i.e., Na, K, Ca, Mg, P, Fe, Cu, J) in Polish adult male BP. The other study on Polish BP [82] revealed positive relationships between a sense of internal and localized health control, or level of self-efficacy (GSES), and more rational food choices.

#### 3.6.5. Nutritional Knowledge and Dietary Counseling Interventions

The literature included in the current systematic review indicated poor/incorrect NK in BP [19,78,79,80]. Each of the four identified studies utilised different tools to evaluate NK, of which solely one questionnaire was a validated tool [78] and the three remaining tools [19,79,80] were developed based on previously used tools; however, their validity remains unclear. Boumosleh et al. [80] found that 80% of male and female Division I Lebanon BP, and 54% of coaches had inadequate NK. Similarly, Escribano-Ott et al. [78] noticed insufficient and inadequate NK in the sample of Spanish male and female U18 BP, as well as adult professional and non-professional players. The total score of NK ranged between 4.28 and 4.6 (out of 10 points). From the five evaluated thematic blocks—‘hydration’, ‘weight management’, ‘recovery’, ‘nutrients’, and ‘supplementation’—the lowest score was noted for the ‘supplementation’ block. The significant difference between subgroups was found within the ‘nutrient’ block, with non-professional players exhibiting the highest score. Sánchez-Díaz et al. [79] in male and female Spanish U14 BP found relatively poor NK, with less than 50% of questions being answered correctly. No gender-related differentiation was noticed. The NK of wheelchair BP seems to be insufficient likewise [19], with rather better knowledge of basic nutrition compared to sport-specific NK. 

Some authors indicated a lack of nutritional education [80] or lack of professional support or time management difficulties [78] as a reasons for poor sport-specific nutritional knowledge. In addition, the source of NK seems to be crucial in developing proper eating habits and nutritional behaviours. Among Bahraini BP [88], the most common source of nutrition information were mass media (including television, radio, and magazines; 48.7% of participants); about one-third of participants indicated that they did not have any source of NK, while 15.4 and 5.2% indicated other players and coaches as a source of NK. Nevertheless, the data came from 1994. Eskici and Ersoy [19] reported that for as many as 40.9, 40.9, 31.8, and 18.2% of wheelchair BP, their sources of NK were trainers, mass media, books on nutrition, and dieticians, respectively. Moreover, dietitians/nutritionists (89.9%), strength and conditioning coaches (66.7%), and college nutrition/health courses (65.7%) were the three main resources to obtain current NK by Lebanon basketball players [80]. Gender influenced the likelihood of using particular sources of NK. Specifically, females compared to males are more unlikely to use coaches and assistant coaches but more likely to use strength and conditioning coaches, magazines, friends, parents, and the Internet as a source of NK. Despite widespread insufficient NK, Boumosleh et al. [80] reported that Lebanon sports clubs neither have dietitians nor carry out nutrition education campaigns. 

Apart from Boumosleh et al. [80], the necessity of nutrition-focused education interventions was noticed also by other authors [19,78,79], and a few studies examined this aspect in BP [22,37,91]. In the study by Tsoufi et al. [91], an elite Greek team of adult male BP was provided with nutritional guidance by a certified sports dietitian. Each athlete received personalized nutritional evaluation and counseling, including face-to-face dietary reviews and individualized weekly diet programs to prepare at home, as well as nutritional advice on food selection during trips and hotel stays. The dietary counseling led to obtaining an adequate diet quality. Still, it was particularly seen on competition days when the team stayed in hotels where the players’ diet was closely monitored. In those days, diet quality was substantially increased, reaching almost the highest possible healthy eating index score [91]. Grams et al. [22] evaluated the effects of long-term nutritional advice in high-performance male wheelchair BP, who participated in three training camps held in the pre-competitive season in two consecutive years (training camp 1 and training camp 2 were held in two following months of the same year; training camp 3 was held one year apart). In each of the training camps, nutritional evaluation was performed, based on which players received individualized feedback to optimize diet quality (e.g., recommendations regarding daily variation and amount of food groups to be consumed, pre- and post-exercise snacks). As a result of dietary counseling, the overall adequacy of micronutrient intake increased after one year, which was attributed to higher total EI and to a more varied diet characterized by higher fruit and egg consumption. Interestingly, marginal differences in micronutrient adequacy were found within four weeks between training camps 1 and 2, suggested that changing eating habits in wheelchair BP is a longer term process [22]. Similar observations were made by Toti et al. [37] in Italian male wheelchair BP. Personalized dietary advice and an interactive course on healthy diet were provided to athletes who participated in two high-intensity training camps held in the pre-competitive period during the European championship. After one year, the follow-up was performed. Dietary counseling contributed to the reduction of energy intake from sugars and fat, adjustment of PRO intake according to individual requirements, as well as an increase in DF intake to the recommended level. Furthermore, BP who received dietary advice compared to athletes without nutritional consultations improved their intake of some micronutrients.

## 4. Discussion

The current systematic review represents a unique and comprehensive elaboration on the actual nutritional value of the habitual diet, hydration practices, and hydration status, as well as eating behaviours and nutritional knowledge in basketball, which covers a wide cross-section of diverse groups of BP, including males and females of various age categories and levels of training experience, or able-bodied and para-athletes. Moreover, it synthesizes and summarizes all of the up-to-date interventional protocols related to dietary interventions on macronutrients’ alternations and hydration strategies in basketball players. The innovative and inimitable approach, which considered observational and interventional investigations, allowed us to obtain holistic insights and to disclose critical gaps in NK and improper behaviours and habits related to nutrition and hydration, which have a reflection in poor/insufficient energy and nutritional value of customary diets of BP. This issue is noticeable in both junior and senior athletes. Furthermore, regarding interventional studies, the implemented approach of data synthetization resulted in clear, concise, but complete, juxtapositions of hitherto mentioned protocols of experimental investigations related to macronutrients intake or supplementation and hydration strategies implemented in BP. Such juxtapositions unequivocally highlight discards and deficits in former experimental investigations in BP and simultaneously indicate the most urgent and necessary directions for future studies. In fact, they serve as a kind of essential guide for practitioners and researchers interested in conducting studies on the basics of applied nutrition for basketball. From this point of view, the current systematic review stands out with a high degree of utility for both people engaged in actual/practical implications of nutrition and diet (including dietary counseling and education) in basketball players (e.g., coaches, sports nutritionists and dietetics, other members of training and medical staff, as well as players themselves) and researchers undertaking investigations within this area.

### 4.1. Energy Intake and Energy Balance

Ensuring adequate energy balance in athletes is crucial for the prevention of both relative energy deficiency in sport (RED-S) [96] and weight gain/excessive fat mass accumulation as a result of excessive energy intake in relation to actual requirements. These two phenomena have adverse effects on health and basketball performance [96,97] and seem to be particularly important in athletes still in a developmental stage. A relative high proportion of body fat has been shown to impair physical capacity and performance in efforts that are frequent in basketball, e.g., explosive actions such as changes of directions (changes of activity every 1–3 s) [2,97,98,99] or vertical jumps (~1 jumps∙min^−1^) [2,97,98,100]. A higher body fat proportion in BP may also contribute to an increased risk of overuse injuries (e.g., patellar tendinopathy) [97]. However, from the point of view of the results of the current systematic review, the more alarming problem seems to be insufficient energy intake in the habitual diet of BP. 

Regarding junior BP, solely Baranauskas et al. [15] assessed the estimated habitual EI as adequate regarding energy requirements, while the majority of authors indicated great discrepancies between EI and total energy expenditures (TEE) [15,18,34,35], with TEE being higher compared to EI. Extremely low EI, e.g., observations made by Papandreou et al. (21 ± 4 in males and 25 ± 13 kcal∙kg^−1^∙day^−1^ in females) [30], is of particular concern because it poses a high risk of the development of RED-S and its further health and performance-related complications. Low EI reported in many of the included studies may partially arise from underreporting food intake and partially reflect the actual energy deficits in BP. Interestingly, Silva et al. [34] found that in male junior BP, both TEE and EI were higher during the competitive period compared to pre-season assessment, while in females greater TEE during the competitive season were not accompanied by an increase in EI (and EI was even lower compared to pre-season measurement). Concerning adult BP, Kostopoulos et al. [25] found no differences in EI between non-match and match day. Nepocatych et al. [27] revealed considerably higher estimated EI at the end of the season compared to the beginning of the season, while Leinus and Ööpik [26] found lower EI during training days compared to resting days in both males and females, with the phenomenon being even more pronounced in males. Moreover, during training days, the estimated EI was lower compared to the estimated TEE. Meanwhile, the analysis of EE in NCAA Division II female BP performed by Moon et al. [101] revealed great differences in total and exercise EE across various types of scheduled daily activities, with EE increasing in the following order: off day, practice, conditioning, and game days. Ali Nabli et al. [102] observed that total EE during ~78 min of official game plays in male elite Tunisian U19 BP was ~504.4 kcal (the contribution of particular activities in EE was ~8.2 kcal for standing, 338.9 kcal for walking, 42.7 kcal for jogging [defined by the speed of 2.5 m∙s^−1^], 72.2 kcal for running [speed 3.2 m∙s^−1^], and 42.5 kcal for sprinting [speed 5.1 m∙s^−1^]). Interestingly, substantially greater EE were observed in 4th QR compared to 1st QR [102].

The concerns of supposed negative energy balance are prevalent in many of the other included and discussed studies [16,21,39]. Zanders et al. [39] performed a comparison of EI across the entire season. Despite minor fluctuations between particular phases of the season, no significant differences were found. However, across the entire season, the estimated TEE were higher compared to EI, resulting in a negative energy balance that ranged from −212 ± 486 to −767 ± 426 kcal∙day^−1^. In the study by Baranauskas et al. [16], the magnitude of estimated negative energy balance was ~−552 in males and ~−831 kcal∙day^−1^ in females. In Polish BP [21], the scale of estimated negative energy balance was ~−1550 kcal∙day^−1^, and only 8.33% of studied BP were categorized as meeting their energy requirements. A recent paper by Peklaj et al. [103], which is a retrospective research study based on a database of 150 athletes aged 14 to 34 years, revealed that the incidence of ‘clinical’ and ‘subclinical’ low energy availability in female young athletes was 49.2 and 32.2%, while in elite adults females it was 22.2 and 38.9%, respectively. In males the corresponding values were 31.5 and 44.4% for young and 26.3 and 47.4% for elite adult athletes, respectively. The great majority of athletes demonstrated at least one health-related symptom described by the RED-S model, with only 9% of females and 18% of males being free of any symptom [103].

Having the above in mind, there is a pivotal need for accurate assessment of actual EE in BP across various periods of the basketball season and to distinguish EE between training and non-training days (or match and non-match days). However, special emphasis should be paid to the methodology of EE evaluation. As observed by Silva et al. in elite young BP [35], there was a great discrepancy between TEE assessed by applying the doubly labeled water method (DLW; 17598 ± 3298 kJ∙day^−1^) and EI obtained via dietary recording (11,274 ± 2567 kJ∙day^−1^); however, no substantial differences between values obtained via DWL and calculations performed according to dietary reference intake (DRI; 17,008 ± 3206 kJ∙day^−1^) were found. Nevertheless, the authors concluded that although the DRI method may be valid for estimating EE at the population or group levels, still it is inaccurate for estimating individual TEE in young players during a demanding competitive season [35]. Thus, BP, especially those at elite and professional levels, should periodically undergo professional evaluation of TEE and exercise EE at different periods of the athletic season, with the use of standard, valid, and accurate methods and equipment and performed by experienced personnel. Furthermore, sports nutritionists and dietitians, as well as athletes themselves, should be trained in adjusting EI to actual EE and differentiate energy (food) intake between training/non-training (match/non-match) days.

### 4.2. Habitual Macronutrients Intake

Apart from clear deficiencies regarding properly covering energy needs, or adjusting EI according to day-to-day or training macro- and micro-cycle variations in EE, the great shortages in proper macronutrients’ contributions in daily EI were also disclosed. Some of the included studies clearly indicated an inappropriate share of macronutrients in the habitual diet of young/junior BP [15,16,24,28], with diets characterized as CHO-rich but relatively low in PRO per energy unit [24]; deficient in CHO, excessive in FAT, and adequate in PRO intake [15,16]; and unbalanced with regard to PRO and CHO and high in FAT [28]. Baranauskas et al. [15] noted an adequate total amount of PRO in the daily diets of young female BP and an inadequate tryptophan, methionine, and lysine ratio (evaluated ratio 1:1.5:4.6; recommended values are 1:3:4). Moreover, the composition of FA was also inadequate, with diets being characterized by high intake of saturated FA (14.7% of TEI with the recommended amount being 10% TEI) and dietary cholesterol and an inappropriate ratio of linoleic to linolenic acids (1:1.8 with the recommended ratio being 1:5) [15]. Furthermore, Nikić et al. [28] observed that about 60% of elite junior BP did not meet recommendations regarding DF intake (with the recommended intake of ~38 g_DF_∙d^−1^).

During basketball matches, athletes perform a variety of high-intensity efforts [3]. Multiple repetitions of these activities lead to the exploitation of muscle glycogen stores, which may, among other effects, result in a decrease in power output and total work during training and competition [104]. Therefore, CHO are the primary fuel during competition for BP [3]. Dietary recommendations released by the International Society of Sports Nutrition (ISSN) [105] for exercise and sports nutrition state that in general CHO intake should be 5–8 g_CHO_∙kg_BM_^−1^∙day^−1^ or 250–1200 g_CHO_∙day^−1^ (for athletes weighing 50–150 kg). Among studies included in the current systematic review, the great majority of them [20,22,25,26,27,30,35,37,38,39] revealed a level of CHO consumption <5 g_CHO_∙kg_BM_^−1^∙day^−1^, and this issue was a case regardless of age group, gender, or presence of disabilities. Moreover, CHO requirements are even higher in athletes engaged in high-volume intense training and may be equal to 8–10 g_CHO_∙kg_BM_^−1^∙day^−1^ or 400–1500 g_CHO_∙day^−1^ (for athletes weighing 50–150 kg). The current systematic review disclosed a lack of proper and sufficient periodization in CHO intake according to daily [25,26] or macro-cycle [27,34,39] variations in energy demands (and consequent variations in macronutrients requirements). Data on periodization in CHO intake within pre- and post-exercise meals in BP is also lacking. The gathered data indicate a relatively common concern of DF deficiency in BP [21,27,29]. Nowak et al. [29] noted extremely low DF intake in adult male and female BP at 3.8 ± 3.6 and 1.9 ± 1.1 g_DF_∙day^−1^, respectively. Nepocatych et al. [27] revealed that mean DF intake in female BP was below the recommendations (25 g_DF_∙day^−1^) both at the beginning (15 ± 4 g_DF_∙day^−1^) and at the end of the season (20 ± 8 g_DF_∙day^−1^), while among Polish adult male BP [21], the recommendations for DF intake were met solely by 22.9% of athletes. Similarly, the included literature pointed out the possible deficiency of DF in the habitual diet of para-athlete BP. In the study by Toti et al. [38], the mean intake of DF was 17.4 ± 1.3 g_DF_∙day^−1^, and 14 out of 15 players consumed <25 g_DF_∙day^−1^. Eskici and Ersoy [19] observed that the mean DF intake in female wheelchair BP was 25.3 ± 8.2 g_DF_∙day^−1^, with only 36.4% of participants meeting the recommended DF level intake. Similar observations were also made in deaf female BP [17], with mean intake (22.6 ± 1.2 g_DF_∙day^−1^) being below recommendations (25 g_DF_∙day^−1^).

According to the ISSN recommendations [105,106], daily PRO intake of 1.4–2.0 g_PRO_∙kg_BM_^−1^∙day^−1^ is sufficient for most exercising individuals. In the opinion of the authors of the current systematic review, the recommendation should be perceived as characterized by a great degree of generality, and individual variations in requirements should always be taken into account. However, in general, in most of the included studies the estimated average habitual daily PRO intake fell within the mentioned range; still there were groups of BP not meeting the recommendation [17,26,29,34,35,37,39]. Nevertheless, apart from the average intake of PRO (and remaining macronutrients) in particular of studied groups, it is necessary to have insight into individual levels of their intake. Nepocatych et al. [27] indicated that 45% of studied female BP did not meet the recommendations for PRO intake (applied recommendations 1.2–1.7 g_PRO_∙kg_BM_^−1^∙day^−1^), while Papandreou et al. [30] revealed proper PRO intake in female BP and insufficient intake in male BP. Another concern is the lack of adequate periodization in PRO intake. Leinus and Ööpik [26] noticed higher PRO, CHO, and FAT intake during resting days compared to training days in both male and female PB; however, solely differences regarding CHO intake in males were statistically significant. Zanders et al. [39] revealed fluctuations in macronutrients intake in female BP across the entire season, with intake of PRO and FAT being higher during the phase of heavy practicing and non-conference games (phase I) compared to the off-season workout phase (phase IV) and CHO intake being higher during the off-season compared to the heavy training phase. No data is available on daily PRO periodization (pre- and post-exercise) in BP.

Dietary recommendations for FAT intake for athletes are similar to or slightly greater than for non-athletic populations to promote health [105], and a moderate amount of FAT of approximately 30% of TEI is acceptable and recommended in athletes [105]. The total amount of FAT in habitual daily diets of included groups of BP varied considerably between each other. However, more alarming are concerns related to the unfavorable profile of dietary FA. Kostopoulos et al. [25] noticed a low contribution of PUFA and a high SFA and monounsaturated fatty acids (MUFA) intake in total fat consumption. Similarly, Schröder et al. [32] revealed the mean share of SFA (13.6% of TEI) to be above the allowed recommended level (<10% of TEI), while excessive cholesterol intake was observed by Gacek [21], Nepocatych et al. [27], Schröder et al. [32], and Gacek and Wojtowicz [82] (41.7% of participants exceeding the level of <300 mg∙day^−1^). Interestingly, Davis et al. [93] evaluated omega-3 status in NBA players. The results showed that the average omega-3 index (O3i), which reflects long-term (~120 previous days) intake of omega-3 FA (especially EPA and DHA), was 5.02 ± 1.19% (2.84–9.76%). Specifically, 21% of athletes had O3i <4% (the value corresponds to high cardiovascular risk), whereas in 77% it ranged from 4 to 8% (intermediate risk), and in solely 2% of players it was >8% (low risk).

Referring to wheelchair and deaf BP, Ferro et al. [20] compared the nutritional value of habitual diet between two high-intensity training camps in a pre-competitive period of the season (May and June). Although there were no significant differences in EI and macronutrients intake between May and June, it was observed that recommendations for CHO intake were reached by six players (55% of participants) in May and nine in June (82%); for PRO by athletes six in May (55%) and seven in June (64%); and for FAT by four participants in May (36%) and nine in June (82%). Interestingly, the profile of FA was significantly improved in June, and it was indicated by an increase in the intake of PUFA, reduction in SFA (two players—18% of participants under the 10% of TEI in May and seven in June [64%]), and cholesterol intake (one player [9%] in May under the amount of 300 mg∙day^−1^ and six in June [55%]). Regarding the profile of FAT intake, excessive intake of SFA was observed in female wheelchair BP [37], with a median intake of 14% of TEI and the consumption of SFA above the recommendations (10% of TEI) in all studied female athletes. The habitual diets of deaf female BP also seem to be characterized by an unfavorable profile of FA intake, with excessive intake of SFA and simultaneous deficiency of PUFA [17].

### 4.3. Hydration—Habits, Practices, and Implications with Basketball Performance

Although there are some inconsistencies in the threshold of dehydration that leads to impairment in performance-determining factors and basketball performance itself among the results of interventional investigations related to dehydration and hydration strategies included in the current systematic review, the lowest value that causes any impairment should be perceived as such a threshold. In these circumferences, DEH equal to 2% must be recognized as causing adverse effects in BP performance. Similarly, the results of the current systematic review provide inconclusive evidence on the type and/or composition of fluids most effective for replenishing fluid loss during exercise or preventing performance decrements during exercise. Referring to comparisons between carbohydrate-electrolyte (CES) and placebo solutions undertaken by Baker and colleagues [42,52] and Dougherty et al. [54], there was a lack of differences in the physiological and biological action of both solutions observed by Baker and colleagues [42,52] but some advantages of CES over PLA in basketball performance in the study by Dougherty et al. [54]. Within all three discussed protocols [42,52,54], participants were provided with standardized meals at the laboratory before the start of test procedures. Although the contribution of macronutrients in standardized meals did not differ between protocols (36% of energy from CHO, 25% from FAT, and 39% from PRO), the absolute amount of macronutrients varied considerably at least between the two protocols [42,54]. In the study by Dougherty et al. [54], the total energy value of the meal was 275 kcal (25 g CHO, 8 g FAT, and 28 g PRO), while in the study by Baker et al. [42] it was 550 kcal (50 g CHO, 16 g FAT, and 56 g PRO). Although the studied groups differed considerably in age and average BM, which were 13.5 ± 1.3 years and 65.3 ± 14.4 kg [54], or 21.1 ± 2.4 years and 81.6 ± 12.1 kg [42], respectively, the differences neither compensated the differentiation of macronutrients provision in standardized meals relative to BM (~0.38 g_CHO_∙kg_BM_^−1^, 0.12 g_FAT_∙kg_BM_^−1^ and 0.43 g_PRO_∙kg_BM_^−1^ in the study by Dougherty et al. [54]; ~0.61 g_CHO_∙kg_BM_^−1^, 0.20 g_FAT_∙kg_BM_^−1^ and 0.60 g_PRO_∙kg_BM_^−1^ in the study by Baker et al. [42]) nor generate such a great diversification in nutritional needs. What is more, taking into account the wide ranges of BM of participants in both studies, the actual individual relative intake of macronutrients with standardized meals, and as a consequence CHO availability during test exercise, differed considerably between study participants. Such differences in absolute and relative macronutrients intake with standardized meals might at least partially contribute to the inconsistency of the effectiveness of CES in affecting performance in basketball drill tests. Thus, it needs to be considered in future studies, to establish the macronutrients’ composition of standardized meals in relation to individual body mass.

Although in none of the included OS on hydration status in BP did pre-exercise (pre-training or pre-game) accidental DEH level reach the threshold of 2%, still, in the majority of observations, there was a high proportion of athletes who began the exercise in an improper hydration state. Among the most commonly used indicators of hydration status is urine specific gravity (USG). However, it needs to be mentioned that various ranges of USG are being utilised for results interpretation. An example can be found in the studies by Thigpen et al. [50] and Heishman et al. [46], where the following interpretation ranges were implemented: ≤1.020 ‘minimal dehydration’, 1.020–1.030 ‘significant dehydration’, >1.030 ‘serious dehydration’ [50] and ≤1.020 ‘euhydration’, 1.020–1.030 ‘hypohydration’, >1.030 ‘significant hypohydration’ [46], respectively. The fact needs to be taken into account when comparing dehydration incidence between studies that the same USG value may be categorized/interpreted differently. However, despite the methodological considerations, it needs to be emphasized that a great majority of BP start practice/matches with some kind of ‘hydration debt’, which with a high degree of probability will worsen during exercise. The results of both, OS and IS indicate that BP do not have the practical ability to replace fluids losses during training and/or games properly (while the data on post-exercise hydration practices are lacking). An alarming and serious example of this phenomenon may be found in the study by Thigpen et al. [50] in which the BM losses during 170 min practice (which in fact was the second training unit during the day) reached 2.5–2.9% in female and male BP. Meanwhile, there is a relatively high number of observations on sweating rates during various types of basketball practice [40,44,45,50,51]. Those studies revealed that sweating rates in various groups of BP range from ~0.6 to ~2.7 L∙h^−1^, and it depends on the type of training, season of the year, or gender. The knowledge of sweating rate might be useful in developing basketball-specific hydration strategies during training and competition games or for recovery periods. Nevertheless, the up-to-date data from experimental studies on BP seem to justify solely framing the following recommendations: (1) BP should have ad libitum access to fluid during training and competition; (2) the type of the fluid should be individually chosen according to one’s organoleptic preference and gastrointestinal tolerance; (3) the advantage of CES over non-energetic fluids probably depends on individual and actual CHO availability during practice and/or potential ‘hydration debt’.

### 4.4. Macronutrients’ Alternations and Manipulations

The evidence from the included IS on PRO supplementation allows us to state that, with respect to body composition and basketball performance, athletes may benefit from long-term (8 weeks) supplementation with whey PRO [69,70], while a single ingestion of a high-protein and CHO drink (36% PRO contribution, 58% CHO, and 6% FAT) may contribute to improved post-exercise recovery and performance in subsequent physical efforts [63]. However, the latter mentioned figures come from a study evaluated as having a high risk of bias. Simultaneously, a single ingestion of a meal consisting of CHO + PRO (1 g_PCHO_∙kg_BM_^−1^ + 1 g_PRO_∙kg_BM_^−1^) compared to a meal consisting of CHO only (2 g_CHO_∙kg_BM_^−1^) may be beneficial in selected components of basketball performance during the final stages (4th QR) of performance protocols [61]. Having the above in mind, it seems reasonable to consider the implementation of supplementation with whey protein (2 × 24 g∙day^−1^) during the preparatory macro-cycle of the season and continue it until the end of the competition period. What is more, the additional pre-exercise provision of high-protein drinks or carbohydrate-protein meals on days with repeated bouts of heavy training units or competition may be considered.

Based on the current systematic review, no strict and discipline-specific recommendations on CHO intake—its quality, type, quantity, timing, or periodization of ingestion—could be stated. Solely single protocols investigating differential aspects of CHO intake have recently been implemented in BP. Moreover, the protocols evaluated a broad range of various outcomes. Although dietary interventions implemented by Michalczyk and colleagues [65,66]—4 weeks of LCD followed by 7 days of CHO loading—constituted a significant alternation in macronutrients’ contribution into daily EI compared to conventional diets of studied athletes, in fact, it is unclear what kind of physiological responses they were assumed to evoke in the first place. Neither the proportion of macronutrients during LCD nor the concentration of β-hydroxybutyrate (βHB) after 4 weeks of LCD [66] allow classification of the diet as a ketogenic diet. A βHB concentration of 0.5–3.0 mmol·L^−1^ is assumed as the threshold for a nutritional ketosis state [107,108,109], while in the discussed study [66], the LCD resulted in a βHB concentration equal to 0.161 ± 0.11 mmol·L^−1^. Thus, the adaptive response typical to a ketogenic diet could not be expected. Little is known about the actual glycemic index of introduced LCDs [65,66]. Thus, it is reasonable and necessary for future studies to implement dietary interventions (alternations in macronutrients’ quantity and quality) based on possible and well-thought-out physiological adaptations as a foundation (highly specific research hypothesis) and to explore assumed performance or recovery implications of those physiological adaptations. With respect to the studies on single acute pre-exercise administration of CHO, similar to the studies on hydration strategies utilising CES, special attention should be paid to individualization of macronutrients provision according to BM and thus on actual CHO availability during exercise, as well as on individual gastrointestinal tolerance of high-CHO meals.

Furthermore, regarding FAT-related nutrients such as PUFA intake, the available data indicate that BP [62], including wheelchair athletes [64], may benefit from long-term co-supplementation of EPA/DHA and vitamin E with respect to the inflammatory and antioxidant status of the body [62,64]. However, no consequent performance advantages of such treatment have been studied.

### 4.5. Studies on Para-Athlete Basketball Players

Studies on para-athlete BP constituted a relatively high proportion of the total number of included studies—9 out of 72 studies (12.5%)—and 3 of them were IS. However, two studies focused exclusively on dietary counseling interventions [22,37], and solely one was a dietary supplementation protocol [64]. The lack and the need for specific dietary recommendations for wheelchair BP has been raised recently [110]. Compared to able-bodied BP, wheelchair BP have different nutritional needs. EE during wheelchair basketball practice are lower compared to ‘conventional’ basketball [110]. EE is higher in wheelchair basketball compared to other paralympic sports [110]. Moreover, in individuals with paraplegia, due to reduced functionality of the nervous system, gastric emptying is slower, which delays the absorption of nutrients along with a restricted synthesis of adrenaline and noradrenaline and a lower rate of lipolysis in adipose tissue [110]. Furthermore, wheelchair BP, especially those with spinal cord injuries, as a consequence of impairment of the cutaneous vasodilation and decreased activation of the sweat glands, are at increased risk of thermoregulatory disturbances [111]. Thus, special attention should be paid to proper and individually tailored hydration strategies, regarding both the amount and the composition of fluids. Having the above in mind, there is no doubt that, regarding wheelchair BP, it is impossible to adapt dietary recommendations framed for able-bodied BP or for paralympic athletes of other sports disciplines. The need for IS related to effective performance-enhancing nutritional strategies in para-athlete basketball players is therefore indisputable.

### 4.6. Strengths and Limitations

This is the first systematic review that discusses the concerns related to the basics of sports nutrition in basketball in such a broad context. A strength of this review is the inclusion of a large number of both OS and IS, which deliver a complementary insight into problems and shortages related to implementation and provision of proper nutritional practices in various groups of BP. The inclusion of OS allowed us to present the actual nutritional and hydration practices of BP and to disclose many irregularities in this regard. Moreover, based on the evaluation of eating habits and behaviours, as well as NK of BP, the reasons for widespread irregularities in covering nutritional needs could be disclosed and exploited for the future development of well-tailored nutritional and hydration education programs for basketball practitioners and coaching staff. On the other hand, the inclusion of experimental studies disclosed the dramatically low number of relevant investigations that could provide reliable proof for framing basketball-specific evidence-based nutritional recommendations related to the basics of sports nutrition. Eventually, the inclusion of both OS and IS was necessary for a proper and comprehensive indication of the direction of future studies.

Another strength of this review is the inclusion of a broad range of various groups of BP. The group-specific shortages in NK and covering nutritional needs were disclosed and must be taken into account when performing future investigations and nutritional education activities in particular groups of BP. Based on the results of the current systematic review, it seems to be pivotal to introduce educational courses on the basics of sports nutrition and dietary counseling starting from the beginning of a sports career in junior BP, while numerous adverse lifestyle and nutritional behaviours were detected in those particular groups of athletes. It must be emphasized that educational activities need to be undertaken in junior BP to prevent transferring adverse nutritional practices for the latter years of life and of sports practice.

The current systematic review focused ‘solely’ on the basics of sports nutrition in basketball. Supplementation protocols other than those related to CHO, PRO, FAT, and fluids alternations were beyond the scope of the current systematic review. This can be perceived as a limitation. Nevertheless, the magnitude and the volume of the gathered data are relatively high, and the data integrally cover basic concerns related to proper nutrition in basketball. Moreover, the authors of the current systematic review are of the opinion that proper nutritional guidance of any sports practitioner (regardless of current stage of sports career) should account for familiarization with essential foundations of discipline-specific sports nutrition, which will contribute to the development of proper nutritional habits and the proper covering of energy and nutritional needs. This can serve as a strong foundation, ensuring adequate health and physical development, as well as support of physical performance, and can serve as a fair start-up for introducing further ergogenic supplementation strategies. 

Another limitation may be the fact that the quality of included studies, specifically the RoB, was in general rated as ‘high’ or as burdened with ‘some concerns’. Still, this aspect is beyond any dependence of the authors of the review. In fact, the efforts invested in evaluating the RoB of the included studies clearly demonstrated the urgent need for methodologically well-planned, designed, and carried-out OS and interventional protocols related to the basics of multidimensional sports nutrition in basketball. Still, there is a need for reliable data on habitual energy and macronutrients intake in BP, while even the authors of some of the included records indicated an underestimation in the dietary recording. Another concern arising from the results of RoB evaluation is the lack of standardization in performing dietary evaluation across the studies (and sometimes within the studies), as well as the lack of validated sport-specific tools for investigating the nutritional habits of players. The need for sport-specific tools for diet evaluation has recently been also noticed by Capling and colleagues [112,113]. This area of nutritional evaluation in physically active individuals and athletes should undoubtedly be extensively developed in the near future.

Unfortunately, none of the included randomized controlled trials was evaluated as having ‘low’ RoB. The greatest number of concerns regarding the RoB in the mentioned type of studies arose from ‘deviations from intended interventions’ and ‘selection of the reported results’. It is worth discussing the latter of the mentioned concerns in light of the two studies by Baker and colleagues [42,52]. They were both methodologically well-considered and planned and successfully implemented comprehensive protocols investigating different levels of DEH and two various EUH strategies (EUH-CES and EUH-PLA) vs. various aspects of cognitive and physical performance. However, eventually due to a lack of differences between the outcomes of EUH-CES or EUH-PLA, results for EUH-CES were excluded from the presentation in the paper [42] or results were presented as the means of two implemented EUH conditions [52]. Thus, the described procedures were considered ‘as a selection of the reported results’. However, it needs to be clearly underlined that at the time of the publication of a part of the included studies, neither the recommendations and guidelines regarding performing and reporting randomized controlled trials nor the tools for their quality assessment were developed and precisely described. In these circumstances, according to the ‘*Lex retro non agit*’ rule, it is worth considering if and to what extent the ‘new’ tools for RoB evaluation are applicable to the papers/protocols published before their development. On the other hand, among the included records, there are some with simply a poor quality of reporting study protocol and/or results. For instance, in the study by Shi [68] it is not clear whether the supplementation protocol lasted for 9 or 10 days. In fact, such uncertainties in reporting study protocol and results should discount the record from inclusion in systematic reviews. However, taking into account the methodological aspect of the current systematic review, the authors decided not to decline the discussed record.

## 5. Conclusions

The current systematic review provides an extensive compilation of knowledge especially valuable for athletes, coaches, nutritionists/dietitians, medical staff, as well as scientists and academics. Based on the thoughtful analyses, the following recommendations may be framed:

(1) Each basketball player should periodically undergo professional evaluation of exercise- and total energy expenditures and physical activity levels during different periods of the athletic season, with the use of standard, valid, and accurate methods and equipment and performed by trained and experienced personnel.
(2) Special emphasis should be paid to the proper periodization of energy and macronutrients’ intake according to training macro- and microcycles, training/non-training (match/non-match) days, as well as timing of meal consumption according to pre- and post-exercise schedule. Energy and macronutrients must be adjusted to actual and individual athletic requirements. Proper provision of CHO is of particular importance. However, the results of the current systematic review do not allow for framing basketball-specific recommendations on CHO intake; thus, the athletes should follow the most up-to-date recommendations for the general athletic population.
(3) Basketball players at each age, level of training experience, or degree of full-body abilities must be provided with nutritional education courses. Taking into account numerous adverse lifestyle and nutritional behaviours in junior basketball players, these activities need to be undertaken from the very beginning of their sports career to prevent transferring adverse nutritional practices to later years of life and sports practice, as well as for developing proper diet, health, and lifestyle behaviours that ensure optimal growth and physiological and physical development.
(4) Based on the included studies, modifying and improving eating habits in basketball players seem to be a longer term process. Thus, nutritional education courses should be planned as longer-lasting programs and should comprise group and individual meetings. Finally, periodical monitoring of their effectiveness should also be introduced.
(5) The scope of nutritional education should be individually tailored to specific and pre-identified needs of particular groups of basketball players. Nevertheless, based on the results of current systematic review, special attention should be paid to (a) nutritional characteristics of particular food groups and proper frequency of their consumption and distribution between meals, including pre- and post-exercises eating occasions; (b) making athletes aware of the importance and necessity of proper periodization (at a micro and macro scale) and timing in energy and macronutrients’ intake; (c) providing them with basic abilities to estimate energy and macronutrients’ intake with particular foods; (d) proper hydration practices, including self-evaluation of an adequate pre-exercise hydration status and exercise-induced fluid losses based on simple indices e.g., urine colour or body mass change, respectively; fluid replenishment strategies during trainings/competitions; and post-exercise rehydration protocols; (e) the importance of proper nutritional and hydration practices on the days of heavy trainings/competition, especially due to the fact that they are commonly neglected by the athletes particularly at such occasions.

Based on the results of the current systematic review, the following recommendations and guidelines regarding methodological considerations for future studies on the basics of applied sports nutrition in basketball must be specified:

(1) Sport-specific tools for the evaluation of diet, eating habits, or nutritional knowledge must be developed, validated, and widely introduced in the research practice.
(2) Any dietary intervention or supplementation protocol in basketball players needs to originate from a well-thought-out and clearly pre-specified research hypotheses, and the hypotheses must be supported by the underlying presumable—but probable—physiological mechanisms. No random protocols can be implemented.
(3) To frame any basketball-specific dietary recommendations, there is apparently the necessity for conducting interventional studies on alternations/supplementation with carbohydrate, protein, and fat, while considering acute, short-, and/or long-term protocols, as well as aspects related to diet periodization and consumption timing.
(4) With respect to hydration strategies, special emphasis must be paid to post-exercise rehydration protocols, while none of the up-to-date studies in basketball players have investigated this aspect. Concurrently, the concern related to the most effective fluids in replenishing fluids losses during exercise in basketball players is still to be resolved. The factors determining the applicability of carbohydrate-electrolyte solutions concerning actual discipline-specific performance need to be disclosed and described in various groups of basketball players.
(5) Protocols of interventional studies, including a plan of statistical analysis, must be prospectively registered in the relevant databases. The presentation of study results need to cover all pre-specified outcomes and all studied subgroups/treatments.

## Figures and Tables

**Table 1:** Habitual energy and macronutrients intake.

Reference	Survey Method	Season/Training Macrocycle	Gender/*n*	Age (Years)	Body Mass (kg)	Energy		Carbohydrate			Protein			Fat		
						(kcal∙day^−1^)	(kcal∙kg_BM_^−1^∙day^−1^)	(g∙day^−1^)	(g∙kg_BM_^−1^∙day^−1^)	(% EI)	(g∙day^−1^)	(g∙kg_BM_^−1^∙day^−1^)	(% EI)	(g∙day^−1^)	(g∙kg_BM_^−1^∙day^−1^)	(% EI)
Baranauskas et al. 2021 [15] ^§^	3 × 24 h FD	-	F/10	16.2 ± 0.4	70.6 ± 4.6	2781 ± 210	40.0 ± 3.8	*353* *^ǂ^*	5.0 ± 0.4	50.3 ± 1.9	*106* *^ǂ^*	1.5 ± 0.2	15.0 ± 0.5	*113* *^ǂ^*	1.6 ± 0.2	34.7 ± 1.5
Baranauskas et al. 2013 [16]	24 h DR	training mezzo-cycles designed for strength training	M/39 F/13	18.6 ± 1.8 16.1 ± 0.5	85.7 ± 9.9 69.5 ± 8.0	4521.2 ± 1341.7 2854.5 ± 428.1	52.9 ± 14.8 41.6 ± 8.4	*514 ^ǂ^* *375 ^ǂ^*	6.0 ± 1.9 5.4 ± 1.3	44.8 ± 5.4 52.0 ± 5.1	*163 ^ǂ^* *104 ^ǂ^*	1.9 ± 0.6 1.5 ± 0.3	-	-	-	40.7 ± 5.2 33.5 ± 4.2
Baranauskas et al. 2020 [17]	7-day DR	special preparatory period for the competition	F/14	26.4 ± 4.5	65.2 ± 7.8	2579 ± 590	*39.6* *^ǂ^*	*326* *^ǂ^*	5.0 ± 1.3	-	*85* *^ǂ^*	1.3 ± 0.3	-	-	-	38.1 ± 4.1
Dzimbova 2020 [18]	FFQ	-	-/16	15.4 ± 1.2	64.4 ± 10.8	2204 ± 624	*32.2* *^ǂ^*	379.7 ± 123.4	*5.9* *^ǂ^*	-	90.8 ± 28.9	*1.4* *^ǂ^*	-	59.8 ± 17.3	*0.9* *^ǂ^*	-
Eskici and Ersoy 2016 [19]	24 h DR	athletes at the training camp	F/22	25.5 ± 7.2	57.4 ± 8.6	2867.8 ± 523.6	*50.0* *^ǂ^*	297.3 ± 74.9	*5.2* *^ǂ^*	42.7 ± 8.8	92.6 ± 16.7	*1.6* *^ǂ^*	13.2 ± 1.9	142.7 ± 37.7	*2.5* *^ǂ^*	44.05 ± 8.0
Ferro et al. 2017 [20]	3-day FD on CD (food weighing)	pre-competitive period: May June	M/11	30 ± 6	74.8 ± 14.9 75.1 ± 14.5	2492 ± 362 2470 ± 497	34.8 ± 9.8 34.7 ± 12.6	*281 ^ǂ^* *318 ^ǂ^*	3.76 ± 1.30 4.24 ± 1.92	45.3 ± 7.3 49.3 ± 8.2	*126 ^ǂ^* *111 ^ǂ^*	1.68 ± 0.64 1.48 ± 0.45	19.1 ± 4.8 17.0 ± 2.8	*104 ^ǂ^* *92 ^ǂ^*	1.39 ± 0.43 1.23 ± 0.41	35.5 ± 4.7 32.1 ± 5.3
Gacek 2022 [21]	3-day FD (2 TDs + 1 RD)	-	M/48	26.6 ± 4.5	-	1795.5 ± 547.9	-	258.2 ± 105.9	-	52.4 ± 9.2	79.3 ± 23.4	-	18.2 ± 3.0	58.5 ± 24.5	-	29.4 ± 9.4
Grams et al. 2016 [22] ^§^	3-day FD on CD (food weighing)	training camps held in the pre-competitive season	M/8	29.9 ± 6.5	75.0 ± 16.2	2441 ± 341	*32.5* *^ǂ^*	*233* *^ǂ^*	3.1	-	*120* *^ǂ^*	1.6 ± 0.7	-	-	-	35.1 ± 4.7
Hickson et al. 1986 [23]	3 × 24 h DR on CD weekdays	competitive season	F/13	19.4 ± 0.3	68.3 ± 1.6	1995 ± 151 (SEM)	30 ± 8	-	-	-	-	-	-	-	-	-
Hickson et al. 1990 [24]	3-day FD on CD weekdays (food weighing)	pre-season	M/12	16.4 ± 0.7 15–18	77.0 ± 8.9	3400 ± 702	45 ± 10	-	-	53	-	-	13	-	-	34
Kostopoulos et al. 2017 [25]	3 × 24 h DR on 2 non-CDs (1 weekday and 1 weekend day) + 1 match day	competitive season (playoff stage): average from 3 × 24 h DR intake at the match day	-/18	24 ± 4	-	-	24.7 ± 7.2 24.0 ± 8.7	-	2.6 ± 0.8 2.8 ± 0.8	45.3 ± 10.5 45.7 ± 11.9	-	1.5 ± 0.9 1.4 ± 1.0	23.0 ± 7.4 22.7 ± 9.7	-	0.95 ± 0.36 0.88 ± 0.33	33.9 ± 5.9 33.3 ± 6.2
Leinus and Ööpik 1998 [26]	4-day FD on 2 TDs + 2 RDs (food weighing)	RD TD RD+TD RD TD RD + TD	M/7 F/7	21.1 ± 2.6 20.6 ± 1.9	81.6 ± 9.3 63.1 ± 7.7	3545 ± 970 2531 ± 719 2986 ± 767 2185 ± 666 1752 ± 394 1968 ± 449	*43.4 ^ǂ^* *31.0 ^ǂ^* *36.6 ^ǂ^* *34.6 ^ǂ^* *27.8 ^ǂ^* *31.2 ^ǂ^*	384 *^ǂ^* *294 ^ǂ^* *335 ^ǂ^* *252 ^ǂ^* *221 ^ǂ^* *240 ^ǂ^*	4.7 ± 1.3 3.6 ± 1.1 4.1 ± 0.9 4.0 ± 1.3 3.5 ± 1.1 3.8 ± 0.9	43.0 ± 9.1 46.7 ± 9.4 44.9 ± 8.1 47.0 ± 5.0 48.4 ± 5.4 47.7 ± 4.5	*114 ^ǂ^* *81 ^ǂ^* *90 ^ǂ^* *57 ^ǂ^* *50 ^ǂ^* *50 ^ǂ^*	1.4 ± 0.4 1.0 ± 0.3 1.1 ± 0.2 0.9 ± 0.2 0.8 ± 0.3 0.8 ± 0.2	13.0 ± 2.7 12.8 ± 2.4 12.9 ± 1.7 11.4 ± 1.5 11.5 ± 2.0 11.5 ± 1.3	*180 ^ǂ^* *122 ^ǂ^* *147 ^ǂ^* *107 ^ǂ^* *76 ^ǂ^* *95 ^ǂ^*	2.2 ± 0.7 1.5 ± 0.6 1.8 ± 0.6 1.7 ± 0.7 1.2 ± 0.3 1.5 ± 0.5	44.1 ± 8.5 40.5 ± 7.4 42.3 ± 7.4 41.7 ± 5.8 40.2 ± 6.1 41.0 ± 5.2
Nepocatych and Balilionis 2017 [27]	3-day FD (2 week- and 1 weekend day)	beginning of the competitive season end of the competitive season	F/10	18–22	78.7 ± 16.8 80.1 ± 18.6	2208 ± 373 2567 ± 834	29 ± 8 34 ± 15	254 ± 51 304 ±74	3.4 ± 1.0 4.1 ± 1.5	46 ± 6 50 ± 14	92 ± 29 97 ± 38	1.3 ± 0.6 1.4 ± 0.7	17 ± 5 15 ± 2	87 ± 19 111 ± 42	1.2 ± 0.3 1.6 ± 0.9	35 ± 5 39 ± 7
Nikić et al. 2014 [28]	FFQ	-	M/57	15.6 ± 0.9	78.0 ± 10.7	3962 ± 1376	51.1 ± 16.5	487.8 ± 171.8	6.3 ± 2.1	-	140.0 ± 58.2	1.8 ± 0.7	-	165.6 ± 64.4	*2.1* *^ǂ^*	-
Nowak et al. 1998 [29]	3-day FD on CD (weekdays)	pre-competitive season	M/16 F/10	18.9 ± 1.29 19.4 ± 0.97	83.4 ± 9.09 71.7 ± 3.50	3558 ± 1078 1730 ± 573	*42.7 ^ǂ^* *24.1 ^ǂ^*	437 ± 158 229 ± 95	*5.2 ^ǂ^* *3.2 ^ǂ^*	48 52	159 ± 70 68 ± 28	*1.9 ^ǂ^* *0.9 ^ǂ^*	17 16	139 ± 48 63 ± 19	*1.7 ^ǂ^* *0.9 ^ǂ^*	34 32
Papandreou et al. 2007 [30]	5-day FD (week- and weekend days)	-	M/8 F/13	20 ± 4 25 ± 5	90 ± 9 62 ± 8	1901 ± 323 1487 ± 636 (*n* = 8)	21 ± 4 25 ± 13	220 ± 42 170 ±71	1.9 ± 1.1 2.9 ± 1.1	46 ± 3 47 ± 11	80 ± 10 66 ± 24	1.1 ± 0.9 1.1 ± 0.9	17 ± 2 18 ± 5	83 ± 17 64 ± 36	1.1 ± 0.9 1.1 ± 0.9	39 ± 4 36 ± 9
Quintas et al. 2003 [31]	5-day FD (week- and weekend days)	-	F/26	17.2 ± 2.1	70.5 ± 11.02	*2580 ± 698 ^ǂ^* (10,807 ± 2921 kJ∙day^−1^)	*36.6 ^ǂ^* *(153 kJ∙kg^−1^∙day^−1^ ^ǂ^)*	-	-	-	*99* *^ǂ^*	1.4 ± 0.41	-	-	-	-
Schröder et al. 2004 [32]	24 h DR	training & competition	M/50	25.1 ± 4.0	93.0 ± 11.0	*4228 ± 215 ^ǂ^* (17.7 ± 0.9 MJ∙day^−1^)	*45.8 ^ǂ^* (191.8 ± 68.6 kJ∙kg^−1^∙day^−1^)	424.2 ± 165.9	4.6 ± 1.7	40.3 ± 7.7	211.3 ± 99.5	2.3 ± 1.0	19.7 ± 4.9	185.3 ± 78.6	2.1 ± 0.92	39.0 ± 7.7
Shimizu et al. 2019 [33]	FFQ	-	F/13	28.9 ± 8.1	-	1636.1 ± 439.5	-	-	-	-	57.5 ± 18.9	-	-	-	-	-
Silva et al. 2012 [34]	7-day FD	longitudinal approach over 34 weeks: beginning of the pre-season competitive period assessment beginning of the pre-season competitive period assessment	M/7 F/2	16.0 ± 0.5 16.8 ± 0.7 16.3 ± 0.5 16.8 ± 0.7	77.7 ± 6.6 79.9 ± 6.8 64.3 ± 7.1 65.7 ± 6.5	*3003 ± 831 ^ǂ^* (12,570 ± 3478 kJ∙day^−1^) *3239 ± 422 ^ǂ^* (13,559 ± 1765 kJ∙day^−1^) *2392 ± 382 ^ǂ^* (10,015 ± 1600 kJ∙day^−1^) *1801 ± 49 ^ǂ^* (7537 ± 204 kJ∙day^−1^)	*38.7 ^ǂ^* (162 kJ∙kg^−1^∙day^−1^*^ǂ^)* *40.6 ^ǂ^* (170 kJ∙kg^−1^∙day^−1^) *37.3 ^ǂ^* (156 kJ∙kg^−1^∙day^−1 ǂ^) *27.5 ^ǂ^* (115 kJ∙kg^−1^∙day^−1 ǂ^)	395 ± 125 427 ± 81 333 ± 71 227 ± 13	*5.1 ^ǂ^* *5.3 ^ǂ^* *5.2 ^ǂ^* *3.5 ^ǂ^*	-	143 ± 27 150 ± 19 104 ± 17 82 ± 14	*1.8 ^ǂ^* *1.9 ^ǂ^* *1.6 ^ǂ^* *1.2 ^ǂ^*	-	95 ± 28 104 ± 16 72 ± 19 63 ± 3	*1.2 ^ǂ^* *1.3 ^ǂ^* *1.1 ^ǂ^* *0.96 ^ǂ^*	-
Silva et al. 2013 [35]	7-day FD	competitive period	M/12 F/7	17.0 ± 0.7 16.9 ± 0.7	80.9 ± 7.7 64.0 ± 5.4	2895 ± 479 1807 ± 46	*35.8 ^ǂ^* *28.2 ^ǂ^*	365.5 ± 64.4 218.8 ± 1.8	*4.5 ^ǂ^* *3.4 ^ǂ^*	50.5 ± 3.8 48.4 ± 0.8	135.4 ± 23.5 82.0 ± 14.3	*1.7 ^ǂ^* *1.3 ^ǂ^*	18.7 ± 2.8 18.8 ± 2.7	93.5 ± 20.7 64.1 ± 1.2	*1.2 ^ǂ^* *1.0 ^ǂ^*	29.1 ± 2.4 31.4 ± 1.4
Silva et al. 2017 [36]	DXA/DLW	competitive phase	-/24	-	-	4347 ± 756	-	-	-	-	-	-	-	-	-	-
Toti et al. 2021 [37] ^§^	3-day FD on CD (2 working days + 1 weekend day/holiday)	-	M/16 M/12 F/9	27 (24–31) 19 (18–21) 26 (19–30)	74.2 ± 12.3 57.2 ± 11.7 61.0 ± 10.6	*2441 ^ǂ^* *1853 ^ǂ^* *1635 ^ǂ^*	32.9 ± 10.4 32.4 ± 8.8 26.8 ± 5.1	*267 ^ǂ^* *257 ^ǂ^* *226 ^ǂ^*	3.6 ± 1.2 4.5 ± 1.5 3.7 ± 0.9	43.0 55.0 55.0	*11 1 ^ǂ^* *74 ^ǂ^* *61 ^ǂ^*	1.5 (1.1–2.0) 1.3 (1.1–1.4) 1.0 (0.8–1.0)	19.0 17.0 17.0	*96 ^ǂ^* *57 ^ǂ^* *55 ^ǂ^*	1.3 ± 0.4 1.0 ± 0.3 0.9 ± 0.2	37.0 27.0 27.0
Toti et al. 2021 [38]	3-day FD on CD (2 working days + 1 weekend day/holiday)	Training camp before the 2019 European Championship	M/15	28.5 ± 1.5	74.8 ± 3.2	*2438* *^ǂ^*	32.6 ± 2.8	*269* *^ǂ^*	3.6 ± 0.3	43.9 ± 1.2	*112* *^ǂ^*	1.5 ± 0.1	18.4 ± 0.7	-	-	36.9 ± 0.6
Zanders et al. 2021 [39]	4 CD of recording of food intake via mobile app	Entire women’s collegiate basketball season: phase I (heavy practicing + non-conference games) phase II (heavy practicing + conference league play) phase III (postseason conference tournament) phase IV (off-season workout) phase V (off-season workout)	F/13	19.8 ± 1.3	74.6 ± 9.1	2506 ± 271 2354 ± 533 2326 ± 456 2517 ± 334 2422 ± 276	33.7 ± 3.1 31.9 ± 7.8 31.5 ± 7.3 33.8 ± 3.7 32.7 ± 4.9	282.4 ± 60.3 272.2 ± 73.2 244.8 ± 42.2 299.9 ± 36.4 263.2 ± 36.8	3.8 ± 0.7 3.7 ± 1.1 3.3 ± 0.7 4.0 ± 0.4 3.6 ± 0.7	-	97.9 ± 18.8 87.3 ± 13.9 87.5 ± 17.0 78.0 ± 13.9 84.7 ± 16.3	1.31 ± 0.22 1.18 ± 0.19 1.19 ± 0.28 1.05 ± 0.19 1.15 ± 0.26	-	113.0 ± 26.1 98.4 ± 27.1 112.7 ± 29.3 87.3 ± 18.8 93.3 ± 28.5	1.50 ± 0.34 1.37 ± 0.38 1.51 ± 0.42 1.23 ± 0.31 1.23 ± 0.35	-

Abbreviations: CD, consecutive days; DLW, doubly labeled water; DR, dietary recall; DXA, dual-energy X-ray absorptiometry; EI, energy intake; F, female; FD, food diary; FFQ, food frequency questionnaire; M, male; RD, resting days; SEM, standard error of a mean; TD, training days; ^§^ Baseline data extracted from papers reporting on interventional studies [15,22,37]; *^ǂ^* values calculated by the authors of current review based on original mean data given in the corresponding papers (in *italics*).

**Table 2:** Habitual hydration practices and hydration status in basketball players during different types of basketball practices.

Reference	Gender/*n* Age (years)	Body Mass (kg)	Environmental Conditions: Temperature & Relative Humidity	Type of Practice & Duration	Fluid Intake	Indices of Hydration State	Additional Notes
Abbasi et al. 2021 [40] ^§^	F/10 n/a	n/a	n/a n/a	n/a	553.35 ± 122.91 mL (during practice)	**PRE-PRACTICE** Urine specific gravity (USG): 1.017 ± 0.006 Urine colour (UC): 4 ± 1 Incidence of dehydration (DEH)^†^: 40% **POST-PRACTICE** USG: 1.021 ± 0.005; UC: 5 ± 2 Incidence of DEH ^†^: 60% Body mass (BM) loss ^¥^: −0.6 ± 0.3% Sweat rate: 0.6 ± 0.1 L∙h^−1^ Fluid replacement: 59.4 ± 27.3% Hydration Awareness Questionnaire: 121 ± 8	-
Arnaoutis et al. 2015 [41]	M/12 15.5 ± 0.5	78.8 ± 8.9	28.8 °C n/a	A typical day of training 86.0 min	n/a	**FIRST MORNING URINE SAMPLE** USG: 1.026 ± 0.005; UC: 5.0 ± 1.0 **PRE-TRANING** USG: 1.024 ± 0.005; UC: 4.0 ± 1.0 Incidence of euhydration (EUH) ^ǂ^: 16.7% **POST-TRAINING** USG: 1.026 ± 0.005; UC: 5.0 ± 1.0 BM loss ^¥^: −1.0 ± 0.01% or −0.79 ± 0.01 kg	-
Baker et al. 2007 [42] ^§^	M/17 21.1 ± 2.4 (17–28)	81.6 ± 12.1 (63.6–104.5)	n/a n/a	-	-	**FIRST EVALUATION** UC: 5 ± 1; USG: 1.024 ± 0.004; urine osmolality (UO): 820 ± 210 mOsm∙L^−1^ **SECOND EVALUATION** UC: 5 ± 1; USG: 1.022 ± 0.006; UO: 774 ± 201 mOsm∙L^−1^ **THIRD EVALUATION** UC: 5 ± 1; USG: 1.023 ± 0.005; UO: 795 ± 180 mOsm∙L^−1^ **FOURTH EVALUATION** UC: 5 ± 1; USG: 1.025 ± 0.006; UO: 826 ± 181 mOsm∙L^−1^ **FIFTH EVALUATION** UC: 5 ± 2; USG: 1.021 ± 0.006; UO: 771 ± 240 mOsm∙L^−1^	Baseline measurements were taken on five different occasions before introducing different hydration/dehydration strategies
Barnes et al. 2019 [43]	M, F/196 23 ± 5	92.1 ± 18.0	22.4 ± 1.7 °C 51 ± 12%	n/a 2.1 ± 0.8 h	n/a	Whole body sweat loss: 0.95 ± 0.42 L∙h^−1^ Whole body sweat [Na+]: 35.4 ± 11.2 mmol∙L^−1^ Rate of sweat Na^+^ loss: 34.5 ± 21.2 mmol∙h^−1^	-
Brandenburg and Gaetz 2012 [44]	F/17 24.2 ± 3	78.8 ± 8	22.5–23.5 °C 44–50%	**GAME I (preceded by 40-min warm-up)** 17.0 ± 4.4 min	**GAME I** Warm-up: 0.35 ± 0.2 L Game: 1.22 ± 0.5 L Fluid intake in relation to sweat loss: 77.8 ± 32%	**GAME I** Pre-game USG: 1.005 ± 0.002 (1.002–1.008) Sweat loss: −1.99 ± 0.75 L BM loss ^¥^: −0.7 ± 0.8 (−2.1–0.5)% BM loss ^¥^: −0.6 ± 0.5 (−1.5–0.4) kg	Two games played on consecutive days against the same opponent
				**GAME II (preceded by 40-min warm-up)** 16.4 ± 4.7 min	**GAME II** Warm-up: 0.25 ± 0.1 L Game: 1.40 ± 0.6 L Fluid intake in relation to sweat loss: 78.0 ± 21%	**GAME II** Pre-game USG: 1.010 ± 0.005 (1.005–1.022) Sweat loss: −1.99 ± 0.60 L BM loss ^¥^: −0.6 ± 0.6 (−2.0–0.1)% BM loss ^¥^: −0.5 ± 0.5 (−1.6–0.1) kg	
Broad et al. 1996 [45]	M/19 16.0–18.0	92.65 ± 8.33	**WINTER**	**WINTER**	**WINTER**	**WINTER**	Testing sessions represent a typical program of weight training, field training, and competition sessions over a 1-week period; data were collected during a minimum of two matches, four training sessions, and two weight training sessions
			20.1 ± 0.0 °C 37.0 ± 0.2%	Weight training session -	113 ± 149 mL∙h^−1^	Sweat rate: 337 ± 120 mL∙h^−1^ BM loss ^¶^: −0.4 ± 0.3%	
			19.9 ± 1.4 °C 24.1 ± 3.3%	Court/field training 123 ± 18 min	489 ± 177 mL∙h^−1^	Sweat rate: 1039 ± 169 mL∙h^−1^ BM loss ^¶^: −1.2 ± 0.4%	
			18.9 ± 0.9 °C 36.3 ± 5.8%	Competition 85 ± 24 min	917 ± 460 mL∙h^−1^	Sweat rate: 1587 ± 362 mL∙h^−1^ BM loss ^¶^: −1.0 ± 0.6%	
			**SUMMER**	**SUMMER**	**SUMMER**	**SUMMER**	
			22.5 ± 0.0 °C 52.1 ± 6.4%	Weight training session -	236 ± 292 mL∙h^−1^	Sweat rate: 389 ± 121 mL∙h^−1^ BM loss ^¶^: −0.3 ± 0.4%	
			27.4 ± 2.5 °C 33.7 ± 6.3%	Court/field training 103 ± 38 min	797 ± 234 mL∙h^−1^	Sweat rate:1371 ± 235 mL∙h^−1^ BM loss ^¶^: −1.0 ± 0.5%	
			23.3 ± 2.6 °C 41.4 ± 10.6%	Competition 89 ± 21 min	1079 ± 613 mL∙h^−1^	Sweat rate: 1601 ± 371 mL∙h^−1^ BM loss^¶^: −0.9 ± 0.7%	
	F/12 16–18	68.16 ± 5.42	**WINTER**	**WINTER**	**WINTER**	**WINTER**	
			20.9 °C 65.9%	Weight training session -	23 ± 60 mL∙h^−1^	Sweat rate: 246 ± 133 mL∙h^−1^ BM loss ^¶^: −0.4 ± 0.2%	
			17.2 ± 1.9 °C 56.2 ± 11.8%	Court/field training 114 ± 23 min	330 ± 156 mL∙h^−1^	Sweat rate: 687 ± 114 mL∙h^−1^ BM loss ^¶^: −1.0 ± 0.4%	
			17.0 ± 1.3 °C 58.1 ± 15.6%	Competition 81 ± 7 min	601 ± 167 mL∙h^−1^	Sweat rate: 976 ± 254 mL∙h^−1^ BM loss ^¶^: −0.7 ± 0.5%	
			**SUMMER**	**SUMMER**	**SUMMER**	**SUMMER**	
			21.4 ± 0.4 °C 48.6 ± 3.5%	Weight training session -	38 ± 62 mL∙h^−1^	Sweat rate: 389 ± 121 mL∙h^−1^ BM loss ^¶^: −0.3 ± 0.4%	
			25.1 ± 0.9 °C 42.8 ± 6.8%	Court/field training 114 ± 7 min	413 ± 162 mL∙h^−1^	Sweat rate:1371 ± 235 mL∙h^−1^ BM loss ^¶^: −1.0 ± 0.5%	
			25.6 ± 1.5 °C 59.6 ± 7.5%	Competition 93 ± 2 min	599 ± 170 mL∙h^−1^	Sweat rate: 1601 ± 371 mL∙h^−1^ BM loss ^¶^: −0.9 ± 0.7%	
Heishman et al. 2021 [46]	M/15 20.4 ± 1.7	95.1 ± 7.4	n/a n/a	n/a	n/a	YEAR 1 **PRE-SEASON** USG: 1.020 ± 0.009 Incidence of EUH/DEH/significant DEH ^#^: 44.0/55.5/0.5%	Pre-season and competitive season; 2 consecutive years.
						**COMPETITIVE SEASON** USG: 1.022 ± 0.009 Incidence of EUH/DEH/significant DEH ^#^: 38.5/60.7/0.5% Playing time ≤ 15 min–USG: 1.021 ± 0.002 Playing time > 15 min—USG: 1.021 ± 0.006	
	M/16 18.9 ± 4.9	94.7 ± 9.7	n/a n/a	n/a	n/a	YEAR 2 **PRE-SEASON** USG: 1.019 ± 0.001 Incidence of EUH/DEH ^#^: 42.9 / 57.1 / 0.0%	
						**COMPETITIVE SEASON** USG: 1.021 ± 0.004 Incidence of EUH/DEH/significant DEH ^#^: 31.0/65.7/3.3% Playing time ≤ 15 min—USG: 1.022 ± 0.0012 Playing time > 15 min—USG: 1.022 ± 0.001	
Logan-Sprenger and McNaughton 2020 [47]	F/11 18–41	65.9 ± 16.1	22.1 ± 1.2 °C 55 ± 2%	18.27 ± 11.08 min	n/a	**WHOLE SAMPLE (*n* = 11)** (mean ± SD calculated based on raw data from original paper) Pre-game USG: 1.014 ± 0.006 BM loss: −0.5 ± 0.4% Δ in core temperature (T_c_): 1.0 ± 0.6 °C (*n* = 10) Highest T_c_: 38.6 ± 0.6°C (*n* = 10) Δ in skin temperature (T_sk_): 6.1 ± 1.5°C (*n* = 10) Incidence of DEH ^†^: 9%	Testing during a four-game series over four consecutive nights with the same game start time; each player was tested twice in the four-day period
		55.9 ± 6.9		20.88 ± 10.52 min	n/a	**SPINAL CORD INJURED GROUP (*n* = 7)** (mean ± SD calculated based on raw data from the original paper) Pre-game USG: 1.016 ± 0.005 BM loss: −0.4 ± 0.5% Δ in T_c_: 1.0 ± 0.5 °C (*n* = 6) Highest T_c_: 38.6 ± 0.5 °C (*n* = 6) Δ in T_sk_: 5.6 ± 1.3 °C (*n* = 6)	
		83.4 ± 11.4		13.68 ± 12.00 min	n/a	**NON-SPINAL CORD INJURED GROUP (*n* = 4)** (mean ± SD calculated based on raw data from the original paper) Pre-game USG: 1.011 ± 0.005 BM loss: −0.6 ± 0.3% Δ in T_c_: 0.9 ± 0.8 °C Highest T_c_: 38.5 ± 0.8 °C Δ in T_sk_: 6.8 ± 1.7 °C	
Osterberg et al. 2009 [48]	M/29 n/a	99 ± 18 (76–140)	20–22 °C 18–22%	Game - Game - Game 21.0 ± 8.0 min	**GAME I** 1.1 ± 0.7 L **GAME II** 1.0 ± 0.5 L **AVERAGE** 1.0 ± 0.6 (0.1–2.9) L	**GAME I** Pre-game USG: 1.020 ± 0.006 Sweat loss: −1.9 ± 0.7 L BM loss ^¥^: −1.2 ± 0.5% **GAME II** Pre-game USG: 1.019 ± 0.008 Sweat loss: −2.4 ± 0.9 L BM loss ^¥^: −1.6 ± 0.7% **AVERAGE** Incidence of DEH (USG > 1.020): 52% Sweat loss: −2.2 ± 0.8 (1.0–4.6) L BM loss^¥^: −1.4 ± 0.6 (0.5–3.2)% Sweat Na concentration: 41.6 ± 11.5 (21.3–58.1) mEq∙L^−1^ Total Na loss: −82.2 ± 38.2 (33.2–161.4) mEq NaCl loss: −4.8 ± 2.3 (1.9–9.5) g Na replacement: 16.6 ± 14.6 (0–49.7)% Sweat K concentration: 4.9 ± 0.7 (3.1–5.8) mEq∙L^−1^ Total K loss: −9.7 ± 2.7 (5.7–14.3) mEq	Athletes competed in 5 to 7 games throughout 9 to 10 days; measurements were taken from each player on 2 occasions, from 2 to 4 days apart
Schröder et al. 2004 [32]	M/50 25.1 ± 4.0	93.0 ± 11.0	n/a n/a	Training - Competition -	646 ± 352 mL∙h^−1 §^ 882 ± 486 mL∙h^−1 §^ Total daily intake: 3126 ± 1226 mL	-	-
Taim et al. 2021 [49] ^§^	M/18 23.1 ± 1.3	76.5 ± 12.1	n/a	-	-	USG: 1.018 ± 0.008 Incidence of EUH: 44.4% (USG ≤ 1.020) or 77.8% (USG ≤ 1.025) Incidence of DEH: 55.6% (USG > 1.020) or 22.2% (USG > 1.025)	-
Thigpen et al. 2014 [50]	M/11 21 ± 1	85.4 ± 7.6	22.5 ± 0.1 °C n/a	**Morning conditioning practices** 45.0 min	523 ± 250 mL	Sweat loss: −969 ± 250 mL Sweat rate: 1263 ± 326 mL∙h^−1^ BM loss: −1.1 ± 0.3%	-
			19.6 ± 2.5 °C n/a	**Afternoon sport-specific practices** 170.0 min	1535 ± 571 mL	Sweat loss: −2471 ± 495 mL Sweat rate: 872 ± 175 mL∙h^−1^ BM loss: −2.9 ± 0.6% Pre-practice USG: 1.026 ± 0.004 Incidence of minimal/significant/serious DEH: 18/68/14% *	
	F/11 19 ± 1	75.3 ± 10.1	23.9 ± 1.0 °C n/a	**Morning conditioning practices** 95.0 min	744 ± 230 mL	Sweat loss: −1112 ± 271 mL Sweat rate: 702 ± 171 mL∙h^−1^ BM loss: −1.5 ± 0.3%	
			23.7 ± 0.8 °C n/a	**Afternoon sport-specific practices** 170.0 min	1101 ± 411 mL	Sweat loss: −1910 ± 441 mL Sweat rate: 674 ± 156 mL∙h^−1^ BM loss: −2.5 ± 0.4% Pre-practice USG: 1.022 ± 0.008 (*n* = 10) Incidence of minimal/significant/serious DEH: 25 / 55 / 20% *	
Vukasinović-Vesić et al. 2015 [51]	M/96 19.0 ± 0.79 (16–20)	90.6 ± 12.4 (62–144)	30 ± 2 °C (27.2–32.5 °C) 55 ± 4% (48–58%)	Game 18.8 ± 10.5 min (0.15–40 min)	Fluid intake 1.87 ± 0.82 L (0.38–3.98 L) Fluid intake rate 1.79 ± 0.8 L∙h^−1^ (0.4–19 L∙h^−1^)	**PRE-GAME** USG: 1024 ± 0.6; UC: 5.67 ± 1.12; UO: 883 ± 229 mOsm Incidence of DEH based on USG: 80% (> 1.020); UC (>4): 95%; UO: 75% (>700 mOsm) **POST-GAME** USG: 1026 ± 6; UC: 5.97 ± 1.37 UO: 852 ± 228 mOsm Incidence of DEH based on USG: 85%; UC: 95%; UO: 75% Sweat rate: 2.7 ± 0.9 (0.23–5.54) L∙h^−1^ BM loss: −0.9 ± 0.7 (−1.0–2.9) kg Level of DEH: 0.99 ± 0.7 (−1.25–2.95)%	Evaluation during the FIBA Europe U20 Championship

Abbreviations: BM, body mass; DEH, dehydration; EUH, euhydration; F, female; M, male; T_c_, core temperature; T_sk_, skin temperature; UC, urine colour; UO, urine osmolality; USG, urine specific gravity. n/a—data not available. ^§^ Baseline data extracted from papers reporting on interventional studies [40,42,49]. ^†^ USG ≥ 1.020 adopted as hypohydration [40]. ^¥^ BM loss defined as pre- vs. post-exercise changes in BM [40,41,43,44,48]. ^ǂ^ USG < 1.020 adopted as euhydration [41]. ^¶^ BM loss calculated using the following equation: BM loss (%) = [(BM change − urine output)/initial BM] × 100 [45]. ^#^ The following classification was applied: USG ≤ 1.020 ‘euhydrated’, USG 1.021–1.030 ‘hypohydrated’, USG > 1.030 significantly ‘hypohydrated’ [46]. * The following classification was applied: USG ≤ 1.020 ‘minimal dehydration’, USG 1.021–1.030 ‘significant dehydration’, USG > 1.030 ‘serious dehydration’ [50].

**Table 3:** Characteristics of interventional studies investigating dietary interventions related to dehydration and/or hydration strategies in basketball players.

Reference	Study Design	Gender/*n*	Age (years)	Intervention	Experimental Procedures ^†^	Outcomes
Baker et al. 2007 [52]	Six-arm randomized cross-over placebo-controlled trial (double-blind with respect to euhydration [EUH] trials)	M/11	21 ± 3 (17–28)	(1) EUH with lemon/lime-flavored carbohydrate–electrolyte solution (CES; 6% carbohydrate [CHO] and 18.0 mM NaCl)—**EUH-CES** (2) EUH with a **placebo** (PLA; lemon/lime-flavored water and 18.0 mM NaCl)—**EUH-PLA** (3) **1% dehydration (DEH)** (4) **2% DEH** (5) **3% DEH** (6) **4% DEH** Results presented as means of two implemented EUH conditions (EUH-CES and EUH-PLA) and four distinct DEH conditions (1% DEH—4% DEH)	(1) BASELINE evaluation (blood sampling, blood pressure [BP], heart rate [HR], core body temperature [Tc], Test of Variables of Attention [TOVA], ratings of fatigue) (2) Procedure of EUH/DEH obtaining via EXERCISE/HEAT exposure during interval-walking protocol (9 bouts × 15 min walking at 50% maximal oxygen uptake [VO_2_max] with 5-min rest between bouts, temperature: 40°C, relative humidity: 20%) (3) POST-EXERCISE/HEAT evaluation (4) Recovery (50 min + 20 min travel) (5) Basketball drill test (4 bouts × 15 min of drills with a 5-min break between quarters [QR] and a 10-min break at halftime) (6) Ratings of fatigue at the HALFTIME drill test (7) Post-drill test evaluation—END	**PHYSIOLOGICAL VARIABLES** ***Tc*** POST-EXERCISE/HEAT: ↑ DEH vs. EUH END: ↔ DEH vs. EUH ***Δ Plasma volume (PV)*** POST-EXERCISE/HEAT: ↓ DEH vs. EUH ***Serum glucose*** POST-EXERCISE/HEAT: *↓* DEH vs. EUH, ↑ EUH-CES vs. EUH-PLA, ↑ EUH-CES vs. DEH **RATINGS OF FATIGUE** ***Lightheadedness, hotness*** POST-EXERCISE/HEAT, HALFTIME, END: ↑ DEH vs. EUH ***Total body fatigue*** POST-EXERCISE/HEAT, HALFTIME: ↑ DEH vs. EUH END: ↔ DEH vs. EUH **VIGILANCE—TOVA—TARGET-INFREQUENT CONDITION** ***Sensitivity*** (Δ from BASELINE) POST-EXERCISE/HEAT: ↔ DEH vs. EUH END: ↓ DEH vs. EUH ***Response time, omission errors*** (Δ from BASELINE) POST-EXERCISE/HEAT: ↔ DEH vs. EUH END: ↑ DEH vs. EUH ***Commission errors*** (Δ from BASELINE) POST-EXERCISE/HEAT, END: ↔ DEH vs. EUH VIGILANCE—TOVA—TARGET-FREQUENT CONDITION ***Sensitivity*** (Δ from BASELINE) POST-EXERCISE/HEAT: ↔ DEH vs. EUH END: ↓ DEH vs. EUH ***Response time*** (Δ from BASELINE) POST-EXERCISE/HEAT: ↔ DEH vs. EUH END: ↑ DEH vs. EUH ***Omission and commission errors*** (Δ from BASELINE) POST-EXERCISE/HEAT: ↑ DEH vs. EUH END: ↔ DEH vs. EUH
Baker et al. 2007 [42]	Six-arm randomized cross-over placebo-controlled trial (double-blind concerning EUH trials)	M/17	21.1 ± 2.4 (17—28)	(1) EUH with lemon/lime-flavored CHO–electrolyte solution CES; 6% CHO and 18.0 mM NaCl)—**EUH-CES** (2) EUH with a **PLA** (lemon/lime-flavored water and 18.0 mM NaCl)—**EUH-PLA** (3) **1% DEH** (4) **2% DEH** (5) **3% DEH** (6) **4% DEH** Results presented for EUH-PLA and separately for four distinct DEH conditions EUH-CES excluded from the presentation due to a lack of differences between EUH conditions and for simplification	(1) BASELINE evaluation (blood sampling, BP, HR, Tc) (2) Procedure of EUH/DEH obtaining via EXERCISE/HEAT exposure during interval-walking protocol (9 bouts × 15 min walking at 50% VO_2_max with 5-min rest between bouts, temperature: 40 °C, relative humidity: 20%) (3) POST-EXERCISE/HEAT evaluation (same as at baseline and additionally ratings of fatigue, rate of perceived exertion [RPE]) (4) Recovery (70 min) (5) RECOVERY evaluation (same as at POST-EXERCISE/HEAT) (6) Basketball drill test (4 bouts × 15 min of drills with a 5-min break between QRs and a 10-min break at HALFTIME) (7) HALFTIME drill test (after 2nd QR) evaluation (same as at POST-EXERCISE/HEAT) (8) Post-drill test (after 4th QR) evaluation (same as at POST-EXERCISE/HEAT)—END	**PHYSIOLOGICAL VARIABLES** ***HR*** POST-EXERCISE/HEAT: ↑ 1–4% DEH vs. EUH RECOVERY: ↔ 1–3% DEH vs. EUH, ↑ 4% DEH vs. EUH HALFTIME: ↔ 1–4% DEH vs. EUH END: ↔ 1, 3, 4% DEH vs. EUH, ↑ 2% DEH vs. EUH ***Tc*** POST-EXERCISE/HEAT: ↔1% DEH vs. EUH, ↑ 2–4% DEH vs. EUH RECOVERY, HALFTIME: ↔ 1–3% DEH vs. EUH, ↑ 4% DEH vs. EUH END: ↔ 1–4% DEH vs. EUH ***Mean arterial pressure (MAP)*** POST-EXERCISE/HEAT: ↔ 1–2% DEH vs. EUH, ↓ 3–4% DEH vs. EUH RECOVERY: ↔1–4% DEH vs. EUH **BLOOD VARIABLES** (at the END of the whole protocol) ***Glucose***: ↔ 1–4% DEH vs. EUH ***Sodium***: ↔ 1% DEH vs. EUH; ↑ 2–4% DEH vs. EUH, ***Osmolality, Δ PV*** (change from BASELINE): ↓ 1–4% DEH vs.EUH ***Protein***: ↔ 1–2% DEH vs. EUH, ↑ 3–4% DEH vs. EUH **RATINGS OF FATIGUE** ***Lightheadedness, leg fatigue*** POST-EXERCISE/HEAT, END: ↔ 1–2% DEH vs. EUH, ↑ 3–4% DEH vs. EUH ***Windedness, hotness, muscle cramping*** POST-EXERCISE/HEAT: ↔ 1–2% DEH vs. EUH, ↑ 3–4% DEH vs. EUH END: ↔ 1–4% DEH vs. EUH ***Upper and total body fatigue*** POST-EXERCISE/HEAT: ↔ 1–2% DEH vs. EUH, ↑ 3–4% DEH vs. EUH END: ↔ 1–3% DEH vs. EUH, ↑ 4% DEH vs. EUH ***Side stitch/ache*** POST-EXERCISE/HEAT, END: ↔ 1–4% DEH vs. EUH **BASKETBALL PERFORMANCE** *Comparison between EUH conditions* No advantage of EUH-CES condition over EUH-PLA concerning basketball performance *Comparisons between EUH and levels of DEH* ***Baseline jump shots***: ↔ 1–3% DEH vs. EUH, ↓ 4% DEH vs. EUH ***Lay-up shots***: ↔ 1–2% DEH vs. EUH, ↓ 3–4% DEH vs. EUH ***Foul line jump shots***: ↔ 1–4% DEH vs. EUH ***Total shots on the move***: ↔1% DEH vs. EUH, ↓ 2–4% DEH vs. EUH, ***20 court widths sprint***: ↔ 1–2% DEH vs. EUH, ↑ 3–4% DEH vs. EUH ***Ladder suicide sprint***: ↑ 1–4% DEH vs. EUH ***Total sprint time, all timed drills***: ↔ 1% DEH vs. EUH, ↑ 2–4% DEH vs. EUH ***All shots***: ↔ 1% DEH vs. EUH, ↓ 2–4% DEH vs. EUH ***RPE*** POST-EXERCISE/HEAT: ↔ 1–2% DEH vs. EUH, ↑ 3–4% DEH vs. EUH HALFTIME, END: ↔ 1–4% DEH vs. EUH
Carvalho et al. 2011 [53]	Three-arm randomized cross-over trial	M/12	14.8 ± 0.45 (14—15)	Three training sessions under distinct hydration conditions: (1) No fluid (**NF**) ingestion (2) Ad libitum ingestion of water (**W**, 3.8 mg∙L^−1^ Na) (3) Ad libitum ingestion of **CES** (7.2% sugar, 0.8% maltodextrin, 510 mg∙L^−1^ Na)	(1) Baseline evaluation (BM) (2) 90-min training (3) 30-min evaluation of basketball performance drills (4) Post-exercise evaluation (urine sampling, RPE, beverage acceptability)	***BM loss***: ↑ NF (−2.46 ± 0.87%) vs. W (−1.08 ± 0.67%) vs. CES (−0.65 ± 0.62%), ↑ W vs. CES ***Sweat rate, urine colour (UC)***: ↔ between conditions ***Fluid intake, beverage acceptability***: ↔ W vs. CES ***RPE (6—20)***: ↑ NF vs. W, ↑ NF vs. CSB ***Basketball performance (2- and 3-point shooting, free-throw shooting, suicide sprints, defensive zigzag)***: ↔ between conditions
Dougherty et al. 2006 [54]	Three-arm randomized placebo-controlled double-blind cross-over trial	M/15	13.5 ± 1.3 (12—15)	(1) 2% DEH—**DEH** (2) EUH with CES (6% CHO and 18.0 mmol∙L^−1^ Na)—**EUH-CES** (3) EUH with a flavored water PLA (0% CHO and 18.0 mmol∙L^−1^ Na)–**EUH-PLA**	(1) BASELINE evaluation (urine sampling, BM, BP, HR, Tc) (2) Procedure of EUH/DEH obtaining via EXERCISE/HEAT exposure during interval-exercise protocol (6 bouts of 15-min treadmill/cycle ergometer exercise at 50%VO_2_ max with 5-min rests; temperature: 35 °C, relative humidity: 20%) (3) POST-EXERCISE/HEAT evaluation (urine sampling, BM, BP, HR, Tc, RPE, fluid intake, ratings of fatigue) (4) 1 h recovery (5) RECOVERY evaluation (urine sampling, BM, BP, HR, Tc, fluid intake) (6) Basketball drill test (4QRs × 12 min of drills with 10-min break at HALFTIME) (7) HALFTIME drill test (after 2nd QR) evaluation (same as at POST-EXERCISE/HEAT) (8) Post-drill test (after 4th QR) evaluation (POST-EXERCISE/HEAT)—END	**PHYSIOLOGICAL VARIABLES** ***HR*** POST-EXERCISE/HEAT: ↑ DEH vs. EUH-PLA, ↑ DEH vs. EUH-CES RECOVERY, HALFTIME: ↔ DEH vs. EUH-PLA, ↔ DEH vs. EUH-CES END: ↑ DEH vs. EUH-PLA, ↔DEH vs. EUH-CES ***T_c_*** POST-EXERCISE/HEAT: ↑ DEH vs. EUH-PLA, ↑ DEH vs. EUH-CES RECOVERY, HALFTIME: ↔ DEH vs. EUH-PLA, ↔ DEH vs. EUH-CES END: ↑ DEH vs. EUH-PLA, ↑ DEH vs. EUH-CES ***MAP*** POST-EXERCISE/HEAT, RECOVERY: ↔ DEH vs. EUH-PLA, ↔ DEH vs. EUH-CES ***RPE*** POST-EXERCISE/HEAT: ↑ DEH vs. EUH-PLA, ↑ DEH vs. EUH-CES HALFTIME, END: ↔ DEH vs. EUH-PLA, ↔ DEH vs. EUH-CES **RATINGS OF FATIGUE** ***Lightheadedness*** POST-EXERCISE/HEAT: ↑ DEH vs. EUH-PLA, ↑ DEH vs. EUH-CES HALFTIME, END: ↔ DEH vs. EUH-PLA, ↑ DEH vs. EUH-CES ***Windedness, hotness*** POST-EXERCISE/HEAT: ↔ DEH vs. EUH-PLA, ↑ DEH vs. EUH-CES HALFTIME, END: ↔ DEH vs. EUH-PLA; ↔ DEH vs. EUH-CES ***Upper-body fatigue*** POST-EXERCISE/HEAT: ↔ DEH vs. EUH-PLA, ↔ DEH vs. EUH-CES HALFTIME: ↑ DEH vs. EUH-PLA, ↑ DEH vs. EUH-CES END: ↔ DEH vs. EUH-PLA, ↑ DEH vs. EUH-CES ***Total body fatigue*** POST-EXERCISE/HEAT, HALFTIME: ↔ DEH vs. EUH-PLA, ↔ DEH vs. EUH-CES END: ↔ DEH vs. EUH-PLA, ↑ DEH vs. EUH-CES ***Side stitch/ache, muscle cramping, leg fatigue*** POST-EXERCISE/HEAT, HALFTIME, END: ↔ DEH vs. EUH-PLA, ↔ DEH vs. EUH-CES BASKETBALL PERFORMANCE ***Around the world shots, free throws: ↔*** DEH vs. EUH-PLA; ↓ DEH vs. EUH-CES ***3-point shots***: ↓ DEH vs. EUH-PLA, ↓ DEH vs. EUH-CES ***Combined shooting***: ↓ DEH vs. EUH-PLA, ↓ DEH vs. EUH-CES, ↑ EUH-CES vs. EUH PLA ***Short-range (layups) shooting***: ↔ between conditions ***10 widths sprinting***: ↑ DEH vs. EUH-PLA, ↑ DEH vs. EUH-CES, ↔ EUH-CES vs. EUH-PLA ***Suicides sprinting, average and total sprints’ times***: ↑ DEH vs. EUH-PLA, ↑ DEH vs. EUH-CES, ↓ EUH-CES vs. EUH-PLA ***Lateral movement drills—zigzags, lane slides, average and total lateral movements’ times***: ↑ DEH vs. EUH-PLA, ↑ DEH vs. EUH-CES, ↔ EUH-CES vs. EUH-PLA ***Individual full-court combination times***: ↔ between conditions ***Individual key combination times: ↑*** DEH vs. EUH-PLA, ↑ DEH vs. EUH-CES ***Average and total defensive drills’ times***: ↔ DEH vs. EUH-PLA, ↑ DEH vs. EUH-CES ***Time to complete 10 vertical jumps (VJ) and maximum VJ height***: ↔ between conditions
Hoffman et al. 1995 [55]	Two-arm balanced cross-over design	M/10	17.3 ± 0.9	Two stimulated ‘2 × 2 full-court’ basketball games under distinct hydration conditions: (1) drinking water permitted—**Wa** (2) restriction from any fluid consumption—**NWa** Ambient temperature: 20.8 ± 0.9 °C, relative humidity: 0.64 ± 0.05%	(1) 15-min standardized warm-up (2) PRE evaluation of dynamic strength of the lower limb and anaerobic power (3) Game—1st half (field goal attempts [FGA] and free throw attempts [FTA] evaluation, BM measurements [at 7, 14 and 20 min]) (4) HALF-game evaluation of dynamic strength of the lower limb and anaerobic power (5) 15-min break (6) Game—2nd half (FGA, FTA, BM at 7, 14, and 20 min)—POST	***BM loss at NWa condition (compared to PRE evaluation)*** HALF: −1.1 ± 0.4% POST: −1.9 ± 0.4% **DYNAMIC STRENGTH OF THE LOWER EXTREMITY** ***Squat jump and countermovement jump heights***: ↔ Wa vs. NWa at any time point **ANAEROBIC POWER AND CAPACITY** ***Anaerobic power, number of jumps, average jump height***: ↔ Wa vs. NWa at any time point **BASKETBALL PERFORMANCE** ***FGA, FTA***: ↔ Wa vs. NWa
Hoffman et al. 2012 [56]	Four-arm double-blind cross-over design	F/10	21.2 ± 1.6	Four 40-min basketball games under distinct hydration conditions: (1) no drinking allowed—**DHY** (2) water allowed—**W** (3) water combined with L-alanyl-glutamine—1 g per 500 mL—**AG1** (4) water combined with L-alanyl-glutamine—2 g per 500 mL—**AG2** At conditions 2–3 fluid intake was adjusted to pre-evaluated fluid loss (during DHY condition) Environmental conditions Temperature: 22.6 ± 0.19 °C Relative humidity: 50.9 ± 3.1%	(1) 10-min dynamic typical warm-up (2) PRE-game testing battery (power [countermovement jump, CMJ], reaction [lower-body and hand-eye reaction time], basketball shooting circuit—5 shoots from 6 different locations on the court) (3) Game (HR, game load assessment) (4) POST-game testing battery: CMJ, reaction, and basketball shooting assessment	***BM loss at DHY condition***: ~−2.3% (−1.72 ± 0.42 kg) ***Fluid intake***: ↔ W, AG1, AG2 BASKETBALL PERFORMANCE *Δ* POST-PRE ***in field goal shooting performance***: ↓ DHY vs. AG1, ↓ W vs. AG1, ↔ between remaining conditions REACTION *Δ* POST-PRE ***in lower-body reaction (number of successful attempts)***: ↓ DHY vs. W, ↓ DHY vs. AG1, ↓ DHY vs. AG2, ↔ between remaining conditions *Δ* POST-PRE ***in visual reaction time***: ↓ DHY vs. AG1, ↔ between remaining study conditions *Δ* POST-PRE ***in motor reaction time***: ↔ between conditions *Δ* POST-PRE ***in physical reaction time***: ↓ DHY vs. AG1, ↔ between remaining study conditions POWER *Δ* POST-PRE ***in peak and mean VJ power***: ↔ between conditions ***Player load***: ↑ AG2 vs. DHY, ↔ between remaining study conditions ***HR***: ↔ between study conditions
Louis et al. 2018 [57]	Two-arm randomized cross-over trial	M/9	16.2 ± 0.7	Two basketball trails under distinct hydration conditions: (1) **EUH** (2) **DEH** (~−2% BM)	(1) EUH/DEH obtaining procedure (60 min of low-intensity [90 ± 10 W] in an environmental chamber at 39 °C (2) 10-min rest (3) Habitual warm-up (4) Three-point shots test (success rate, shooting technique analysis, RPE)	***Success rate and number of throws (per minute) in three-point shots test***: ↔ DEH vs. EUH *RPE*: ↑ DEH vs. EUH ***Variables of body kinematics and ball release during three-point shots test***: ↔ DEH vs. EUH
Minehan et al. 2002 [58]	Three-arm randomized cross-over design	M/8 F/7	-	Nine training sessions under three distinct hydration conditions (3 trainings per condition): (1) **WATER** (2) **CES** (6.8% CHO, 1130 kJ∙L^−1^, 18.7 mmol∙L^−1^ Na, 2 mmol∙L^−1^ K) (3) low energy-electrolyte beverage (**LKEB**; 1% CHO, 170 kJ L^−1^, 18.7 mmol L^−1^ Na, 3 mmol L^−1^ K)	Ad libitum intake of **WATER**/**CES**/**LKEB** during nine trainings characterized by similar time and structure and undertaken in comparable environmental conditions (temperature 17.8 ± 0.9 °C, relative humidity 40.4 ± 8.1%). Evaluation of fluid intake, sweat loss, fluid balance	***Fluid intake, sweat loss***: ↔ between any fluids in M and F ***Fluid balance in M***: ↑ CES vs. WATER, ↔ LKEB vs. WATER, ↔ CES vs. LKEB ***Fluid balance in F***: ↑ CES vs. WATER, ↑ LKEB vs. WATER, ↔ CES vs. LKEB
Taim et al. 2021 [49]	Parallel group randomized between-subject design	M/18	23.1 ± 1.3	3 × 3 small-sided basketball game with participants divided into two groups consuming: (1) colourless, flavoured water (without CHO; sweetened with acesulfame K and sucralose, and containing negligible amounts of Na [less than 10 mg per 250 mL])—**FW** (2) plain water—**PW** Environmental conditions Temperature: 31.7 ± 0.5 °C Relative humidity: 62 ± 4%	(1) PRE-GAME evaluation (urine sampling, BM, fluid palatability, RPE, thirst) (2) standardized warm-up (3) 40-min GAME (HR, RPE, and thirst evaluation) (4) POST-GAME evaluation (BM, urine sampling)	**PALATABILITY RATINGS** ***Hedonic rating, sweetness, saltiness, sourness fluid***: ↑ FW vs. PW **HYDRATION STATE** ***Fluid consumption, sweat rate***: ↔ FW vs. PW **BM loss**: ↔ FW (−0.941 ± 0.524%) vs. PW (−0.534 ± 0.376%) ***HR***: ↔ FW vs. PW ***RPE***: ↔ FW vs. PW **BASKETBALL PERFORMANCE** ***2-point and 3-point field-goal percentage, number of assists and defensive rebounds***: ↔ FW vs. PW

Abbreviations: AG1, water combined with L-alanyl-glutamine—1 g per 500 mL condition; AG2, water combined with L-alanyl-glutamine—2 g per 500 mL condition; BM, body mass; BP, blood pressure; CES, carbohydrate-electrolyte solution; CHO, carbohydrate; CMJ, countermovement jump; DEH, dehydration; DHY, no drinking allowed condition; EUH, euhydration; F, female; FGA, field goal attempts; FTA, free throw attempts; FW, flavoured water condition; HR, heart rate; LKEB, low energy-electrolyte beverage; M, male; MAP, mean arterial pressure; NF, no fluid intake condition; NWa, restriction from any fluid consumption condition; PLA, placebo; PV, plasma volume; PW, plain water condition; QR, quarter; RPE, ratings or perceived exertion; T_c_, core temperature; TOVA, Test of Variables of Attention; UC, urine colour; VJ, vertical jump; VO_2_max, maximal oxygen uptake; W, water intake condition; Wa, drinking water permitted condition. ↑ significantly higher than comparator; ↔ no difference between comparators; ↓ significantly lower than comparator. ^†^ The UNDERLINED UPPER-CASE SCRIPTS in the descriptions of experimental procedures refer to the time point when evaluation measurements were taken.

**Table 4:** Characteristics of interventional studies investigating dietary interventions related to macronutrient intake manipulation in basketball players.

Reference	Study Design	Gender/*n*	Age (years)	Dietary Intervention	Experimental Procedures ^†^	Outcomes
Afman et al. 2014 [59]	Two-arm randomized cross-over counterbalanced placebo-controlled trial	M/10	20 ± 1	Single acute ingestion of 75 g carbohydrate (CHO) as sucrose dissolved in 500 mL of sugar-free, orange-flavored, artificially sweetened beverage (**CHO-SOL**) 45 min before exercise **vs.** ingestion of volume, taste- and colour-matched placebo (**PLA**)	(1) BASELINE body mass (BM) measurement and blood sampling (glucose, lactate analyses) (2) **CHO-SOL/PLA** ingestion (3) 45-min rest (4) PRE-EXERCISE blood sampling (5) Basketball stimulation test—a modified version of Loughborough Intermittent Shuttle Test (LIST)—blood sampling and rate of perceived exertion (RPE) at 5 min of the 1st quarter (QR) and after the 1st QR, 2nd QR, 3rd QR, and 4th QR	**BLOOD VARIABLES** ***Glucose*** BASELINE: ↔ CHO-SOL vs. PLA PRE-EXERCISE: ↑ CHO-SOL vs. PLA at 5 min of 1st QR, 1st QR: ↓ CHO-SOL vs. PLA 2nd, 3rd, 4th QR: ↔ CHO-SOL vs. PLA ***Lactate***: ↔ CHO-SOL vs. PLA at any time point **BASKETBALL PERFORMANCE (LIST)** ***Layup shooting*** 1st QR and overall mean: ↓ CHO-SOL vs. PLA 2nd, 3rd, 4th QR: ↔ CHO-SOL vs. PLA ***20 m sprint time*** 1st QR: ↑ CHO-SOL vs. PLA 2nd, 3rd QR and overall mean: ↔ CHO-SOL vs. PLA 4th QR: ↓ CHO-SOL vs. PLA
Daniel et al. 2019 [60]	Two-arm randomized cross-over trial	M/9	18.0 ± 0.7	Single ingestion of high-glycemic index (**HGI**; glycemic index [GI]) 71.8—74.9) ***versus*** low-glycemic index (**LGI**; GI 47.9–49.5) dinner and evening snacks across two consecutive days during competition Ad libitum intake of food during remaining meals	**The day before competition:** (1) 3-h fast (2) BEFORE DINNER (BD) evaluation of sleepiness (Epworth Sleepiness Scale [ESS], visual analogue scale [VAS]) and satiety (VAS); saliva sampling (melatonin, cortisol) (3) **HGI/LGI** dinner at 19:00 (4) blood sampling before (BD), 30 and 60 min after the start of **HGI/LGI** (5) AFTER DINNER (AD) evaluation of sleepiness and satiety (6) ~9:30 p.m. **HGI/LGI** evening snack (7) BEFORE SLEEP (BS) saliva sampling (8) Actigraph monitoring of sleep pattern **Day of competition:** (9) AFTER AWAKING (AA) evaluation of sleepiness, saliva sampling (free awaking between 7:00 and 8:00 a.m.) (10) BEFORE BREAKFAST (BB) evaluation of sleepiness and satiety, saliva sampling (11) Game (between 9:00 and 12:00 a.m.)	**ENERGY AND MACRONUTRIENT INTAKE** ***CHO (g∙kg^−1^)***: ↑ HGI vs. LGI ***Energy, protein (PRO), fat, CHO as % of energy intake (EI)***: ↔ HGI vs. LGI **GLYCEMIC RESPONSE TO HGI/LGI DINNER** ***Area under the curve***: ↑ HGI vs. LGI **SATIETY** at BD, AD, BB: ↔ HGI vs. LGI **SALIVA HORMONES** ***Melatonin, cortisol*** at BD, BS, AA, BB: ↔ HGI vs. LGI **SLEEPINESS** ***Based on VAS*** at BD, AD, BB: ↔ HGI vs. LGI ***Based on ESS*** at BD, BB: ↔ HGI vs. LGI ***Sleep pattern (nocturnal and daytime sleep time, sleep latency, sleep efficiency, wake after sleep onset)***: ↔ HGI vs. LGI
Gentle et al. 2014 [61]	Two-arm randomized cross-over trial	M/10	22 ± 2	Single ingestion of CHO (1g_CHO_∙kg_BM_^−1^) in conjunction with PRO (1g_PRO_∙kg_BM_^−1^; **CHO-PRO**) **vs.** CHO alone (2g_CHO_∙kg_BM_^−1^, **CHO**) 90 min before 87-min exercise protocol	(1) BASELINE fasting blood and saliva sampling (2) Ingestion of **CHO-PRO/CHO meals** (3) Anthropometric measurements (4) Heart rate (HR) recording during the entire protocol (5) PRE-EXERCISE urine and blood sampling, evaluation of gastrointestinal upset (GIU) and muscle soreness (MS) (6) Warm-up (7) Basketball performance protocol (4 QRs × 15 min with 15-min rest after 2nd QR) (8) DURING-EXERCISE (after 2nd QR) blood sampling, evaluation of RPE, MS, GIU (9) POST-EXERCISE blood, urine, and saliva sampling, evaluation of RPE, MS, GIU (10) 30-min post-exercise venous blood sampling (11) AFTER 24 h venous blood, urine, and saliva sampling, evaluation of RPE, MS, GIU	**PHYSIOLOGICAL VARIABLES** ***Mean*** and ***peak HR***: ↔ CHO-PRO vs. CHO **BLOOD VARIABLES** ***Lactate*** PRE-, DURING-, POST-EXERCISE: ↔ CHO-PRO vs. CHO ***Glucose*** PRE-EXERCISE: ↔ CHO-PRO vs. CHO DURING-, POST-EXERCISE: ↑ CHO-PRO vs. CHO ***Mean Δ in creatine kinase (CK) activity*** BASELINE to POST-EXERCISE: ↓ CHO-PRO vs. CHO BASELINE to AFTER 24 h, POST-EXERCISE to AFTER 24 h: ↔ CHO-PRO vs. CHO **SALIVA HORMONES CONCENTRATIONS** ***Cortisol*** BASELINE, AFTER 24 h: ↔ CHO-PRO vs. CHO POST-EXERCISE: ↑ CHO-PRO vs. CHO ***Testosterone***: ↔ CHO-PRO vs. CHO at any time point **BASKETBALL PERFORMANCE** ***Mean jump height, sprint time***: ↔ CHO-PRO vs. CHO at any time point ***Mean success rate for the first two free throw attempts***: ↑ CHO-PRO vs. CHO at 4th QR (no differences at any other time point) ***MS (upper, lower, and whole body):*** ↔ CHO-PRO vs. CHO at any time point **GASTROINTESTINAL UPSET** ***Increase in nausea and belching*** from BASELINE to DURING-EXERCISE: ↑ CHO-PRO vs. CHO ***Increase in nausea and stomach bloating*** from BASELINE to POST-EXERCISE: ↑ CHO-PRO vs. CHO **RPE** at 1st QR and 4th QR: ↑ CHO-PRO vs. CHO (no differences at any other time point)
Ghiasvand et al. 2010 [62]	Three-arm randomized double-blind placebo-controlled parallel group clinical trial	M/34	24 * (15–35)	6 weeks of supplementation with: (1) 2 g of eicosapentaenoic acid (EPA) + 400 IU vitamin E—**EPA + Vit E** (*n* = 8) **vs.** (2) 2 g **EPA + PLA** (*n* = 9) **vs.** (3) 400 IU **Vit E + PLA** (*n* = 9) **vs.** (4) **PLA + PLA** (*n* = 8)	Venous blood samples (for interleukin 2 [IL-2], tumor necrosis factor alfa [TNF-α], and malonylodialdehyde [MDA] concentration, catalase, and glutathione reductase activity) between 5:00 and 6:00 p.m., after intensive endurance exercising for 2 h, at the BASELINE and AFTER 6-week supplementation	BASELINE vs. AFTER comparisons ***TNF-α*** in **EPA + Vit E**: ***↓*** AFTER vs. BASELINE ***MDA*** in **EPA + PLA** and **Vit E + PLA**: ***↓*** AFTER vs. BASELINE ***Glutathione reductase activity*** in **EPA + Vit E** and **Vit E + PLA**: ***↓*** AFTER vs. BASELINE No differences in the remaining groups and/or remaining variables BETWEEN GROUPS COMPARISONS AFTER SUPPLEMENTATION ***IL-2***: ↑ **EPA + Vit E** vs. **EPA + PLA**; ↑ **EPA + Vit E** vs. **Vit E + PLA**; ↓ **EPA + Vit E** vs. **PLA + PLA** ***Glutathione reductase activity: ↓*** **EPA + PLA** vs. **Vit E + PLA** No between-group differences in the remaining groups and/or variables
Ho et al. 2018 [63]	Two-arm randomized, placebo-controlled counterbalanced cross-over trial	-/15	18–20	Single oral ingestion of 600 mL of **high-PRO** (36% PRO, 58% CHO, 6% FAT in total energy) ***versus*** **low-PRO** (12% PRO, 63% CHO, 25% FAT) isoenergetic drink (6.25 kcal∙kg^−1^) immediately after 1 h endurance cycling at 70% of maximal oxygen uptake (VO_2_max)	(1) PRE-exercise fasting blood sampling (2) 1 h endurance cycling at 70% VO_2_max (3) consumption of **high-PRO/low-PRO** drink (4) blood sampling immediately POST-ingestion of a drink and every 30 min until 2 h recovery completion (30, 60, 90, and 120 min) (5) Endurance time trial (TT) on the cycloergometer at 80% VO_2_max—simultaneous monitoring of cerebral hemodynamic response	***Glucose*** PRE, POST, 30 min: ↔ high-PRO vs. low-PRO 60, 90, 120 min: ↓ high-PRO vs. low-PRO ***Insulin*** PRE, POST, 60, 90, 120 min: ↔ high-PRO vs. low-PRO 30 min: ↑ high-PRO vs. low-PRO ***Time to exhaustion in cycling TT***: ↑ high-PRO vs. low-PRO ***Percent oxygen saturation in the frontal brain at 60, 120, 180, 240, and 300 s of cycling TT***: ↑ high-PRO vs. low-PRO ***Blood perfusion (total hemoglobin) to the brain during cycling at 60, 120, 180, 240, and 300 s of cycling TT***: ↓ high-PRO vs. low-PRO
Marques et al. 2015 [64]	Single-arm intervention	M/8	33.8 ± 8.3	30 days supplementation with 3 g of fish oil (1500 mg docosahexaenoic acid, 300 mg EPA, and 6 mg vitamin E)	Pre- (S0) and post-supplementation (S1) resting (REST) and after ACUTE EXERCISE blood sample analysis	**LIPID PROFILE** ***Total cholesterol*** S0: ↑ ACUTE EXERCISE vs. REST S1: ↔ ACUTE EXERCISE vs. REST ***LDL- and HDL-Chol, triglycerides***: no effect of supplementation or exercise **MUSCLE DAMAGE** ***Lactate dehydrogenase activity*** S0: ↑ ACUTE EXERCISE vs. REST(55.4% increase) S1: ↔ ACUTE EXERCISE vs. REST ***CK activity***: no effect of supplementation or exercise **INFLAMMATORY MEDIATORS** ***Interleukin 6 (IL-6), Interleukin 1ra (IL-1ra)*** S0: ↑ ACUTE EXERCISE vs. REST S1: ↔ ACUTE EXERCISE vs. REST ACUTE EXERCISE: ↓ S1 vs. S0 ***Interleukin 8 (IL-8)*** S0: ↔ ACUTE EXERCISE vs. REST S1: ↑ ACUTE EXERCISE vs. REST ***C-reactive protein, TNF-α, interleukin 1β (IL-1β), interleukin 4 (IL-4)***: no effect of supplementation or exercise **NEUTROPHIL FUNCTION AND DEATH** ***IL-6 production by lipopolysaccharide-stimulated neutrophils*** S0: ↓ ACUTE EXERCISE vs. REST S1: ↔ ACUTE EXERCISE vs. REST ***IL-8, TNF-α, IL-1ra, IL-1β, IL-4 production by unstimulated and stimulated neutrophils***: no effect of supplementation or exercise ***Pathogenic capacity of neutrophils***: S0: ↓ ACUTE EXERCISE vs. REST S1: ↔ ACUTE EXERCISE vs. REST REST: ↓ S1 vs. S0 ACUTE EXERCISE: ↔ S1 vs. S0 ***Percentage of cells with loss of membrane integrity:*** S0: ↑ ACUTE EXERCISE vs. REST S1: ↔ ACUTE EXERCISE vs. REST REST: ↑ S1 vs. S0 ACUTE EXERCISE: ↓ S1 vs. S0 ***Percentage of cells with phosphatidylserine externalization and with DNA fragmentation***: no effect of supplementation or exercise ***Reactive oxygen species (ROS) production by unstimulated neutrophils***: no effect of supplementation or exercise ***ROS production by stimulated neutrophils, accumulation of neutral lipids:*** S0: ↑ ACUTE EXERCISE vs. REST S1: ↔ ACUTE EXERCISE vs. REST REST: ↑ S1 vs. S0 ACUTE EXERCISE: ↔ S1 vs. S0 ***Mitochondrial membrane potential***: S0: ↑ ACUTE EXERCISE vs. REST S1: ↔ ACUTE EXERCISE vs. REST REST: ↔ S1 vs. S0 ACUTE EXERCISE: ↓ S1 vs. S0
Michalczyk et al. 2018 [65]	Single-arm intervention	M/11	24.27 ± 2.6	4 weeks of low CHO diet (**LCD**; ~10% EI from CHO, ~31% PRO and 59% FAT) followed by 7 days of CHO loading (**Carbo-L**; 75% CHO, ~16% PRO, ~9% FAT). Conventional diet (**CD**; ~54% CHO, ~15% PRO, ~31% FAT) 1 month prior to the experiment	Measurements taken before (CD) and after 4-week LCD, as well as after the 7-day Carbo-L	**BODY MASS (BM) and BODY COMPOSITION** ***BM, fat-free mass (FFM, kg)***: ↔ LCD vs. CD, ↔ Carbo-L vs. CD ***Body fat (%), fat mass (FM, kg)***: ↓ LCD vs. CD, ↓ Carbo-L vs. CD **BLOOD VARIABLES** ***Triglycerides***: ↓ LCD vs. CD, ↑ Carbo-L vs. CD ***Glucose***: ↔ LCD vs. CD, ↑ Carbo-L vs. CD ***Total-, HDL-, and LDL-cholesterol, insulin, homeostasis model assessment-estimated insulin resistance (HOMA-IR)***: ↔ LCD vs. CD, ↔ Carbo-L vs. CD
Michalczyk et al. 2019 [66]	Single-arm intervention	M/15	23.5 ± 2.2	4 weeks of low CHO diet (**LCD**; ~10% EI from CHO, ~31% PRO and 59% fat) followed by 7 days of CHO loading (**Carbo-L**; 75% CHO, ~16% PRO, ~9% FAT). Conventional diet (**CD**; ~54% CHO, ~15% PRO, ~31% FAT) 1 month prior to the experiment	Measurements taken before (CD) and after 4-week LCD, as well as after the 7-day Carbo-L Measurements taken at REST and POST-EXERCISE (after the 30 s Wingate Anaerobic Test for lower limbs)	**BM and BODY COMPOSITION** ***BM***: ↓ LCD vs. CD, ↔ Carbo-L vs. CD, ↔ Carbo-L vs. LCD ***FFM (kg)***: ↔ LCD vs. CD, ↔ Carbo-L vs. CD, ↑ Carbo-L vs. LCD ***FM (%)***: ↓ LCD vs. CD, ↔ Carbo-L vs. CD, ↔ Carbo-L vs. LCD **ANAEROBIC PERFORMANCE** ***Peak power (PP), time to PP***: no differences between any condition ***Total work***: ↓ LCD vs. CD, ↔ Carbo-L vs. CD, ↑ Carbo-L vs. LCD **BLOOD ACID-BASE BALANCE** ***Lactate, pH*** at REST: ↓ LCD vs. CD, ↔ Carbo-L vs. CD, ↑ Carbo-L vs. LCD ***Lactate, pH*** at POST-EXERCISE: no differences between diets ***Bicarbonate*** at REST and POST-EXERCISE: no differences between diets ***β-HYDROXYBUTYRATE*** at REST: ↑ LCD vs. CD, ↔ Carbo-L vs. CD, ↓ Carbo-L vs. LCD **HORMONES** ***Testosterone: ↑*** LCD vs. CD, ↑ Carbo-L vs. CD, ↔ Carbo-L vs. LCD ***Growth hormone: ↑*** LCD vs. CD, ↔ Carbo-L vs. CD, ↓ Carbo-L vs. LCD ***Insulin: ↓*** LCD vs. CD; ↔ Carbo-L vs. CD, ↑ Carbo-L vs. LCD ***Cortisol***: no differences between diets
Ronghui 2015 [67]	Two-arm randomized parallel-group placebo-controlled study	M/10	-	30 days supplementation with 20 g whey PRO and 40 g oligosaccharides once every two days dissolved in 250 mL of whole milk 30 min before bed-time (**NUTR**) ***versus*** 250 mL of whole milk in the same manner (**CTRL**)	Measurements BEFORE and AFTER the supplementation period taken immediately after the incremental cycling test	***Haemoglobin, red blood cells, haematocrit*** at AFTER: ↑ NUTR vs. CTRL ***Mean corpuscular volume*** at AFTER: ↓ NUTR vs. CTRL ***Mean corpuscular volume*** in CTRL: ↑ AFTER vs. BEFORE
Shi 2005 [68]	Two-arm parallel-group placebo-controlled study	M/10	19–23	10 days (or 9) supplementation with Weichuan high-octane solid beverage (CHO content 100g∙L^−1^, 500 mL every day, **CHO-SOL**, *n* = 5) ***versus*** no-energy, colour-, appearance- and taste-matched placebo solution (**PLA**, *n* = 5) Preparations were ingested in three parts (at 7:00, 12:30, and 19:00), each part diluted in 200 mL of water	Measurements BEFORE and AFTER supplementation period, before (PRE-EXERCISE), after exercise (POST-EXERCISE), and 30 min POST-EXERCISE	***Δ in blood urea nitrogen***AFTER***supplementation between PRE- and POST-EXERCISE***: ↓ CHO-SOL vs. PLA ***Δ in CK activity*** AFTER ***supplementation between PRE- and POST-EXERCISE***: ↓ CHO-SOL vs. PLA ***Glucose*** at AFTER: PRE-EXERCISE: ↔ CHO-SOL vs. PLA POST-EXERCISE: ↑ CHO-SOL vs. PLA 30 min POST-EXERCISE: ↔ CHO-SOL vs. PLA ***Lactate*** at AFTER: PRE-EXERCISE, 5 and 10 min POST-EXERCISE: ↔ CHO-SOL vs. PLA
Taylor et al. 2016 [69]	Two-arm randomized placebo-controlled double-blind parallel group study	F/14	20 ± 2 (WP) 21 ± 3 (MD)	8 weeks of supplementation of 2 × 24 g whey PRO (WP; *n* = 8) ***versus*** maltodextrin (MD; *n* = 6) ingested immediately pre- and post-training (4 days/per week anaerobic and resistance training) during the pre-season part of training season	Measurements before (T1) and after (T2) supplementation (body composition, 1-repetition maximum [1RM] bench press [BP], 1RM leg press [LP], vertical jump [VJ], broad jump [BJ], 5-10-5 agility time)	**BM and BODY COMPOSITION** ***BM***: no group, time, or group ***x*** time interaction ***Lean mass*** at T1 and T2: ↔ WP vs. MD ***Lean mass*** in **WP**: ↑ T2 vs. T1, ***lean mass*** in **MD**: ↔ T2 vs. T1 ***Increase in lean mass (***T2–T1***)***: ↑ WP vs. MD ***Fat mass*** at T1 and T2: ↔ WP vs. MD ***Fat mass*** in **WP**: ↓T2 vs. T1 ***Fat mass*** in **MD**: ↔ T2 vs. T1 **PHYSICAL PERFORMANCE** ***1 RM BP, 1 RM LP, VJ, BJ, 5-10-5 agility drill*** at T1 and T2: ↔ WP vs. MD ***1 RM BP, 1 RM LP, VJ*** in **WP** and **MD**: ↑ T2 vs. T1 ***Increase in 1 RM BP***: ↑ WP vs. MD ***BJ*** in **WP**: ↑ T2 vs. T1, ***BJ*** in **MP**: ↔ T2 vs. T1 ***5-10-5 agility drill time*** in **WP**:↓ T2 vs. T1 and in **MD**: ↔ T2 vs. T1
Wilborn et al. 2013 [70]	Two-arm randomized double-blind parallel group study	F/16	20.0 ± 1.9 (WP) 21.0 ± 2.8 (CP)	8 week supplementation with 2 × 24 g whey PRO (**WP**; *n* = 8) ***versus*** 2 × 24 g casein PRO (**CP**; *n* = 8) 30 min before and immediately after training	Measurements before (T1) and after (T2) supplementation (body composition, 1RM BP, 1RM LP, VJ, BJ, 5-10-5 agility time)	**BM and BODY COMPOSITION** ***Body fat (%), FM (kg)*** in **WP** and **CP**: ↓ T2 vs. T1 ***Lean mass (kg)*** in **WP** and **CP**: ↑ T2 vs. T1 **BASKETBALL PERFORMANCE** ***1RM BP, 1 RM LP, VJ, BJ*** in **WP** and **CP**:↑ T2 vs. T1 ***5-10-5 agility drill time*** in **WP** and **CP**: ↓ T2 vs. T1

Abbreviations: AA, after awaking measurement; AD, after dinner measurement; BB, before breakfast measurement; BJ, broad jump; BD; before dinner measurement; BM, body mass; BP; bench press; BS, before sleep measurement; Carbo-L, carbohydrate loading diet; CD, conventional diet; CHO, carbohydrate; CHO-PRO, co-ingestion of carbohydrate and protein condition; CHO-SOL, ingestion of CHO solution condition; CK, creatine kinase; CP, casein protein; CTRL, study condition related to intake of placebo preparation; EPA, eicosapentaenoic acid; ESS, Epworth Sleepiness Scale; EI, energy intake; FFM, fat-free mass; FM, fat mass; GI, glycemic index; GIU, gastrointestinal upset; HR, heart rate; HGI, high glycemic index; IL-1ra, interleukin 1 ra; IL-1β, interleukin 1 beta; IL-4,interleukin 4; IL-6, interleukin 6; IL-8, interleukin 8; LCD, low carbohydrate diet; LGI, low glycemic index; LIST, Loughborough Intermittent Shuttle Test; LP, leg press, M, male; MD, maltodextrin; MDA, malonylodialdehyde; MS, muscle soreness; NUTR, study condition related to intake of protein and oligosaccharide enriched drink; PLA, placebo; PP, peak power; PRO, protein; ROS, reactive oxygen species; QR, quarter; RPE, rates of perceived exertion; TNF-α; tumor necrosis factor alfa; TT, time trial; VAS, visual analogue scale; VJ, vertical jump; VO_2_max, maximal oxygen uptake; WP, whey protein; 1RM, 1-repetition maximum. * value is median. ↑ significantly higher than comparator; ↔ no difference between comparators; ↓ significantly lower than comparator. ^†^ The UNDERLINED UPPER-CASE SCRIPTS in the descriptions of experimental procedures refer to the time point when evaluation measurements were taken.

## Supplementary Materials

The following supporting information can be downloaded at: https://www.mdpi.com/article/10.3390/nu15204484/s1, Table S1: PRISMA 2020 checklist (A) and PRISMA 2020 checklist for abstracts (B); Table S2: Basic characteristics of studies included in the systematic review; Supplementary Material S3: Risk of bias evaluation for observational cross-sectional studies related to habitual energy value and macronutrients intake (A,B) and habitual hydration strategies and hydration status (C,D); Supplementary Material S4: Risk of bias evaluation (A) and share of reasons for risk of biases (B) for randomized parallel group and cross-over interventional studies investigating dietary interventions related to hydration/dehydration; Supplementary Material S5: Risk of bias evaluation (A,B) for randomized parallel group and cross-over interventional studies and risk of bias evaluation for single-arm interventional (C,D) investigating dietary interventions related to manipulation in CHO/PRO/FAT intake; Supplementary Material S6: Risk of bias evaluation for observational cross-sectional (A,B), single-arm interventional (C,D), and randomized cross-over interventional (E) studies related to eating behaviours/nutritional knowledge.
