# Supplementary material for: Practical Application and Methodological Considerations on the Basics of Sports Nutrition in Basketball: A Comprehensive Systematic Review of Observational and Interventional Studies
Source: Nutrients. 2023 Oct 23;15(20):4484. doi: 10.3390/nu15204484 (PMC10610293; doi:10.3390/nu15204484)
Supplement: Supplementary file 1 [file nutrients-15-04484-s001.zip › nutrients-2613531-supplementary/Supplementary material S3.pdf]

Supplementary material S3. Risk of bias evaluation for observational cross-sectional studies related to habitual energy value and macronutrients intake (A-B) and habitual hydration strategies and hydration status (C-D)

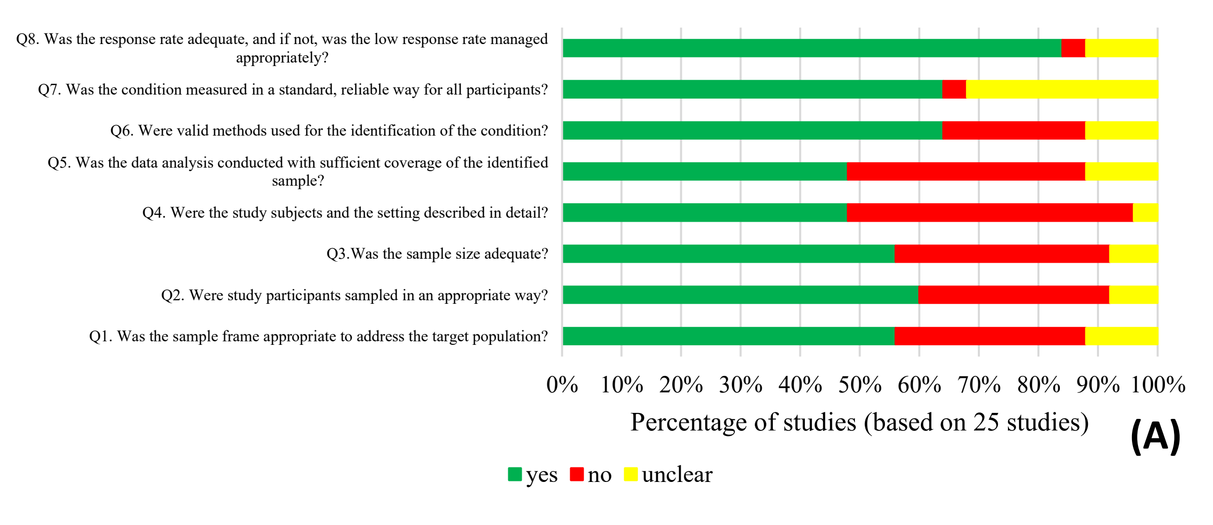

References	Questions							
	Q1	Q2	Q3	Q4	Q5	Q6	Q7	Q8
Baranauskas et al. 2011	no	no	no	no	no	no	unclear	yes
Baranauskas et al. 2013	yes	yes	yes	yes	no	no	unclear	yes
Baranauskas et al. 2020	yes	yes	yes	yes	yes	yes	yes	yes
Dzimbova 2020	no	no	no	no	no	yes	yes	yes
Eskici & Ersoy 2016	yes	yes	yes	no	yes	no	unclear	yes
Ferro et al. 2017	yes	yes	yes	yes	yes	yes	yes	yes
Gacek 2022	yes	yes	yes	yes	yes	no	yes	yes
Grams et al. 2016	yes	yes	yes	no	yes	yes	yes	yes
Hickson et al. 1986	no	no	no	no	no	yes	yes	yes
Hickson et al. 1990	no	no	no	no	no	yes	yes	yes
Kostopoulos et al. 2017	unclear	yes	unclear	yes	no	yes	yes	yes
Leinus & Ööpik 1998	no	no	no	no	no	yes	yes	yes
Nepocatych et al. 2017	no	unclear	no	no	no	yes	yes	unclear
Nikić et al. 2014	yes	yes	yes	no	unclear	yes	yes	yes
Nowak et al. 1988	no	no	no	no	no	yes	yes	yes
Papandreou et al. 2007	no	no	no	no	yes	unclear	unclear	yes
Quintas et al. 2003	unclear	unclear	unclear	yes	unclear	no	no	yes
Schröder et al. 2004	yes	yes	yes	yes	unclear	no	unclear	no
Shimizu et al. 2019	yes	yes	yes	yes	yes	yes	yes	yes
Silva et al. 2012	yes	yes	yes	yes	yes	unclear	unclear	yes
Silva et al. 2013	yes	yes	yes	yes	yes	yes	yes	yes
Silva et al. 2017	yes	yes	yes	unclear	yes	yes	yes	yes
Toti et al. 2021	yes	yes	yes	yes	yes	yes	unclear	unclear
Toti et al. 2022	yes	yes	yes	yes	yes	yes	yes	yes
Zanders et al. 2021	unclear	no	no	yes	no	unclear	unclear	unclear

Legend

yes no unclear

(B)

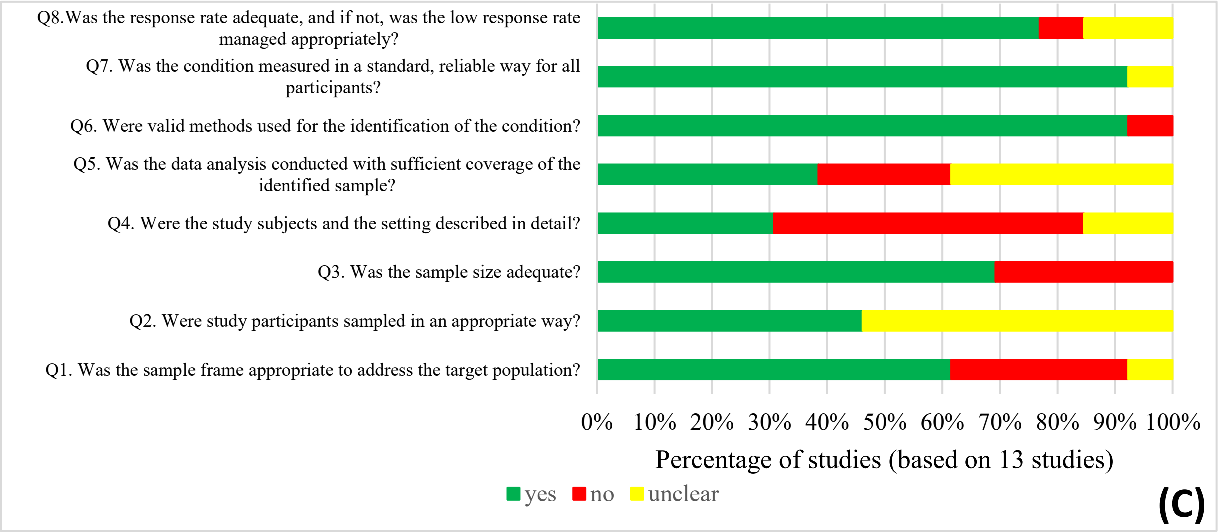

(C)

References	Questions							
	Q1	Q2	Q3	Q4	Q5	Q6	Q7	Q8
Abassi et al. 2021	no	unclear	no	yes	unclear	yes	yes	yes
Arnaoutis et al. 2015	no	unclear	no	no	unclear	yes	yes	yes
Baker et al. 2007b	unclear	unclear	yes	yes	no	yes	yes	yes
Barnes et al. 2019	yes	yes	yes	no	no	yes	yes	yes
Brandenburg & Gaetz 2012	no	unclear	no	no	unclear	yes	yes	yes
Broad et al. 1996	yes	yes	yes	no	yes	yes	yes	yes
Heishman et al. 2021	yes	unclear	yes	no	yes	yes	yes	unclear
Logan-Sprenger & McNaughton 2019	yes	yes	yes	unclear	yes	yes	yes	yes
Osterberg et al. 2009	yes	yes	yes	unclear	unclear	yes	yes	unclear
Schröder et al. 2004	yes	yes	yes	yes	unclear	no	unclear	no
Taim et al. 2021	yes	unclear	no	no	no	yes	yes	yes
Thigpen et al. 2014	yes	unclear	yes	no	yes	yes	yes	yes
Vukašinović-Vesić et al. 2015	yes	yes	yes	yes	yes	yes	yes	yes

Legend: yes no unclear

(D)
