# Supplementary material for: Practical Application and Methodological Considerations on the Basics of Sports Nutrition in Basketball: A Comprehensive Systematic Review of Observational and Interventional Studies
Source: Nutrients. 2023 Oct 23;15(20):4484. doi: 10.3390/nu15204484 (PMC10610293; doi:10.3390/nu15204484)
Supplement: Supplementary file 1 [file nutrients-15-04484-s001.zip › nutrients-2613531-supplementary/Supplementary material S4.pdf]

Supplementary material S4. Risk of bias evaluation (A) and share of reasons for risk of biases (B) for randomized parallel group and cross-over interventional studies investigating dietary interventions related to hydration/dehydration

References	Study design	Randomisation process	Bias arising from period and carryover effects	Deviations from the intended interventions	Missing outcome data	Measurement of the outcome	Selection of the reported result	Overall
Baker et al. 2007a	randomized cross-over	!	+	-	+	!	-	-
Baker et al. 2007b	randomized cross-over	!	+	-	+	!	-	-
Carvalho et al. 2011	randomized cross-over	!	+	+	+	!	!	!
Dougherty et al. 2011	randomized cross-over	+	+	-	+	!	-	-
Hoffman et al. 1995	randomized cross-over	!	+	-	+	!	!	-
Hoffman et al. 2012	randomized cross-over	+	!	!	+	!	-	-
Louis et al. 2018	randomized cross-over	!	!	-	+	!	!	-
Minehan et al. 2002	randomized cross-over	!	+	-	+	!	!	-
Taim et al. 2021	randomized parallel group	!	not applicable	!	+	!	!	!

Legend:

+ Low risk   ! Some concerns   - High risk

(A)

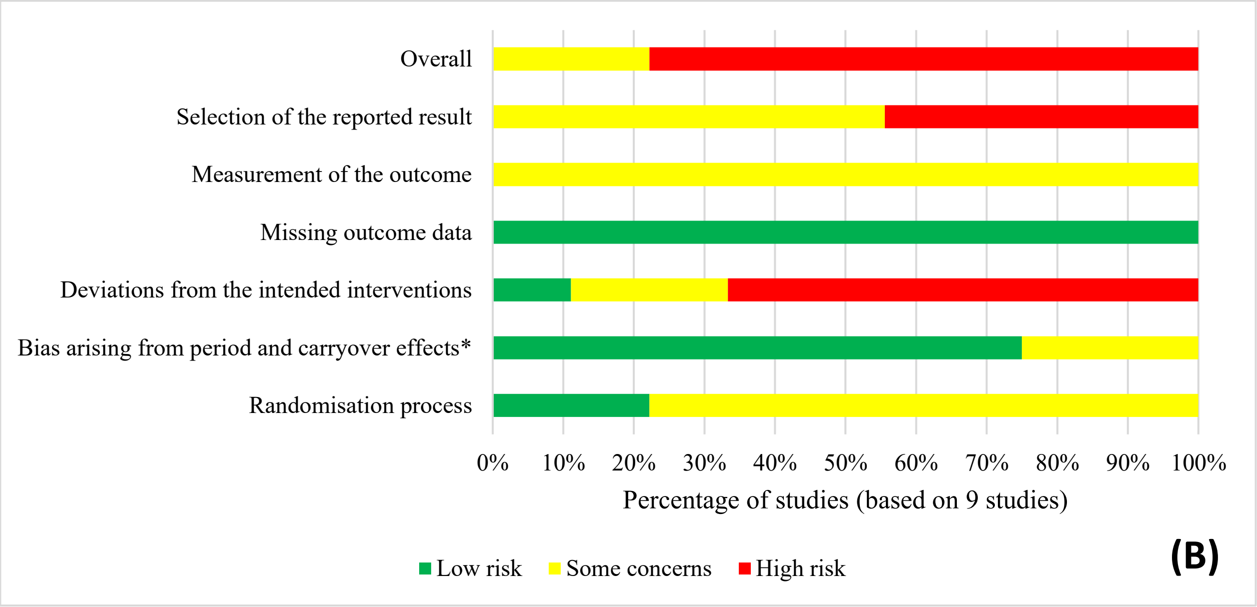

*applicable for cross-over designs
