# Supplementary material for: Practical Application and Methodological Considerations on the Basics of Sports Nutrition in Basketball: A Comprehensive Systematic Review of Observational and Interventional Studies
Source: Nutrients. 2023 Oct 23;15(20):4484. doi: 10.3390/nu15204484 (PMC10610293; doi:10.3390/nu15204484)
Supplement: Supplementary file 1 [file nutrients-15-04484-s001.zip › nutrients-2613531-supplementary/Supplementary material S5.pdf]

Supplementary material S5. Risk of bias evaluation (A-B) for randomized parallel group and cross-over interventional studies and risk of bias evaluation for single-arm interventional (C-D) investigating dietary interventions related to manipulation in CHO/PRO/FAT intake

References	Study design	Randomisation process	Period and carryover effects	Deviations from the intended interventions	Missing outcome data	Measurement of the outcome	Selection of the reported result	Overall
Afman et al. 2014	randomized cross-over	!	+	!	+	!	!	!
Daniel et al. 2019	randomized cross-over	!	!	!	+	!	!	!
Gentle et al. 2014	randomized cross-over	!	!	-	+	!	!	-
Ghiasvand et al. 2014	randomized parallel group	!	not applicable	-	+	!	!	-
Ho et al. 2018	randomized cross-over	!	+	-	+	!	!	-
Ronghui 2015	randomized parallel group	!	not applicable	-	+	!	!	-
Shi 2005	randomized parallel group	!	not applicable	-	+	!	-	-
Taylor et al. 2016	randomized parallel group	!	not applicable	+	+	!	!	!
Wilborn et al. 2013	randomized parallel group	!	not applicable	!	+	!	!	!

Legend:

+ Low risk ! Some concerns - High risk

(A)

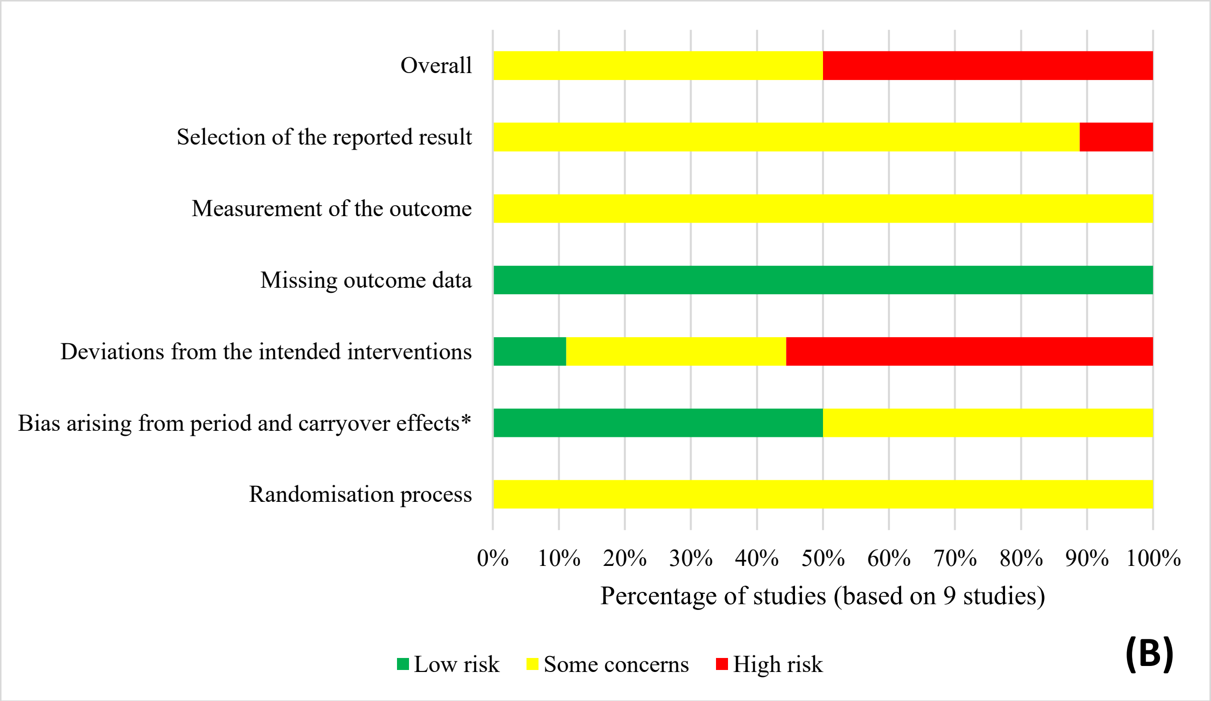

*applicable for cross-over designs

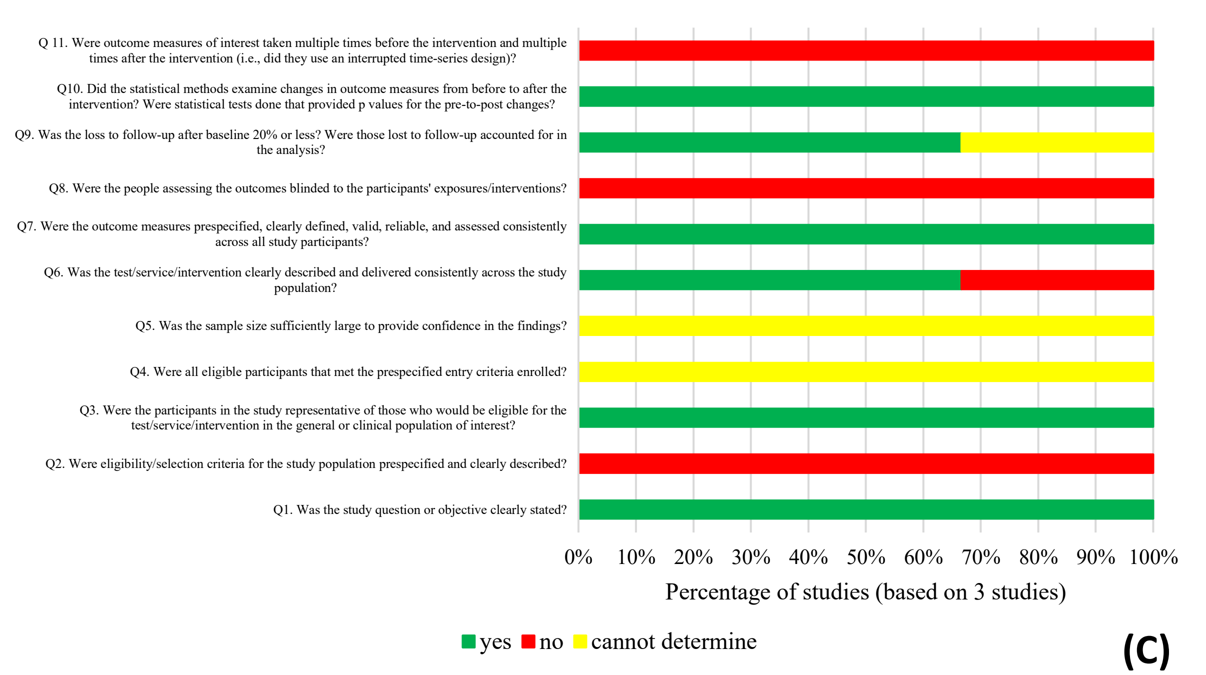

(C)

References	Questions										
	Q1	Q2	Q3	Q4	Q5	Q6	Q7	Q8	Q9	Q10	Q11
Marques et al. 2015	yes	no	yes	cannot determine	cannot determine	no	yes	no	yes	yes	no
Michalczyk et al. 2018	yes	no	yes	cannot determine	cannot determine	yes	yes	no	cannot determine	yes	no
Michalczyk et al. 2019	yes	no	yes	cannot determine	cannot determine	yes	yes	no	yes	yes	no

Legend

yes	no	cannot determine

(D)
