# Supplementary material for: Practical Application and Methodological Considerations on the Basics of Sports Nutrition in Basketball: A Comprehensive Systematic Review of Observational and Interventional Studies
Source: Nutrients. 2023 Oct 23;15(20):4484. doi: 10.3390/nu15204484 (PMC10610293; doi:10.3390/nu15204484)
Supplement: Supplementary file 1 [file nutrients-15-04484-s001.zip › nutrients-2613531-supplementary/Supplementary material S6.pdf]

Supplementary material S6. Risk of bias evaluation for observational cross-sectional (A-B), single-arm interventional (C-D) and randomized cross-over interventional (E) studies related to eating behaviours/nutritional knowledge

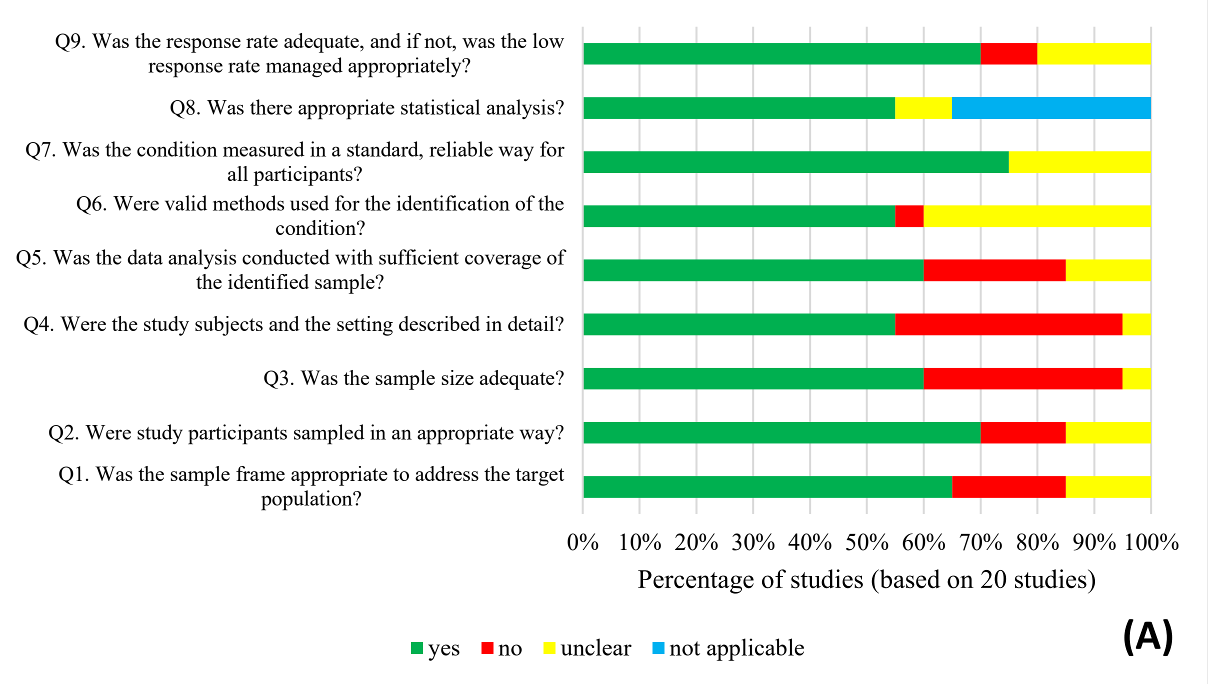

References	Questions								
	Q1	Q2	Q3	Q4	Q5	Q6	Q7	Q8	Q9
Boumesleh et al. 2021	yes	yes	yes	yes	yes	unclear	yes	yes	yes
del Mar Bibiloni et al. 2018	yes	yes	yes	unclear	yes	unclear	yes	yes	yes
Escribano-Ott et al. 2022	yes	unclear	yes	yes	yes	yes	yes	yes	yes
Eskici & Ersoy 2016	yes	yes	yes	yes	yes	yes	yes	not applicable	yes
Ferro et al. 2017	yes	yes	yes	no	yes	yes	yes	yes	yes
Gacek & Wojtowicz 2021	yes	yes	yes	yes	yes	yes	yes	yes	yes
Gorrell et al. 2021	no	unclear	no	no	no	yes	yes	yes	unclear
Kampouri et al. 2019	yes	yes	unclear	yes	yes	yes	yes	yes	unclear
Mavra et al. 2014	yes	yes	yes	no	yes	unclear	unclear	yes	yes
Michou & Costarelli 2011	yes	yes	yes	yes	yes	yes	yes	unclear	yes
Monthuy-Blanc et al. 2012	unclear	yes	no	yes	yes	yes	yes	yes	no
Musaiger & Ragheb 1994	unclear	yes	no	no	unclear	unclear	unclear	not applicable	yes
Nikić et al. 2014	yes	yes	yes	no	unclear	unclear	yes	not applicable	yes
Nowak et al. 1988	no	no	no	no	no	yes	yes	not applicable	yes
Sánchez-Diaz et al. 2021	no	no	no	yes	yes	unclear	yes	not applicable	yes
Schröder et al. 2004	yes	yes	yes	yes	unclear	no	unclear	not applicable	no
Szczepańska & Spałkowska 2012	yes	yes	yes	no	no	unclear	unclear	not applicable	yes
Toti et al. 2022	yes	yes	yes	yes	yes	yes	yes	yes	yes
Wells et al. 2015	no	unclear	no	no	no	yes	yes	yes	unclear
Zanders et al. 2021	unclear	no	no	yes	no	unclear	unclear	unclear	unclear

Legend

yes	no	unclear	not applicable

(B)

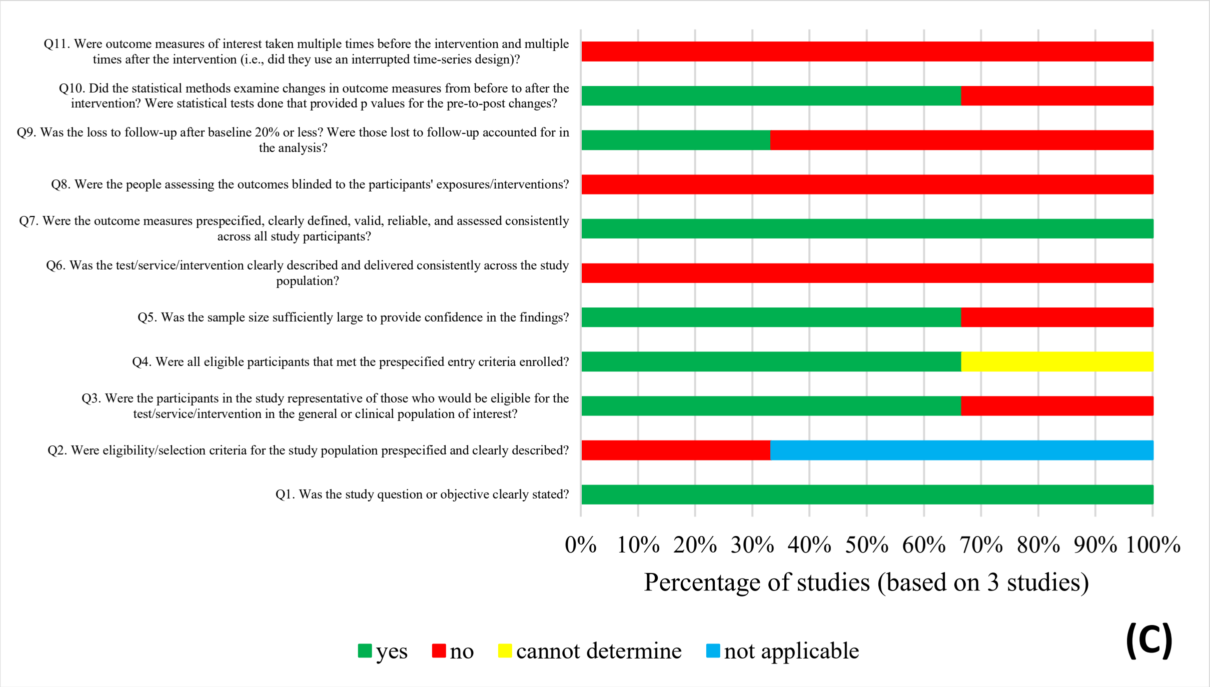

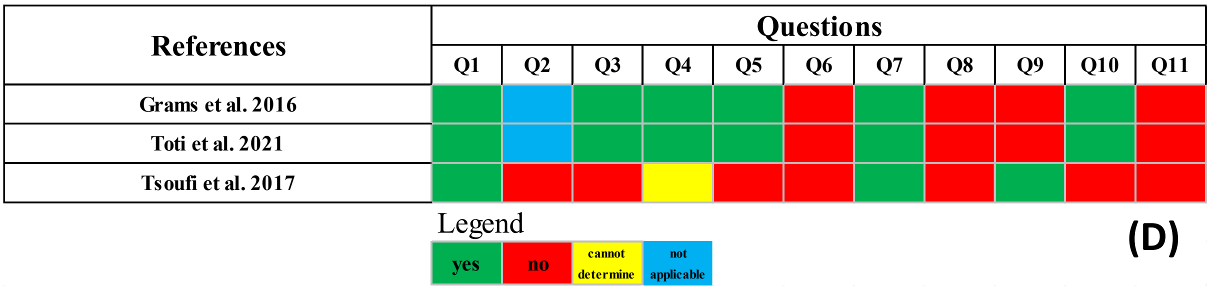

References	Study design	Randomisation process	Period and carryover effects	Deviations from the intended interventions	Missing outcome data	Measurement of the outcome	Selection of the reported result	Overall
Čabarkapa et al. 2020	randomized cross-over	!	!	!	+	!	!	!

Legend:
+ Low risk
! Some concerns
- High risk

(E)
