# Supplementary material for: Practical Application and Methodological Considerations on the Basics of Sports Nutrition in Basketball: A Comprehensive Systematic Review of Observational and Interventional Studies
Source: Nutrients. 2023 Oct 23;15(20):4484. doi: 10.3390/nu15204484 (PMC10610293; doi:10.3390/nu15204484)
Supplement: Supplementary file 1 [file nutrients-15-04484-s001.zip › nutrients-2613531-supplementary/Supplementary table S2.pdf]

Supplementary table S2. Basic characteristics of studies included in the systematic review

No.	Authors	Location	Study design	Sample size	Gender	Age (years)	Level of training
**Observational studies**							
1.	Arnaoutis et al. 2015	-	cross-sectional	12	males	15.5 ± 0.5	athletes competing in national and international championships
2.	Baranauskas et al. 2013	Lithuania	cross-sectional	52	males (*n* = 39)<br>females (*n* = 13)	18.6 ± 1.8<br>16.1 ± 0.5	Lithuanian Olympic Team
3.	Baranauskas et al. 2020	Lithuania	cross-sectional	14	females	26.4 ± 4.5	high-performance **deaf** women's players
4.	Barnes et al. 2019	-	retrospective analysis	196	males<br>females	23 ± 5	-
5.	Branderburg and Gaetz 2012	Canada	cross-sectional	17	females	24.2 ± 3	national team
6.	Broad et al. 1996	Australia	cross-sectional	31	males (*n* = 19)<br>females (*n* = 12)	16 – 18	players training at Australian Institute of Sport
7.	Boumosleh et al. 2021	Lebanon	cross-sectional	178	males (*n* = 126)<br>females (*n* = 52)	28.17 ± 4.42<br>25.13 ± 4.28	1st Division Lebanese Championship
8.	Davis et al. 2021	USA	cross-sectional	119	males	23.8 ± 2.0	players in the National Basketball Association minor league
9.	del Mar Bibiloni et al. 2018	Spain	cross-sectional	183	males (*n* = 96)<br>females (*n* = 87)	21.7 (20.4 ± 25.3)*<br>22.4 (20.1 ± 24.6)*	amateur players
10.	Dzimbova 2020	Bulgaria	cross-sectional	16	males, females	15.4 ± 1.2	-
11.	Escribano-Ott et al. 2022	Spain	cross-sectional	104	males (*n* = 49)<br>females (*n* = 55)	15 – 18	children involved in sport professionally under 18 years (*n* = 69), professional adult players (*n* = 21), non-professional players (*n* = 14)

No.	Authors	Location	Study design	Sample size	Gender	Age (years)	Level of training
12.	Eskici and Ersoy 2016	Turkey	cross-sectional	22	females	25.5 ± 7.2	national **wheelchair** team
13.	Ferro et al. 2017	Spain	cross-sectional	11	males	30 ± 6	national **wheelchair** team
14.	Gacek and Wojtowicz 2021	Poland	cross-sectional	165	males	23.44 ± 3.74	professionally training basketball (1st and 2nd leagues, as well as the top league)
15.	Gacek 2022	Poland	cross-sectional	48	males	26.6 ± 4.5	professional players
16.	Gorrell et al. 2021	USA	cross-sectional	16	males	18 – 26	10 top-ranked National Collegiate Athletic Association (NCAA) schools in USA
17.	Heishman et al. 2021	USA	retrospective longitudinal analysis	15	males	20.4 ± 1.7	NCAA Division I
18.	Hickson et al. 1986	USA	cross-sectional	13	females	19.4 ± 0.3	intercollegiate team
19.	Hickson et al. 1990	USA	cross-sectional	12	males	16.4 ± 0.7	high-school varsity players
20.	Kampouri et al. 2019	Greece	cross-sectional	53	females	24.30 ± 6.4	elite players
21.	Kostopoulos et al. 2017	Greece	cross-sectional	18	-	24 ± 4	A1 division (1st and 2nd division)
22.	Leinus and Ööpik 1998	Estonia	cross-sectional	14	males (n = 7)<br>females (n = 7)	21.1 ± 2.6<br>20.6 ± 1.9	4-6 y of training, 90 min/day, 5-6 times/week<br>8-10 y of training, 90 min/day, 2-4 times/week
23.	Logan-Sprenger and McNaughton 2020	Canada	cross-sectional	11	females	18 – 41	national wheelchair team
24.	Mavra et al. 2014	Croatia	cross-sectional	153	females	21.90 ± 4.89 (1st)<br>17.30 ± 2.85 (2nd)	Players from the 1st (n = 79) and the 2nd (n = 74) national league

No.	Authors	Location	Study design	Sample size	Gender	Age (years)	Level of training
25.	Michou and Costarelli 2011	Greece	cross-sectional	74	females	24.92 ± 3.81	national and international level
26.	Monthuy-Blanc et al. 2012	France	cross-sectional	41	females	13.59 ± 1.32	non-elite players
27.	Musaiger and Ragheb 1994	Bahrain	cross-sectional	39	-	-	1st class clubs
28.	Nepocatych et al. 2017	USA	cross-sectional	10	females	18 – 22	NCAA Division I
29.	Nikić et al. 2014	Serbia	cross-sectional	57	males	15.6 ± 0.9	elite junior
30.	Nowak et al. 1988	USA	cross-sectional	26	males (n = 16)<br>females (n = 10)	18.9 ± 1.29<br>19.4 ± 0.97	National Association of Intercollegiate Athletics – Division II
31.	Osterberg et al. 2009	USA	cross-sectional	29	males	-	National Basketball Association
32.	Papandreou et al. 2007	Greece	cross-sectional	21	males (n = 8)<br>females (n = 13)	20 ± 4<br>25 ± 5	professional players
33.	Quintas et al. 2003	Spain	cross-sectional	26	females	17.2 ± 2.1	-
34.	Sánchez-Diaz et al. 2021	Spain	cross-sectional	23	males (n = 13)<br>females (n =10)	13.5 ± 0.3<br>12.7 ± 0.5	highest competitive level for the U-14 category
35.	Schröder et al. 2004	Spain	cross-sectional	50	males	25.1 ± 4.0	1st Spanish Basketball League
36.	Shimizu et al. 2019	Japan	cross-sectional data	13	females	28.9 ± 8.1	national wheelchair team
37.	Silva et al. 2012	Portugal	longitudinal approach (~34 weeks)	9	males (n = 7)<br>females (n = 2)	16.0 ± 0.5<br>16.3 ± 0.5	junior national team
38.	Silva et al. 2013	Portugal	cross-sectional	19	males (n = 12)<br>females (n = 7)	17.0 ± 0.7<br>16.9 ± 0.7	junior national team
39.	Silva et al. 2017	-	8 months observation	24	-	-	-

No.	Authors	Location	Study design	Sample size	Gender	Age (years)	Level of training
40.	Szczepańska and Spałkowska 2012	Poland	cross-sectional	107	males (n = 89)<br>females (n = 18)	17 – 33	-
41.	Thigpen et al. 2014	USA	cross-sectional	22	males (n = 11)<br>females (n = 11)	21 ± 1<br>19 ± 1	NCAA Division II
42.	Toti et al. 2021	Italy	cross-sectional	15	males	28.5 ± 1.5	national wheelchair team
43.	Vukasinović-Vesić et al. 2015	International	cross-sectional	96	males	19 ± 0.79	elite players during official International Basketball Federation (FIBA) Europe U20 Championship
44.	Wells et al. 2015	USA	cross-sectional	8	females	-	varsity players from Campbell University team
45.	Zanders et al. 2021	USA	cross-sectional	13	females	19.8 ± 1.3	NCAA Division II
	**Experimental studies**						
46.	Abbasi et al. 2021	USA	controlled laboratory study/dietary counseling intervention	10	females	-	NCAA Division II
47.	Afman et al. 2014	United Kingdom	randomized, counterbalanced crossover placebo-controlled study	10	males	20 ± 1	from university to international level competition
48.	Baker et al. 2007a	USA	six arms randomized cross-over placebo controlled trial (double blind with respect to euhydration trials)	11	males	21 ± 3	-

No.	Authors	Location	Study design	Sample size	Gender	Age (years)	Level of training
49.	Baker et al. 2007b	USA	six-arm randomized cross-over placebo controlled trial (double blind with respect to euhydration trials)	17	males	21.1 ± 2.4	highly skilled players ranged from high school (*n* = 9) to college (Division III, *n* = 4; Division I, *n* = 4)
50.	Baranauskas et al. 2011	Lithuania	single arm interventional study	10	females	16.2 ± 0.4	Lithuanian Olympic Sport Centre players
51.	Carvalho et al. 2011	Portugal	three-arm randomized cross-over trial	12	males	14.8 ± 0.45	U-15 national team
52.	Čabarkapa et al. 2020	USA	two-arm cross-over design	18	males	27.5 ± 10.6	considerable amount of playing experience
53.	Daniel et al. 2019	Brazil	two-arm randomized cross-over design	9	males	18.0 ± 0.7	high-performance
54.	Dougherty et al. 2006	USA	double blind randomized placebo-controlled cross-over trial	15	males	13.5 ± 1.3	first-team member of either their school or district Amateur Athletic Union team or both in the central Pennsylvania region
55.	Gentle et al. 2014	New Zealand	two-arm randomized cross-over design	10	males	22 ± 2	well-trained
56.	Ghiasvand et al. 2010	Iran	randomized double blind placebo-controlled clinical trial	34	males	24 (17 – 35)[ǂ]	well-trained

No.	Authors	Location	Study design	Sample size	Gender	Age (years)	Level of training
57.	Grams et al. 2016	Spain	interventional study (dietary counseling)	17	males	30 (21 – 40)	national wheelchair team
58.	Ho et al. 2018	Taiwan	two-arm randomized, placebo-controlled counterbalanced cross-over design	15	-	18 – 20	Division I collegiate players
59.	Hoffman et al. 1995	Israel	two-arm balanced cross-over design	10	males	17.3 ± 0.9	players in regional youth team
60.	Hoffman et al. 2012	USA	four-arm double blind cross-over design	10	females	21.2 ± 1.6	NCAA Division I
61.	Louis et al. 2018	-	two-arm randomized cross-over trial	9	males	16.2 ± 0.7	U-18 of the basketball academy of the National team
62.	Marques et al. 2015	Brazil	single arm study	8	males	33.8 ± 8.3	wheelchair athletes participating in national championships
63.	Minehan et al. 2002	Australia	three-arms randomized cross-over design	15	males (n = 8) females (n = 7)	-	players training at Australian Institute of Sport
64.	Michalczyk et al. 2018	Poland	single-arm dietary intervention	11	males	24.27 ± 2.6	1st Division of the Polish Basketball League
65.	Michalczyk et al. 2019	Poland	single-arm dietary intervention	15	males	23.5 ± 2.2	1st Division of the Polish Basketball League
66.	Ronghui 2015	China	two-arm randomized parallel	10	-	-	university professional players

No.	Authors	Location	Study design	Sample size	Gender	Age (years)	Level of training
			group placebo-controlled study				
67.	Shi 2005	China	two-arm parallel-group study	10	males	19 – 23	CUBA athletes
68.	Taim et al. 2021	Singapore	parallel group randomized between-subject design	18	males	23.4 ± 1.4 (experimental)<br>22.8 ± 1.2 (control)	national varsity league
69.	Taylor et al. 2016	USA	two-arm randomized placebo-controlled double blind parallel group study	14	females	20 ± 2 (experimental)<br>21 ± 3 (control)	NCAA Division III
70.	Toti et al. 2021	Italy	interventional study (dietary counseling)	37	males (n = 16)<br>males (n = 12)<br>females (n = 9)	27 (24 – 31)<br>19 (18 – 21)<br>26 (19 – 30)	national wheelchair team
71.	Tsoufi et al. 2017	Greece	interventional study (dietary counseling)	15	males	-	elite players
72.	Wilborn et al. 2013	USA	Two-arm randomized double blind parallel group study	16	females	20.0 ± 1.9 (whey protein)<br>21.0 ± 2.8 (casein protein)	NCAA Division III

Abbreviations: NCAA, National Collegiate Athletic Association. Note: * values are median (interquartile range); ‡ values are median (range)
